# Characters related to higher starch accumulation in cassava storage roots

**DOI:** 10.1038/srep19823

**Published:** 2016-02-19

**Authors:** You-Zhi Li, Jian-Yu Zhao, San-Min Wu, Xian-Wei Fan, Xing-Lu Luo, Bao-Shan Chen

**Affiliations:** 1 State Key Laboratory for Conservation and Utilization of Subtropical Agro-bioresources; Key Laboratory of Ministry of Education for Microbial and Plant Genetic Engineering; College of Life Science and Technology, Guangxi University; 100 Daxue Road, Nanning, Guangxi 530004, P. R. China

## Abstract

Cassava (*Manihot esculenta*) is valued mainly for high content starch in its roots. Our understanding of mechanisms promoting high starch accumulation in the roots is, however, still very limited. Two field-grown cassava cultivars, Huanan 124(H124) with low root starch and Fuxuan 01(F01) with high root starch, were characterised comparatively at four main growth stages. Changes in key sugars in the leaves, stems and roots seemed not to be strongly associated with the final amount of starch accumulated in the roots. However, when compared with H124, F01 exhibited a more compact arrangement of xylem vascular bundles in the leaf axils, much less callose around the phloem sieve plates in the stems, higher starch synthesis-related enzymatic activity but lower amylase activity in the roots, more significantly up-regulated expression of related genes, and a much higher stem flow rate (SFR). In conclusion, higher starch accumulation in the roots results from the concurrent effects of powerful stem transport capacity highlighted by higher SFR, high starch synthesis but low starch degradation in the roots, and high expression of sugar transporter genes in the stems. A model of high starch accumulation in cassava roots was therefore proposed and discussed.

Cassava (*Manihot esculenta*) is an important staple food crop in many developing countries, especially in tropical, intertropical, and sub-Saharan regions^1^. It is valued for the root starch, which accounts for about 80% of the dry matter^2^. Cassava currently attracts great attention worldwide because its starch is the most important source of raw materials for fuel ethanol production^3^,^4^. Enhancing the starch content in the roots is a core breeding goal but will rely greatly on engineering approaches because this crop is not particularly amenable to genetic improvement through sexual crosses^5^. However, improving cassava through plant biotechnology is extremely challenging because it is very difficult to find significant phenotypes in breeding because of the limited knowledge of inheritance traits^6^.

In plants, starch is synthesised in the leaves with sucrose as one of the important sugar sources during the day, mobilized and then degraded for energy metabolism requirements at night^7^,^8^. The committed starch biosynthesis pathway is conserved almost in all green plants^2^,^9^,^10^,^11^,^12^. However, the process of starch accumulation is very complex and varies greatly with plant species^13^,^14^.

Starch biosynthesis in cassava, as in other plants, involves several key enzymes: ADP-glucose pyrophosphorylase (AGPase), starch synthases, branching enzymes, and debranching enzymes^7^,^11^. So far, genes encoding soluble starch synthases III, starch phosphorylase, and 1,4-α-glucan branching enzyme have been identified and cloned^15^. Large-scale analysis of gene expression has been conducted in different cassava cultivars^1^,^15^,^16^,^17^,^18^,^19^, and provided a large number of genes, of which some are potentially related to starch biosynthesis and accumulation or to post-harvest physiological deterioration of the roots. In fact, it is impossible to fully explain high starch accumulation in the starch storage roots of cassava if events are observed at only one growth stage. This is, at least in part, because field-grown cassava plants usually start from propagation of stem cuttings/stakes, and their growth and development undergo four distinct stages of emergence of sprouting from stem stakes, formation of root system, root bulking, and carbohydrate translocation to roots^20^.

The following four key issues have puzzled cassava scientists because of the lack of solid experimental evidence: (1) whether the high starch accumulation in starch storage roots is associated with strong transport capacity of the stems; (2) whether high starch accumulation in the starch storage roots is related to lower efficiency of starch degradation; (3) whether only non-reducing sugars can move by long-distance transportation hrough the stem phloem; and/or (4) whether sugar transport correlates to the degree of starch accumulation in the starch storage roots.

Huanan 124 (H124) and Fuxuan 01 (F01) are two of the main cassava cultivars in Guangxi, China. The former has the root starch content lower than the latter^21^. Usually, the growth and development of field-grown cassava plants grown from stem stakes in the cassava-growing area in Guangxi can be divided empirically into the four key growth stages of (1) ‘seedling’ (a local customary terms by farmers to describe the young cassava plants that start from the stem stakes) 50 ± 5 days after planting (DAP), (2) formation of root system (110 ± 5 DAP), (3) visible root bulking (180 ± 5 DAP), and (4) starch maturity (240 ± 5 DAP). The goal of this study is to reveal characters related to the formation of high starch content in storage roots through analyses of differences in plants of cassava cultivars H124 and F01 (which started from stem cuttings and were filed-grown) and by examining starch synthesis and degradation, sugar content, structure of the vascular bundles, stem flow rate (SFR), and gene expression at the four key growth stages mentioned above.

## Results

Unless we specify otherwise, roots that are mentioned as being at the two stages of ‘seedling’ and formation of root system refer to fibrous roots, and roots at the two stages of root bulking and root maturity refer to the storage roots.

### Starch content in cassava tissues

**Figure 1:**
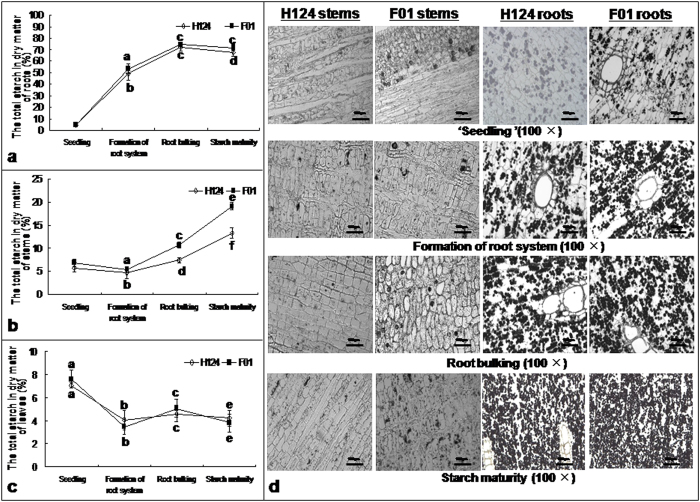
Starch content in the dry matter of the cassava roots (a), cassava stems (b) and cassava leaves (c); and starch granule density (d) in stems and roots of cassava. Plants of the same size were chosen. For analysis of starch content, leaf tissue samples (not including petioles) from the sixth leaf down from the top of each plant were used. Root tissue samples were taken from whole fibrous roots at the first two growth stages of ‘seedling’ and formation of root system, or from the tissues from transversely sectioned middle sections of the storage roots. For observation of starch granule density in the cells, the tissues were transversely sectioned through tissue paraffin section methods, where the thickness of tissue slices was 10 μm. The tissue slices were stained in I_2_ (2%)-KI(1%) solution and then photographed. Each datum in column figures was the mean ± standard deviation (SD) from three individual plants. Different letters on the columns indicate the statistical difference at a level of *p* < 0.05. F01, cassava cultivar Fuxuan 01. H124, cassava cultivar Huanan 124.

The total starch in F01 roots was significantly (p < 0.05) higher than that in H124 roots after formation of root systems (Fig. 1a), coinciding with the previous report^21^. The total starch in F01 stems was much higher than that in H124, especially after formation of root system (Fig. 1b). However, the starch contents in leaves of these two cultivars showed no significant (p < 0.05) difference at the four growth stages (Fig. 1c). It should be noted that the starch content increased significantly (p < 0.05) in roots (Fig. 1a) and stems (Fig. 1b) but declined significantly (p < 0.05) in leaves (Fig. 1c) with the growth of cassava.

To observe the difference between cells of H124 and F01 in starch granule density/number, we carried out the paraffin section-based tissue staining. Consequently, the starch granules were much smaller but might form in stem and root cells at ‘seedling’ stage. However, the density of the starch granules was much higher in root cells of F01 than in root cells of H124 (Fig. 1d). These results directly supported the difference we found in starch contents between roots and stems of H124 and F01 as the plants grew (Fig. 1a,b).

### Activities of starch synthesis- and degradation-related enzymes

Starch biosynthesis capacity depends on the activities of such key enzymes as starch synthase and AGPase. The latter is located in a rate-limiting step of the starch synthesis pathway^22^.

**Figure 2:**
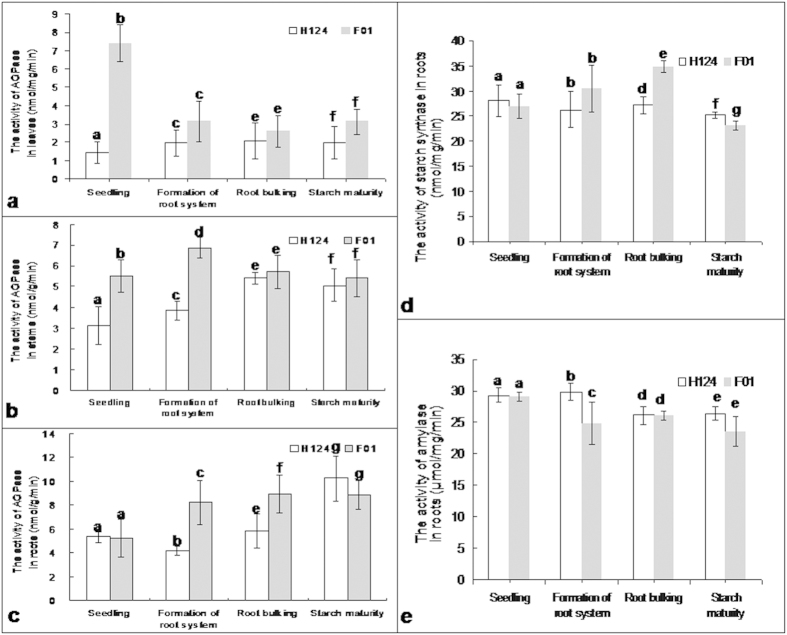
The AGPase activity in leaves (a), stems (b) and roots (c); and the starch synthase activity (d) and amylase activity (e) in roots of cassava cultivars H14 and F01. Plants of the same size were chosen. Leaf tissue samples (not including petioles) from the sixth leaf down from the top of each plant were used. The roots were fibrous roots at the first two growth stages of ‘seedling’ and formation of root system, and the storage roots at the other two growth stages of root bulking and starch maturity. For analysis, root tissues were taken from whole fibrous roots or from the middle sections of the storage roots. Each datum was the mean ± SD from the data of three individual plants. Different letters on the columns indicate the statistic difference at a level of *p* < 0.05. F01, cassava cultivar Fuxuan 01. H124, cassava cultivar Huanan 124.

For AGPase in leaves, the activity in F01 was about five times that in H124 at ‘seedling’ stage (Fig. 2a). For AGPase in stems, the activity in F01 was about 1.8 and about 1.7 times that in H124 at the ‘seedling’ and formation of root system stages (Fig. 2b), respectively. For AGPase in roots, the activity in F01 was about twice that in H124 at formation of root system, and about 1.4 times that in H_124_ at root bulking (Fig. 2c).

With respect to starch synthase, the activity in roots of F01 was 1.4 times that in roots of H124 at root bulking but significantly (p < 0.05) lower than that in roots of H124 at starch maturity (Fig. 2d).

Notably, the amylase activity in roots of H124 was 1.2 times that in roots of F01 at formation of root system (Fig. 2e). It should be pointed out that the activity of this enzyme was obviously lower in roots of F01 than in roots of H124 at starch maturity although the difference was not up to a significant level at p < 0.05 (Fig. 2e).

### Sugar content in the tissues

Sucrose, glucose and fructose are three mutually related sugars that are all associated with starch biosynthesis^23^.

**Figure 3:**
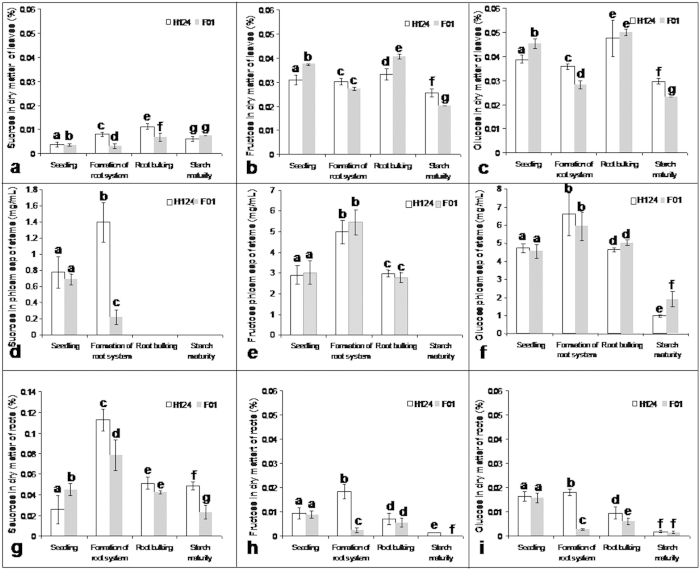
Sucrose (a), fructose (b) and glucose(c) in the dry matter of cassava leaves; sucrose (d), fructose(e) and glucose (f) in the sap of cassava stem phloem; and sucrose (g), fructose (h) and glucose (i) in the dry matter of cassava roots. Plants of the same size were chosen. Leaf tissue samples (not including petioles) from the sixth leaf down from the top of each plant were used. For assay of sugars in the sap of stem phloem, stems within 10–20 cm above the ground were ring-cut at 10 a.m. with a scalpel, and the phloem sap flowing from the wound was collected with a pre-chilled plastic syringe of a syringe needle. The roots were fibrous roots at the first two growth stages of ‘seedling’ and formation of root system, and the storage roots at the other two growth stages of root bulking and starch maturity. For analysis, root tissues were taken from whole fibrous roots or from the middle sections of the storage roots. Each datum was the mean ± SD from three individual plants. Different letters on the columns indicate the statistical difference at *p* < 0.05. F01, cassava cultivar Fuxuan 01. H124, cassava cultivar Huanan 124.

For sugars in leaves, the sucrose content was significantly (p < 0.05) lower in F01 than in H124 at the two stages of formation of root system as well as root bulking (Fig. 3a). The fructose content was much higher in F01 than in H124 at the two stages of ‘seedling’ and root bulking, but significantly (p < 0.05) lower in F01 than in H124 at starch maturity (Fig. 3b). The glucose content was significantly (p < 0.05) higher in F01 than in H124 at the ‘seedling’ stage, but significantly (p < 0.05) lower in F01 than in H124 at the two stages of formation of root system as well as starch maturity (Fig. 3c).

For sugars in the phloem sap of stems, the sucrose content was significantly (p < 0.05) lower in F01 than in H124 at formation of root system; however, it was scarcely detected in both cassava cultivars at the two stages of root bulking and starch maturity (Fig. 3d). The fructose content showed no significant (p < 0.05) difference between F01 and H124 at the first three stages; however, it was scarcely detected in both cassava cultivars at starch maturity (Fig. 3e). The glucose content was significantly (p < 0.05) higher in F01 than in H124 only at starch maturity (Fig. 3f).

With respect to sugars in roots, the sucrose content was significantly (p < 0.05) higher in roots of F01 than in roots of H124 at the ‘seedling’ stage. However, the content of this sugar was significantly (p < 0.05) lower in roots of F01 than in roots of H124 both at formation of root system and at starch maturity (Fig. 3g). The fructose content was significantly (p < 0.05) lower in roots of F01 than in roots of H124 both at formation of root system and at starch maturity (Fig. 3h). The glucose content was significantly (p < 0.05) lower in roots of F01 than in roots of H124 both at formation of root system and at root bulking (Fig. 3i).

### SFR in a 24-h day/night cycle

**Figure 4:**
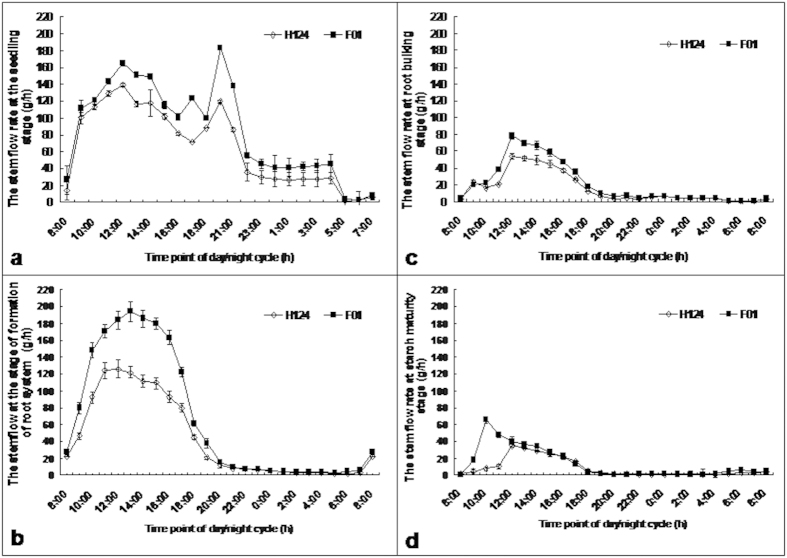
Diurnal changes of F01 and H124 in SFR at the stages of ‘seedling’ (a), formation of root system (b), root bulking (c) and starch maturity (d). Plants of the same size were chosen for assay of the SFR. The SFR assay was conducted on the stem 10 cm above the ground in a daily cycle of 24 h during sunny days. Each datum on the columns was the mean ± SD from three individual plants. F01, cassava cultivar Fuxuan 01. H124, cassava cultivar Huanan 124. SFR, stem flow rate.

Given that the stem flow is closely associated with matter transport between roots and shoots, we monitored and compared the SFRs from stems of F01 and H124. It was found that, during the day, the SFR was always higher in stems of F01 than in stems of H124 (Fig. 4). For both cassava cultivars, the SFR showed two peaks about at 12:00 and 20:00, respectively, at the ‘seedling’ stage (Fig. 4a). It peaked once between 12:00 and 13:00 at formation of root system (Fig. 4b) and at 12:30 at root bulking (Fig. 4c), respectively. However, the peak of the SFR appeared at approximately 10:00 for F01 and at 12:00 for H124 at the maturity stage (Fig. 4d), respectively. The SFR was much higher during the day than at night, and became very low at night after the ‘seedling’ stage (Fig. 4 b-d).

### Xylem vascular bundles of leaf axils, and phloem sieve plates (PSPs) of stems

**Figure 5:**
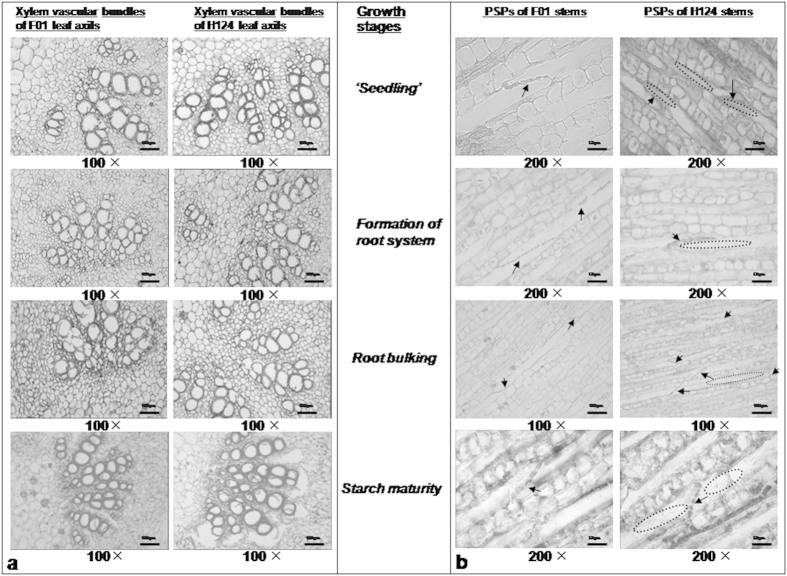
Xylem vascular bundles in the leaf axils (a); and PSPs of sieve tubes in the stem phloem of cassava (b). Plants of the same size were chosen. Leaf axil tissues from the junction between the stem and petiole of the sixth leaf down from the top of the plants were used for observation of xylem vascular bundles. Stem tissue samples from stems within 10–20 cm above the ground were used for observation of PSPs. Leaf axil tissues and stem tissues were sectioned transversely and longitudinally, respectively, by paraffin section. The thickness of the tissue slices was 10 μm. Three individual plants were examined for each cassava cultivar. The arrows indicate PSPs. The dotted circles indicate callose around the PSPs. F01, cassava cultivar Fuxuan 01. H124, cassava cultivar Huanan 124. PSPs, phloem sieve plates.

Because there was a considerable difference between F01 and H124 in SFR (Fig. 4), we examined the xylem vascular bundles in the leaf axil as well as the PSPs in stems. The former act as ‘bottlenecks’ that restrict fluid exchange between the leaf’s petioles and stems, and the latter are associated with transport of metabolites. By examining the xylem vascular bundles of leaf axils of the sixth leaves (counting down from the top of the plants) from three individual plants, we found that xylem vascular bundles in leaf axils of F01 were arranged compactly at the first three growth stages (Fig. 5a), obviously differing from the disperse and loose arrangement of those in the leaf axils of H124 (Fig. 5a).

The transport of the photosynthate from leaves to roots must pass the PSPs between elongated cells of the sieve tubes in the phloem^24^. We reasoned that the differences in SFR between F01 and H124 was likely to be associated partly with differences in PSPs. There was little or no callose around the PSPs of F01 stems, whereas a large amount of visible callose was deposited on both sides of the PSPs of H124 stems, which plugged the pores of PSPs and sometimes almost filled the space in the elongated cells of the sieve tubes (Fig. 5b).

### Expression profile of the genes concerned

Referring to our cassava cDNA database^19^, as well as the public cassava cDNAs (http://www.ncbi.nlm.nih.gov/genbank/), we chose a total of 106 genes and analysed their expression in roots and stems of F01 and H124 at different growth stages by using quantitative real-time polymerase chain reaction (qPCR). These genes were roughly categorised into antiporter/transporter, starch metabolism-related enzyme, signaling, sucrose synthase, growth and development, and vascular system (Supplementary Table S1). It should be pointed out that AGPase genes were not assayed because no appropriate sequence-specific primers were found to be available in qPCR. As expected, expression of the majority of the starch biosynthesis-related genes was up-regulated in roots of F01 when compared with that in roots of H124 at three growth stages except for formation of root system (Table 1). However, more than 90% of these genes were expressed in an up-regulation in stems of F01 when compared with that in stems of H124 at all four growth stages (Table 2).

## Discussion

Our results showed that F01 is a cassava cultivar with high root starch when compared with cassava cultivar H124 (Fig. 1a). Although starch accumulation in cassava roots may occurs early at 25–40 DAP^13^, little was known about the growth stages at which massive starch accumulation in roots occurs. The obvious increase in density of starch granules (Fig. 1d) together with starch content (Fig. 1a) clearly indicate that significant starch accumulation in roots of cassava may start to occur in the fibrous roots at the stage of formation of root system (Fig. 1d).

AGPase activity in roots of high-starch F01 was significantly (p < 0.05) higher than that in roots of low-starch H124 at two stages of formation of root system as well as root bulking (Fig. 2c). The starch synthase activity was obviously higher in roots of F01 than in roots of H124 at the two stages of formation of root system as well as at root bulking (Fig. 2d). The amylase activity was obviously lower in roots of F01 than in roots of H124 at two stages of formation of root system as well as starch maturity (Fig. 2e). Together, these results strongly suggest that greater starch synthesis capacity, and low starch degradation in roots are associated with high accumulation of starch in storage roots at late growth stages.

Although sucrose, glucose and fructose are all the substrates for starch biosynthesis, their contents in the different cassava tissues did not show either uniform or orderly changes with growth (Fig. 3) possibly because they could be dynamically interconverted during starch biosynthesis^23^,^25^. In leaves of both F01 and H124 at each growth stage the content of sucrose was much lower than that of either glucose or fructose (Fig. 3), signifying that the sucrose synthesised in leaves was likely exported in a timely manner and transported via stems into roots since the relatively higher content of sucrose occurred in stem phloem sap (Fig. 3d) and roots (Fig. 3g). No sucrose could be detected in the phloem sap of stems at the two stages of root bulking and starch maturity (Fig. 3d), whereas the content of both fructose (Fig. 3e) and glucose (Fig. 3f) was very high in the phloem sap of stems, respectively, at the corresponding stage(s), strongly suggesting that part of the sucrose was degraded into glucose and fructose during transport via stems. No fructose (Fig. 3e) but a relatively higher glucose content (Fig. 3f) in the phloem sap of stems at starch maturity signified that fructose was subsequently converted into glucose. Taking our results together with the literature^23^,^25^, it is clear that the complexity of conversion of sucrose, glucose and fructose in the tissues of cassava as growth occurs is far beyond our expectations.

Sucrose is a non-reducing sugar^26^, and it is the major form of sugar to be transported from leaves to roots^25^,^27^. Both glucose and fructose are reducing sugars^27^. A long-held contention is whether only non-reducing sugars could be transported over long distance in the phloem^26^,^28^. In two plant families (Ranunculaceae and Papaveraceae), translocation of reducing sugar species such as hexose can be regarded as a normal mode of carbohydrate transfer by the phloem^26^. The occurrence of glucose and fructose in the phloem sap of stems (Fig. 3e,f) indicated that reducing sugars could also be transported via the stem phloem in cassava. Certainly, we cannot preclude the possibility that a proportion of these two reducing sugars existing in the stem phloem has resulted from degradation of sucrose in the phloem.

When compared with the wild type, the tubers of transgenic potato over-expressing a sucrose non-fermenting-1-related protein kinase-1 gene under the control of a tuber-specific promoter showed a decrease in glucose levels by 17–56%, but it presented an increase in starch content by 23–30%^29^. Coincidentally, the glucose content in roots of high-starch F01 was significantly (p < 0.05) lower than that in roots of low-starch H124 at the two stages of formation of root system as well as root bulking (Fig. 3i), suggesting that there are potential differences between the cassava cultivars in partitioning of the sugar resource in the starch synthesis pathway.

Transport of water and nutrients inside plants usually depends on the vascular system composed of xylem and phloem, which are responsible for root-to-shoot transport and for leaf-to-root transport, respectively^24^. The water flow in the xylem is re-circularised from phloem to xylem and provides the hydraulic pressure required by transport in the phloem^27^. Therefore, it is reasonable to believe that the high SFR can bring about a greater transport ability to transport sugars from leave to roots, consequently allowing roots to obtain, in a timely manner, the sugars necessary for starch synthesis. More significantly, the timely supply of the sugars can inhibit the starch degradation^30^. The highest SFR for the tow cassava cultivars appeared both at the stages of ‘seedling’ and at formation of root system (Fig. 4). This is because these two growth stages take place at midsummer of the high temperature. However, the SFRs were always much higher in stems of high-starch F01 than in stems of low-starch H124 during the day (Fig. 4), strongly indicating that there is a more powerful stem transport in high-starch cultivars than in low-starch cultivars. This may explain why the sucrose content was much lower in the stem phloem sap of F01 than in that of H124 at formation of root system (Fig. 3d). Another potential benefit from high SFRs is likely to be a reduction in the water content of the storage roots, which would indirectly increase their dry matter content. We noted that the sugars were, on the whole, lower in roots of high-starch F01 than in those of low-starch H124 after the stage of formation of root system (Fig. 3g–i). This, in conjunction with the greater activity of AGPase (Fig. 2c) and starch synthase (Fig. 2d) in the roots of high-starch F01 at the two growth stages of root system formation as well as at root bulking, indicates that sugars are used more efficiently in starch biosynthesis in roots of high-starch F01 than in roots of low-starch H124.

In plants, most of the resistance to transport is considered to be axial rather than radial (via membrane transport), which results from the sieve plates^31^. Xylem transport in plants, depends, to a great extent, on the structure of the xylem pipeline system^32^. Undoubtedly, the more compact arrangement of the xylem bundles in the leaf axils of F01 (Fig. 5a) is favorable to formation of high SFR (Fig. 4) because such an arrangement may help to enhance the efficiency of fluid exchange between the leaves and stems per unit area per unit time. More phloem callose could lead to a concomitant reduction in lateral movement of ^14^C-assimilates^33^. Recently, it was suggested that in *Arabidopsis* the callose lining of sieve plate pores of the phloem confers favorable flow characteristics on the pores possibly because it can prevent collapse of the walls of the sieve plate pores^34^. However, more callose around PSPs could, after all, result in a higher resistance to water and nutrient transport via a stem^34^. Therefore, the reason for the high SFR in stems of F01 should be partially attributed to relatively smooth transportation in the phloem pipeline system because of the limited amount of callose in the sieve tubes (Fig. 5b).

The higher SFR and/or the compact arrangement of xylem bundles in F01 seemed to be associated with the high expression of genes related to the vascular system, cytoskeleton and cell expansion in stems and/or roots of F01 (Tables 1 and 2). Of these genes, high expression of late embryogenesis abundant protein genes (FG805555 and FG807051) might be helpful in increasing the SFR because the related proteins usually serve as ‘space fillers’ to prevent cellular collapse and consequently to ensure the stability of molecular interactions via protecting cytoskeleton^35^. The genes involved in the development of the vascular tissue were highly expressed in F01, and were found to be those encoding the GRP-like protein 2^36^ and S-adenosyl methionine synthases (FG806323, FG806486 and FG806831)^37^,^38^,^39^. Of interest was tubby-like protein (FG806408) and expansin-like protein precursor (FG807273) genes. The tubby-like protein participates in the ABA signaling pathway in *Arabidopsis*^40^ and causes the tubby syndrome in the mouse^41^. It is worth noting that the tubby-like protein gene (FG806408) showed temporarily down-regulated expression in roots of F01 when compared with that in roots of H124 at formation of root system (Table 1). This indicates that the regulation of the root systems probably occurs because that stage is decisive in differentiation of adventitious roots, fibrous roots and storage roots in cassava^20^. The expansin-like protein precursor gene (FG807273) was expressed in an up-regulation in roots of F01 relative to that in roots of H124 at starch maturity. The expansin protein is one of the key regulators of cell wall extension during growth^42^. This strongly implies that the volume of root cells in F01 is probably larger than that in H124. The larger cell volume is, of course, favourable to accumulation of more starch in roots. Taken together, this may well imply that the high-starch cassava cultivar F01 is relatively more flexible in its cell expansion than is H124.

The ADP/ATP antiport system supplies plastids with ATP as the driving force for biosynthetic processes^43^. It was found in maize that the mutation in the ADP/ATP antiport system resulted in a reduction in starch biosynthesis^43^. Therefore, the high-level expression of ADP/ATP antiporter genes in roots of F01 (Tables 1) would be more conducive to starch synthesis. The genes encoding glucose transporter (FG805259) and hexose carrier protein (FG806102) were expressed in an up-regulation in stems and/or roots of F01 (Tables 1 and 2), implying that stems of this cultivar have relatively higher loading and unloading efficiencies of the sugars because the related proteins are required for efficient transportation of sugars via phloem-xylem systems^44^,^45^.

Sucrose is destined either to be degraded by invertase to glucose and fructose before entering the cytosol or to be converted by sucrose synthase to UDP-glucose after entering the cytosol in cassava roots^11^. The resulting UDP-glucose is also reversely converted by sucrose synthase into sucrose in the cytosol^11^. Interestingly, one sucrose synthase gene (AY818397) was always expressed in a down-regulation in roots of F01 at three growth stages except at ‘seedling’ when compared with that in roots of H124 (Table 1). In contrast, this sucrose synthase gene was expressed in an extreme up-regulation in stems of F01 (Table 2). Therefore, it is likely that the presence of sucrose in the form of UDP-glucose facilitates loading and unloading in the stem phloem during long-distance transport. After being transported to destinations such as roots, and under low conversion of sucrose to UDP-glucose by sucrose synthase in roots, sucrose predominance among the sugars there (Fig. 3g–i) probably promoted a positive reaction from sucrose to glucose-6-P that is generally used for starch biosynthesis in cassava roots, indirectly supporting a previous proposal^11^. It can be assumed, therefore, that low expression of the sucrose synthase gene in roots is an important factor resulting in high starch accumulation in cassava storage roots.

The high expression of the hexokinase gene (FG805239) in roots (Table 1) and stems (Table 2) of F01 implies that F01 has a stronger ability to use the hexoses to synthesize starch. This is because the encoded hexokinases can catalsze such hexoses as glucose and fructose to form hexose monophosphates^46^,^47^, which constitute a so-called Hx-P pool for starch biosynthesis^11^.

However, the total amylase activity is significantly lower in roots of F01 than in those of H124 at the two stages of formation of root system as well as starch maturity (Fig. 2e), but the expression level of the two amylase-related genes assayed (DQ017830 and AY944584) was higher in roots of F01 than in those of H124 at the two stages of root bulking and starch maturity (Table 1). Such incompatibility between the enzyme activity and expression pattern of the amylase-related genes is likely because the amylase is an enzyme system that is usually composed of different isoforms^48^. Actually, it has been found in rice that isoforms of amylase are encoded by different genes and showed differential expression in germinating rice^49^. In contrast, the amylase isoforms in barley have usually been found to be subjected to posttranslational modification^50^.

**Figure 6:**
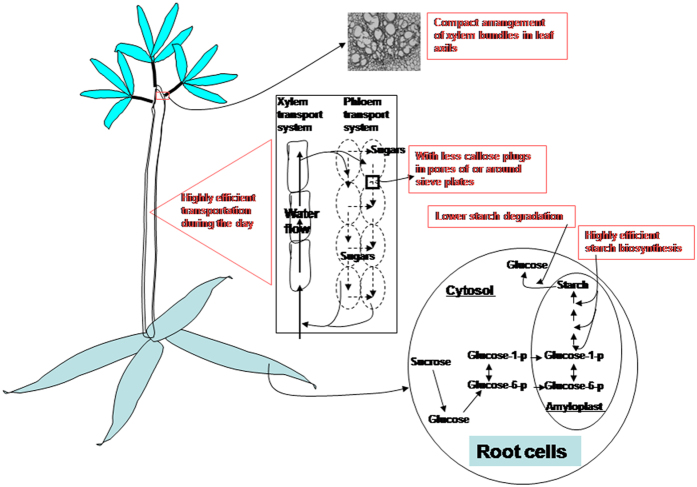
Schematic characters related to high root starch accumulation in cassava storage roots. This model emphasises that high starch accumulation in the cassava roots is associated with the capacities of stronger stem transport supported by high SFR; higher loading and unloading efficiencies of the sugars through high expression of genes encoding glucose transporters and hexose carrier proteins in the phloemxylem systems of the stems; high starch synthesis efficiency dependent on high AGPase and starch synthase activities, low expression of the sucrose synthase genes and high-level expression of ADP/ATP antiporter genes in storage roots; lower starch degradation due to low amylase activity and the timely supply of the sugars in roots; and high starch storage capacity which probably results from the increased cell volume due to high expression of expansin protein genes in storage roots. Stronger stem transport is associated with the more compact arrangement of xylem vascular bundles in leaf axils, and much less callose in sieve tubes and around the sieve plates of the stem phloem. SFR, stem flow rate.

The above discussion makes it clear that the high starch accumulation in the cassava roots is a very complex process, and one that results from a combination of physiological, cellular, biochemical and gene expression. However, our results suggest that some characters related to high starch accumulation can be sketched out. Therefore, we propose, with caution, a model on formation and high accumulation of starch in cassava roots (Fig. 6). The model emphasises that high starch accumulation in roots of cassava is associated with the capacities of stronger stem transport supported by high SFR; higher loading and unloading efficiencies of the sugars through high expression of genes encoding glucose transporters and hexose carrier proteins in the phloem–xylem systems of the stems; high starch synthesis efficiency dependent on high AGPase and starch synthase activities, low expression of the sucrose synthase genes, and high-level expression of ADP/ATP antiporter genes in storage roots; lower starch degradation, not only from low amylase activity, but also benefiting from the timely supply of the sugars to the roots; and high starch storage capacity, which is likely to result from the increased cell volume owing to high expression of expansin protein genes in storage roots. The stronger stem transport is associated not only with the more compact arrangement of xylem vascular bundles in the leaf axils but also with much less callose in sieve tubes and around the sieve plates of the stem phloem.

## Methods

### Cassava cultivation and tissue sampling

The two cassava cultivars used were H124 and F01. The cassava plants started from stem stakes and were grown in the experimental field of the Agricultural College of Guangxi University in the 2010–2011 growing season following conventional cultivation and management methods. Sampling or assay was conducted at 10:00 a.m. on plants of the same size, which were from non-marginal effect areas in the experimental field at the four growth stages of ‘seedling’, formation of root system, root bulking, and starch maturity.

The sixth leaves, without petioles, counting down from the top of the plants, were used as leaf samples. The root surface was carefully cleaned of clay, and the root bark (periderm) was removed. The roots were then quickly rinsed with sterile water before tissue sampling. Root tissue samples were taken from whole fibrous roots at the first two growth stages of ‘seedling’ and formation of root system, or from the tissues in the middle sections of the storage roots at other two growth stages of root bulking and starch maturity. The storage roots were cross-sectioned for tissue sampling in a fan-shaped manner, where the arc length of fan-shaped tissues was 1/8 of the cross-sectional perimeter.

The stem samples were from parts of the stems within 10–20 cm above the ground, and the old bark of the stems was removed before use.

The leaf axil samples of the sixth leaves, counting down from the top of the plants were from tissues located at the junction between stems and petioles.

Aliquots of the sampled tissues were immediately frozen in liquid nitrogen and then stored at –80 °C for biochemical analyses, while another aliquots of the sampled tissues were immediately fixed for paraffin section in fixative comprising 90 mL of 70% ethanol, 5 mL glacial acetic acid and 5 mL 37% formaldehyde.

### Assay of starch content

The tissues were immersed for 12 h in anhydrous ethanol to remove the interfering coloured substance, dried for 12 h at 60 °C fully pulverised. The resulting tissue powder was then sieved through a 60-mesh sieve. A 20 mg aliquot of the tissue powder was added to 8 mL of 2 M KOH followed by heating for 15 min in a water bath at 70 °C. The mix was adjusted to pH 3.0 with 2 M HCl and then to 25 mL with sterile ultrapure water. A 4 mL aliquot of the powder–solution mix was combined with 1 mL 1% KI-0.1% I_2_ solution and allowed to react for 15 min at room temperature. The amylose content in the reaction solution was then estimated upon optical density (OD) value at 620 nm against the standard curve established upon OD_620nm_ with standard amylose (Sigma). The amylopectin content in the reaction solution was estimated upon OD_550nm_ value against the standard curve established upon OD_550nm_ with standard amylopectin (Sigma). The content of either amylase or amylopectin in the dry matter of the tissues was calculated as a formula: (%) = [(content (mg/L) of the reaction solution × 25 mL)/20 mg] × 100%. The total content of the starch in the dry matter of the tissues was the sum of amylose and amylopectin.

**Extraction of crude enzyme**. The crude extracts of AGPase or starch synthase were prepared following the previous methods^51^ with minor modifications. In brief, a 0.5 g aliquot of the tissues was fully homogenised on ice in a 1.5 mL pre-cooled solution composed of 100 mM Hepes-NaOH at pH 7.4, 8 mM MgCl_2_, 2 mM EDTA, 12.5% (v/v) glycerol, 5% (v/v) polyvinylpyrrolidone, and 50 mM β-mercaptoethanol. The resulting homogenate was centrifuged for 5 min at 4 °C at 10,000 × *g*. The supernatant was collected and stored at −20 °C as the crude extracts of AGPase or starch synthase.

The crude extract of the amylase was made as the previous methods^52^ with minor modifications. Briefly, a 0.5 g aliquot of the tissues was fully homogenised on ice in a pre-cooled 10 mL of 0.1 M citric acid at pH 5.6. The resulting homogenate was then immediately centrifuged for 15 min at 2,200 × *g* at 4 °C. The supernatant was collected and stored at −20 °C as the crude extract of the amylase.

### Collection of stem phloem sap

Stems within 10–20 cm above the ground were ring-cut at 10 a.m. with a scalpel. This was carried out with care to avoid reaching the pith tissues inside the stems. Once phloem sap was flowing from the wound it was collected quickly, with a pre-chilled plastic syringe of a syringe needle, because the discharged sap clots easily owing to water evaporation. The collected sap was immediately transferred into a tube that was placed into liquid nitrogen and subsequently stored at −80 °C for further analysis.

### Assay of activities of the enzymes

For AGPase activity, a 20 μL aliquot of the crude enzyme extract was mixed well with 110 μL reaction solution comprising 100 mM Hepes-NaOH at pH 7.4, 5 mM MgCI_2_, 3 mM pyrophosphoric acid, 4 mM dithiothreitol (DTT) and 1.2 mM adenosine diphosphate glucose (ADPG) and allowed to react for 20 min in a 30 °C water bath. The reaction was then terminated by a treatment for 30 s in a 100 °C water bath. The reaction solution was cooled rapidly in an ice-water bath, and then centrifuged for 10 min at 10,000 × *g* at 4 °C. A 100 μL aliquot of the supernatant was added to 5.2 μL solution composed of 5.76 mM nicotinamide adenine dinucleotide phosphate,(NADP) 0.07 U glucose-6-P-dehydrogenase and 0.08 U β-glucomutase, and allowed to react for 10 min in a 30 °C water bath. The reaction was terminated by treatment for 30 s in a 100 °C water bath. A control was conducted in parallel in the reaction system composed of a 20 μL aliquot of the crude enzyme extract and the sterile deionised water to correct the background glucose release. The OD_340 nm_ values in the reaction solution were measured and normalized by OD_340 nm_ values resulting from the control. The enzyme activity was then estimated with normalized OD_340 nm_ values in the reaction solution against the standard concentration curve established at 340 nm with a series of 100 mM Hepes-NaOH solutions with gradient concentrations of NADPH.

To examine starch synthase activity, a 20 μL aliquot of the enzyme extract was mixed with 36 μL solution composed of 50 mM Hepes-NaOH at pH 7.4, 15 mM DTT, 0.7 mg amylopectin and 1.6 mM ADPG, and allowed to react for 20 min. The reaction was terminated by treatment for 30 s in a 100 °C water bath, and then cooled rapidly in an ice-water bath. The cooled reaction solution was further mixed with a 20 μL solution containing 50 mM Hepes-NaOH at pH 7.4, 1.2 U pyruvate kinase, 4 mM phosphoenolpyruvate, 10 mM MgC1_2_ and 200 mM KC, and allowed to react for 20 min in a 30 °C water bath. The reaction was terminated by treatment for 30 s in a 100 °C water bath. The reaction solution was immediately cooled to 0 °C in an ice-water bath, and then centrifuged for 10 min at 10,000 × *g* at 4 °C. A 60 μL aliquot of the supernatant was mixed with 43 μL solution containing 50 mM Hepes-NaOH at pH 7.4, 2 mM NADP, 10 mM glucose, 0.35 U glucose-6-P dehydrogenase, 1.4 U hexokinase and 20 mM MgC1, and then allowed to react for 10 min in a 30 °C water bath. The reaction was terminated by treatment for 30 s in a100 °C water bath. The starch synthase activity was then determined with the OD_340 nm_ values in the reaction solution against a standard concentration curve established with gradient concentrations of NADPH at 340 nm.

To examine amylase activity, a 1 mL aliquot of the crude enzyme extract was dispensed equally into 48 tubes. These tubes with the enzyme extracts were then divided into two groups, 24 tubes each. A 1 mL aliquot of 1% soluble starch (Sigma) was added to each tube in one group as treatment, while a 1 mL aliquot of sterile ultrapure water was added to each tube in another group as control. All 48 tubes were placed for 5 min in a 40 °C water bath, and a 3 mL aliquot of the solution composed of 1 g 3,5-dinitrosalicylic acid, 20 mL of 2 M NaOH and 30 g Seignette salt was then added to each tube. Subsequently, the solution volume of each tube was adjusted to 100 mL with sterile ultrapure water. The tubes were allowed to react for 10 min in a 30 °C water bath and for 10 min in a 100 °C water bath to terminate the reaction, and then immediately cooled to 0 °C in an ice-water bath. The OD_540nm_ values from all the assays were normalised with OD_540nm_ values from the respective controls conducted in the solutions without the crude enzyme extract. The amylase activity was then estimated with the OD_540nm_ values in the normalised reaction solution against a standard concentration curve established at 540 nm with reactions between 3,5-dinitro salicylic acid and a series of concentration gradients of maltose.

### Quantitation of sugars in the tissues, and stem phloem sap

The sugars in the tissues and phloem sap of stems were assayed as the methods found in the literature^53^,^54^ with some modifications.

The tissues were first baked for 10 min at 110 °C to inactivate endogenous sugar-digesting enzymes, and then kept at 80 °C until dried. The dried tissues were ground into powder and sieved though a 60 mesh sieve. A 0.1 g aliquot of the sieved powder was added to 10 mL of 80% ethanol and then positioned for extraction for 30 min in a 80 °C water bath. A 2 mL aliquot of the resulting extract was centrifuged for 10 min at 12,851 × *g* at 4 °C. A 1.5 mL aliquot of the supernatant was concentrated in an Eppendorf^®^ tube plate for concentrator 5301 (Eppendorf, Germany). The resulting concentrate was dissolved in 1.5 mL sterile ultrapure water followed by centrifugation for 10 min at 12,851 × *g*. The supernatant was filtered by using a Acrodisc Syringe Filter (PN:4614, PALL). The filtrate was then used for assay of sugars by using the high-performance liquid chromatograph (HPLC) system equipped with a CBM-10A vp Plus instrument (Shimadzu) as a RID-10A detector model, where the column used was a Aminex HPX-87P column (Bio-Rad, Germany). The sample loaded in the column was 20 μL. The HPLC was operated at a flow-rate of 0.6 mL/min at a column temperature of 80 °C. The used standard sugars were purchased from Sangon Biotech (Shanghai, China).

For sugars in phloem sap of stems, a 15 μL aliquot of the phloem sap of stems was diluted to 1.5 mL with 80% ethanol, and then centrifuged for 10 min at 10,621 × *g* at 4 °C. The resulting supernatant was concentrated in the vacuum concentrator. The concentrate was dissolved with 1.5 mL sterilised ultrapure water and then filtered by using the Acrodisc Syringe Filter. The resulting filtrate was used for assay of sugars by HPLC as indicated above.

### Assay of SFR

We conducted the SFR assay on the stem within 10 cm above the ground in a daily cycle of 24 h during sunny days with the Flow 32-1K™ Sap Flow System (Dynamax Inc., USA) equipped with the Dynagage sensor, following the default parameters of the program F32-1K_30 min.cr1 provided by the memory device CR1000 of the Sap Flow System. In brief, the operating parameters for the sensor were SGB16 for DG_Type(1), 99.6 for DG_HR(1), 5 for DG_dx(1), 0.54 for DG_Kst(1), 2.2686 for DG_SA(1), 2.0 for DG_IA(1), 0.5 for DG_LCutT(1), 152 for DG_HCutV(1), and 0.8 for D G_Ksh(1).

### Observations of starch granules, xylem vascular bundles and PSPs

The fixative-fixed tissue samples of stems, roots and leaf axils, about 0.2 cm^3^, were examined and sectioned through paraffin section by using a Leica RM2235 manual rotary microtome (Leica, Germany) following the protocols established by our laboratory. The leaf and leaf axils tissues were transversely sectioned, and the stem tissues were longitudinally sectioned. The tissues were sliced to a thickness of 10 μm. For observation of starch granules in cells, the tissue slices were stained in a conventional I_2_ (2%)-KI(1%) solution. Photographs were taken with a BX41 optical microscope (Olympus, Japan) equipped with a Qimaging MicroPublisher 5.0 RTV image collector.

### qPCR

The total RNA of each sample was extracted from the mix of equal amounts of tissues from three individual plants by using the cetyltrimethylammonium bromide containing 4% β-mercaptoethanol. The RNA integrity was examined through agarose gel electrophoresis, and the RNA purity was estimated by measuring OD_260nm_/OD_280nm_ and OD_260nm_/OD_230nm_ ratios. The first strand cDNA was synthesised with the extracted RNA as a template by using the All-in-One™ First-Strand cDNA Synthesis Kit (GeneCopoeia, China) following the kit’s instructions. The subsequent quantitative PCR was carried out on the iQ5 Real Time PCR Detection System (Bio-Rad, USA) by using the All-in-One^TM^ qPCR Mix (GeneCopoeia, China). The detailed PCR conditions in PCR are presented in Supplementary Table S2.

For each gene, at least four pairs of sequence-specific primers were designed. The specificity of the primers was examined through homology analysis of the expressed sequence tags of cassava against the public database. The amplification efficiency of all these primers was pre-tested and only one pair of the primers, with the highest amplification efficiency, was used for qPCR (Supplementary Table S2). The optimum amplicon concentration was determined with the serial dilutions of the first strand cDNA synthesised before PCR. The expression analysis for each gene was technically repeated three times. The detailed amplification cycles of the genes in qPCR are listed in Supplementary Tables S3, S4, S5, S6, S7, S8, S9 and S10.

The cassava actin gene was used as an internal control in qPCR to normalise the gene expression. The controls were conducted in parallel in reactions with a PCR mix reagent but without a cDNA template to monitor contamination of the reaction systems. The relative expression level of the gene in F01 tissues was determined as a comparison of F01 roots (or stems) vs. H124 roots (or stems) at the same growth stages, and calculated as 2^−ΔΔCt(ΔCt^_F01_^−ΔCt^_H124_). Differential expression was defined according to a cutoff fold of ≥1.2 or ≤−1.2 at *p* < 0.01.

### Statistical analysis

Statistically significant difference was analysed by a *t*-test at p < 0.05 using SPSS17.0 version statistics software (SPSS Inc., USA).

## Additional Information

**How to cite this article**: Li, Y.-Z. *et al.* Characters related to higher starch accumulation in cassava storage roots. *Sci. Rep.* **6**, 19823; doi: 10.1038/srep19823 (2016).

## Supplementary Material

Supplementary Information

## Figures and Tables

**Table 1:** Differential expression of the selected genes in roots of cassava cultivar F01.

GenBank accession no. of gene	Description	*P* value inT test	Fold change in expression in F01 roots
**F01 vs. H124 at ‘seedling’**			
**Antiporter/Transporter**			
FG805259	Glucose transporter type 12	0.003	***1.5***
FG804711	ATP:ADP antiporter	0.004	***1.7***
**Starch metabolism related enzyme**			
DQ017830	*Manihot esculenta* alpha-amylase	0.005	***2.3***
**Signaling**			
FG806408	AtTLP3 (TUBBY LIKE PROTEIN 3)	0.000	***390.2***
FG805296	Pyruvate kinase, cytosolic isozyme	0.002	***1.8***
FG806368	pyrophosphate-dependent phosphofructo-1-kinas	0.000	***2.1***
**F01 vs. H124 at formation of root system**			
**Transporter**			
FG804711	ATP:ADP antiporter	0.000	−2.18
FG805197	Potassium/chloride transporters	0.001	−1.92
FG805211	Organic cation transporter	0.000	−1.41
FG805259	Glucose transporter type 12	0.000	−2.10
FG806078	Glucose-6-phosphate/phosphate-translocator	0.000	−5.01
FG805678	Chloroplast glucose-6-phosphate/phosphate translocator	0.004	−1.49
**Starch metabolism-related enzyme**			
DQ011041	*M*. *esculenta* alpha-amylase 2 (amy2)	0.000	−2.93
X74160	*M*. *esculenta* granule-bound starch synthase	0.000	−1.93
X74160	*M*. *esculenta* granule-bound starch synthase	0.000	−1.93
**Sucrose synthase**			
BM260279	Sucrose synthase	0.000	***1.35***
AY818397	*M*. *esculenta* sucrose synthase	0.000	−9.10
**Signaling**			
FG805239	Hexokinase	0.000	−2.98
FG806408	AtTLP3 (Tubby like protein 3)	0.008	−1.76
FG805296	Pyruvate kinase, cytosolic isozyme	0.000	−1.90
FG806368	Pyrophosphate-dependent phosphofructo-1-kinas	0.004	−1.60
**Growth and development**			
FG806385	Auxin response factor-like protein	0.001	−1.66
FG806743	Secretory carrier membrane protein (SCAMP) family protein	0.000	***2.58***
FG806552	Auxin-responsive factor TIR1-like protein	0.004	−1.25
FG807559	Putative ripening-related protein	0.002	−1.25
FG805555	Late embryogenesis abundant protein 5	0.000	−3.13
FG807051	Late embryogenesis abundant protein Lea5	0.000	−3.81
FG806890	Induced stolon tip protein	0.000	−2.44
FG806889	Cell division ATP-binding protein FtsE	0.005	−1.84
**F01 vs. H124 at root bulking**			
**Antiporter/Transporter**			
FG804711	ATP:ADP antiporter	0.008	−1.22
FG805321	Inner-membrane translocator	0.000	***2.84***
FG805211	Similar to Solute carrier family 22 (organic cation transporter)	0.001	***1.41***
**Vascular system**			
FG806684	GRP-like protein 2	0.000	***1.37***
FG807243	Fructose-bisphosphate aldolase, putative	0.001	***2.69***
**Starch metabolism related enzyme**			
DQ017830	*M. esculenta* alpha-amylase	0.001	*1.80*
X77012	*M. esculenta* starch branching enzyme	0.001	*2.24*
X69714	*M. esculenta* strach branching enzyme (r-3)	0.001	*1.20*
X74160	*M. esculenta* granule-bound starch synthase	0.000	−4.51
**Sucrose synthase**			
BM260279	Sucrose synthase	0.002	*1.35*
DQ443534	*M. esculenta* sucrose synthase	0.004	*1.28*
AY818397	*M. esculenta* sucrose synthase	0.000	−4.14
**Signaling**			
FG805239	Hexokinase	0.002	*1.48*
FG805296	Pyruvate kinase, cytosolic isozyme	0.007	*1.31*
FG806368	Pyrophosphate-dependent phosphofructo-1-kinas	0.000	*4.27*
**F01 vs. H124 at starch maturity**			
**Antiporter/Transporter**			
FG806078	Glucose-6-phosphate/phosphate-translocator	0.000	*1.69*
FG805211	Similar to Solute carrier family 22	0.005	*1.34*
FG805259	Glucose transporter type 12	0.000	*1.67*
**Vascular system**			
FG806684	GRP-like protein 2	0.003	*1.37*
**Starch metabolism related enzyme**			
DQ017830	*M. esculenta* alpha-amylase	0.000	*3.21*
AY944584	*M. esculenta* beta-amylase (AmyB)	0.004	*2.51*
X74160	*M. esculenta* granule-bound starch synthase	0.000	−3.02
EF667960	*M. esculenta* starch synthase isoform I (SSI)	0.006	−1.37
**Sucrose synthase**			
AY818397	*M. esculenta* sucrose synthase	0.005	−4.28
DQ443534	*M. esculenta* sucrose synthase	0.005	*1.40*
BM260279	Sucrose synthase	0.000	*1.74*
DQ443534	Sucrose synthase	0.005	*1.40*
**Signaling**			
FG805239	Hexokinase	0.001	*1.35*
FG805296	Pyruvate kinase, cytosolic isozyme	0.006	*1.35*
**Growth and development**			
FG805555	Late embryogenesis abundant protein 5	0.000	*2.00*
FG804941	Growth-on protein GRO10	0.005	*1.21*
FG807273	Expansin-like protein precursor	0.000	*2.15*
FG807559	Putative ripening-related protein	0.001	−1.45
FG807051	Late embryogenesis abundant protein Lea5	0.000	−5.44
DN740362.1	Similar to ethylene response factor	0.003	*1.50*
FG807216	Ethylene response factor	0.004	*1.22*

The plants were propagated by stem stakes and grown in the experimental fields of Agricultural College of Guangxi University in the 2010–2011 growing season following conventional cultivation and management. The expression of each gene in F01 and H124 was analyzed by qRT-PCR with a pair of cDNA sequence-specific primers, respectively. The figures in column ‘Fold change’ indicate a differential expression levels of the target genes in F01 roots under a comparison as F01 roots vs. H124 roots at the same growth stage, which was calculated following the formula of 2^−ΔΔCt(ΔCt^_F01 roots_^−ΔCt^_H124 roots_. The bold italic figures and negative figures in column ‘Fold change’ indicate the up-regulated and down-regulated expression levels of the genes, respectively, The up-regulated and down-regulated expression was determined according to a cutoff fold of ≧1.2 or ≦−1.2 at *p* < 0.01, respectively.

**Table 2:** Differential expression of the selected genes in stems of cassava cultivar F01.

GenBank accession no. of gene	Description	*P* value inT test	Fold change in expression in F01 stems
**F01 vs. H124 at ‘seedling’**			
**Antiporter/transporter**			
FG806078	Glucose-6-phosphate/phosphate-translocator	0.000	***4.29***
FG804711	ATP:ADP antiporter	0.001	***1.73***
FG805321	Inner-membrane translocator	0.000	***2.63***
FG805197	Potassium/chloride transporters	0.001	−1.70
FG805259	Glucose transporter type 12	0.001	***2.00***
FG806102	Hexose carrier protein	0.008	***2.30***
**Vascular system**			
FG806684	GRP-like protein 2	0.000	***3.14***
FG806176	Vacuolar protein sorting-associated protein	0.000	***1.72***
FG806269	Vacuolar sorting receptor protein	0.000	***1.49***
FG806323	S-adenosyl-L-methionine synthetase 1	0.001	***2.61***
FG806831	S-adenosyl methionine synthase-like	0.000	***1.97***
**Starch metabolism related enzyme**			
DQ011041	*M. esculenta* alpha-amylase 2 (amy2)	0.000	***1.67***
AY944584	*M. esculenta* beta-amylase (AmyB)	0.002	−2.66
X69714	*M. esculenta* branching enzyme (r-3).	0.001	−1.91
X74160	*M. esculenta* granule-bound starch synthase	0.000	***2310.19***
EF667961	*M. esculenta* starch synthase isoform II (SSII)	0.000	−1.71
EF667960	*M. esculenta* starch synthase isoform I (SSI)	0.002	−1.81
**Sucrose synthase**			
BM260279	Sucrose synthase	0.000	−1.42
AY818397	*M. esculenta* sucrose synthase	0.000	***408.29***
**Signaling**			
FG805239	Hexokinase	0.000	***3.31***
FG806368	Pyrophosphate-dependent phosphofructo-1-kinas	0.000	***8.03***
**Growth and development**			
FG807216	Ethylene response factor	0.001	***2.39***
FG806385	Auxin response factor-like protein	0.000	***1.87***
FG805555	Late embryogenesis abundant protein 5	0.000	***4.66***
FG807051	Late embryogenesis abundant protein Lea5	0.000	***18.14***
FG806890	Iinduced stolon tip protein	0.001	***2.65***
FG807273	Expansin-like protein precursor	0.000	***3.79***
**F01 vs. H124 at formation of root system**			
**Antiporter/Transporter**			
FG806078	Glucose-6-phosphate/phosphate-translocator	0.004	−1.59
FG806351	Golgi transport complex protein-related	0.000	***1.93***
FG805678	Chloroplast glucose-6-phosphate/phosphate translocator	0.000	***3.41***
FG805321	Inner-membrane translocator	0.004	***1.62***
FG805197	Potassium/chloride transporters	0.000	***4.05***
FG805211	Organic cation transporter	0.000	***2.69***
FG805259	Glucose transporter type 12	0.000	***4.12***
FG806102	Hexose carrier protein	0.000	***8.45***
**Vascular system**			
FG806176	Vacuolar protein sorting-associated protein	0.000	***5.11***
FG806269	Vacuolar sorting receptor protein	0.001	***2.26***
FG807089	Secondary cell wall-related glycosyltransferase family 8	0.000	***4.62***
FG806486	S-adenosylmethionine:2-demethylmenaquinone methyltransferase-like	0.000	**1.95**
**Starch metabolism related enzyme**			
DQ011041	*M. esculenta* alpha-amylase 2 (amy2)	0.000	***18.04***
DQ017830	*M. esculenta* alpha-amylase gene	0.000	***27.33***
X69714	*M. esculenta* branching enzyme (r-3).	0.000	***1.91***
X74160	*M. esculenta* granule-bound starch synthase	0.000	***3.36***
AF173900	*M. esculenta* granule bound starch synthase II precursor (GBSSII)	0.000	***3.97***
EF667961	*M. esculenta* starch synthase isoform II(SSII)	0.000	***3.36***
EF667960	*M. esculenta* starch synthase isoform I (SSI)	0.000	***2.39***
GU229751.1	Isoamylase (Meisa1) mRNA	0.005	***4.39***
**Sucrose synthase**			
BM260279	Sucrose synthase	0.000	***1.77***
**Signaling**			
FG805239	Hexokinase	0.000	***2.37***
FG806408	AtTLP3 (Tubby like protein 3)	0.000	***2.60***
FG806368	Pyrophosphate-dependent phosphofructo-1-kinas	0.000	***3.55***
**Growth and development**			
FG806385	Auxin response factor-like protein	0.000	***4.40***
FG806552	Auxin-responsive factor TIR1-like protein	0.000	***4.59***
DN740362.1	Similar to ethylene response factor	0.000	***2.58***
FG807559	Putative ripening-related protein	0.001	***2.62***
FG806890	Induced stolon tip protein	0.000	**2.08**
FG807273	Expansin-like protein precursor	0.000	**3.98**
FG805555	Late embryogenesis abundant protein 5	0.000	***4.53***
FG807051	Late embryogenesis abundant protein Lea5	0.000	***6.64***
FG804941	Growth-on protein GRO10	0.000	***3.68***
FG806004	Expansin-like protein precursor	0.001	***3.11***
**F01 vs. H124 at root bulking**			
**Antiporter/Transporter**			
FG806078	Glucose-6-phosphate/phosphate-translocator	0.000	***3.03***
FG806269	Vacuolar sorting receptor protein	0.007	***1.07***
FG805678	Chloroplast glucose-6-phosphate/phosphate translocator	0.002	***1.28***
FG805321	Inner-membrane translocator	0.002	−1.21
FG805197	Potassium/chloride transporters	0.000	***1.88***
FG805259	Glucose transporter type 12	0.000	***1.56***
FG806102	Hexose carrier protein	0.000	***4.24***
**Vascular system**			
FG806831	S-adenosyl methionine synthase-like	0.000	***1.78***
**Starch metabolism related enzyme**			
DQ011041	*M. esculenta* alpha-amylase 2	0.000	***2.04***
DQ017830	*M. esculenta* alpha-amylase	0.003	***5.16***
X77012	*M. esculenta* SBE starch branching enzyme	0.002	***1.79***
X74160	*M. esculenta* granule-bound starch synthase	0.000	***4.68***
EF667960	*M. esculenta* starch synthase isoform I (SSI)	0.006	***1.30***
X74160	*M. esculenta* granule-bound starch synthase	0.000	***4.68***
GQ227726.1	Isoamylase isoform	0.004	***2.05***
**Sucrose synthase**			
AY818397	*M. esculenta* sucrose synthase	0.000	***4.08***
**Signaling**			
FG805239	Hexokinase	0.006	***1.50***
FG806368	Pyrophosphate-dependent phosphofructo-1-kinas	0.000	***3.79***
**Growth and development**			
FG807216	Ethylene response factor	0.003	***1.17***
FG806385	Auxin response factor-like protein	0.000	***1.50***
FG807559	Putative ripening-related protein	0.000	***2.24***
FG805555	Late embryogenesis abundant protein 5	0.000	***2.95***
FG807051	Late embryogenesis abundant protein Lea5	0.000	***4.83***
FG806004	Expansin-like protein precursor	0.001	***3.12***
**F01 vs. H124 at starch maturity**			
**Transporter**			
FG806078	Glucose-6-phosphate/phosphate-translocator	0.000	***4.62***
FG804711	ATP:ADP antiporter	0.002	***2.07***
FG805321	Inner-membrane translocator	0.001	−1.46
FG805197	Potassium/chloride transporters	0.006	***1.53***
FG805259	Glucose transporter type 12	0.002	***1.35***
FG806102	Hexose carrier protein	0.000	***3.29***
**Amylase**			
DQ011041	*M. esculenta* alpha-amylase 2	0.000	***3.23***
DQ017830	*M. esculenta* alpha-amylase	0.000	***6.92***
GU229751.1	Isoamylase (Meisa1)	0.000	***2.21***
**Starch synthase**			
X74160	*M. esculenta* granule-bound starch synthase	0.000	***3.45***
EF667961	*M. esculenta* starch synthase isoform II (SSII)	0.005	***1.65***
X74160	*M. esculenta* granule-bound starch synthase	0.000	***3.45***
**Sucrose synthase**			
BM260279	Sucrose synhase	0.000	−1.41
AY818397	*M. esculenta* sucrose synthase	0.001	***4.14***
**Vascular system**			
FG806684	GRP-like protein 2	0.000	***1.28***
FG806486	S-adenosylmethionine:2-demethylmenaquinone methyltransferase-like	0.004	***1.18***
FG806831	S-adenosyl methionine synthase-like	0.006	***1.57***
**Signaling**			
FG805239	Hexokinase	0.002	***1.80***
FG806368	Pyrophosphate-dependent phosphofructo-1-kinas…	0.000	***3.01***
**Growth and development**			
FG807559	Putative ripening-related protein	0.000	***2.27***
FG805555	Late embryogenesis abundant protein 5	0.000	3.54
FG807051	Late embryogenesis abundant protein Lea5	0.002	***2.05***
FG806890	Induced stolon tip protein	0.000	***2.52***
FG805555	Late embryogenesis abundant protein 5	0.000	***3.54***
FG806004	Expansin-like protein precursor	0.009	***9.18***

The plants were propagated by stem stakes and grown in the experimental fields of Agricultural College of Guangxi University in the 2010–2011 growing season following conventional cultivation and management.The expression of each gene in F01 and H124 was analyzed by qRT-PCR with a pair of cDNA sequence-specific primers, respectively. The figures in column ‘Fold change’ indicate a differential expression levels of the target genes in F01 stems under a comparison as F01 stems vs. H124 stems at the same growth stage, which was calculated following the formula of 2^−ΔΔCt(ΔCt^_F01 stems_^−ΔCt^_H124 stems_. The bold italic figures and negative figures in column ‘Fold change’ indicate the up-regulated and down-regulated expression levels of the genes, respectively, The up-regulated and down-regulated expression was determined according to a cutoff fold of ≧1.2 or ≦−1.2 at *p* < 0.01, respectively.
