# Supplementary material for: Characters related to higher starch accumulation in cassava storage roots
Source: Sci Rep. 2016 Feb 19;6:19823. doi: 10.1038/srep19823 (PMC4759534; doi:10.1038/srep19823)
Supplement: Supplementary Information [file srep19823-s1.pdf]

Characters related to higher starch accumulation in cassava storage roots

You-Zhi Li[**1], Jian-Yu Zhao[*1], San-Min Wu[1], Xian-Wei Fan[1], Xing-Lu Luo[1] & Bao-Shan Chen[**1]

[1]State Key Laboratory for Conservation and Utilization of Subtropical Agro-bioresources; Key Laboratory of Ministry of Education for Microbial and Plant Genetic Engineering; College of Life Science and Technology, Guangxi University; 100 Daxue Road, Nanning, Guangxi 530004, P. R. China

Supplementary Table S1 Description of 107 genes analyzed

Accession no.	Description
	Actin of cassava as internal control
FG805118	Peroxidase
BM260279	SUCROSE SYNTHASE
BM260275	SUCROSE SYNTHASE ISOFORM II
DQ443534	Sucrose synthase
FG805504	RHM1/ROL1 (RHAMNOSE BIOSYNTHESIS1); UDP-glucose 4,6-dehydratase/ catalytic
FG807216	Ethylene response factor
FG806385	Auxin response factor-like protein
FG806486	S-adenosylmethionine:2-demethylmenaquinone methyltransferase-like
FG806743	Secretory carrier membrane protein (SCAMP) family protein
FG806552	Auxin-responsive factor TIR1-like protein
DN740362	Similar to ethylene response factor
FG807559	Putative ripening-related protein
FG807051	Late embryogenesis abundant protein Lea5
FG806890	Induced stolon tip protein
FG806831	S-adenosyl methionine synthase-like
FG806176	Vacuolar protein sorting-associated protein
FG806323	S-adenosyl-L-methionine synthetase 1
FG807273	Expansin-like protein precursor
FG806078	Glucose-6-phosphate/phosphate-translocator
FG806269	Vacuolar sorting receptor protein
FG807417	Mechano sensitive ion channel domain-containing protein / MS ion channel domain-containing protein
FG805555	Late embryogenesis abundant protein 5
FG804941	Growth-on protein GRO10
FG804850	AXS1 (UDP-D-APIOSE/UDP-D-XYLOSE SYNTHASE 1)
FG807243	Fructose-bisphosphate aldolase, putative
FG805261	ATCSLC12 (Cellulose synthase-like C12); transferase, transferring glycosyl groups
FG804711	ATP:ADP antiporter/ binding
FG805045	Glucose-6-phosphate 1-dehydrogenase
FG805853	Ethylene response factor

FG807023	Putative shikimate kinase
FG806351	Golgi transport complex protein-related
FG805678	Chloroplast glucose-6-phosphate/phosphate translocator
FG807054	Regulator of gene silencing
FG806004	Expansin-like protein precursor
FG806888	Cellulose synthase catalytic subunit (UDP-forming)
FG806407	Nitrogen regulatory protein P-II 2
FG806208	Seed maturation protein PM36
FG807089	Secondary cell wall-related glycosyltransferase family 8
FG804956	Beta-glucosidase
FG806889	Bell division ATP-binding protein FtsE
FG805321	Inner-membrane translocator
FG805197	Solute carrier family 12 (potassium/chloride transporters), member 9
FG805211	Similar to Solute carrier family 22 (organic cation transporter)
FG807144	Glycosyl transferase, group 1
FG805259	Solute carrier family 2, facilitated glucose transporter member 12 (Glucose transporter type 12) (GLUT-12)
FG806102	Hexose carrier protein
DQ011041	*Manihot esculenta* alpha-amylase 2 (amy2) mRNA
DQ017830	*M. esculenta* alpha-amylase gene
X77012	*M. esculenta* SBE mRNA for starch branching enzyme
AY944584	*M. esculenta* beta-amylase (AmyB) gene
AY944583	*M. esculenta* alpha-amylase (AmyA) gene
X69714	*M. esculenta* mRNA for branching enzyme (r-3).
X74160	*M. esculenta* mRNA for granule-bound starch synthase
AF173900	*M. esculenta* granule bound starch synthase II precursor (GBSSII)
EF667961	*M. esculenta* starch synthase isoform II (SSII)
EF667960	*M. esculenta* starch synthase isoform I (SSI)
X74160	*M. esculenta* mRNA for granule-bound starch synthase
AY818397	*M. esculenta* sucrose synthase mRNA
DQ443534	*M. esculenta* sucrose synthase mRNA
FG807146	photosystem II CP47 protein
BI325240	**RUBISCO SUBUNIT BINDING-PROTEIN BETA SUBUNIT PRECURSOR**
FG806315	Auxin response factor 3
FG806257	Chitin synthase export chaperone
FG807524	Chitinase
FG806994	Chitin synthase
FG806895	Chitin synthase
FG807523	Chitin synthase
FG805882	Unnamed protein product
FG806486	S-adenosylmethionine:2-demethylmenaquinone methyltransferase-like
FG807035	Thiopurine S-methyltransferase
FG806240	UDP-glucose dehydrogenase
FG806843	Beta-fructofuranosidase
FG804902	Hypothetical protein
FG805122	Unnamed protein product
FG806288	Hypothetical protein
FG805239	Hexokinase
FG806020	Unnamed protein product

FG806684	GRP-like protein 2
FG806755	Unnamed protein product
GQ227726	Isoamylase isoform 3 mRNA
GU229751	Isoamylase (Meisa1) mRNA
FG806969	Hypothetical protein OsI_019972
FG806491	NADP-dependent malic protein
FG806272	Os01g0649100
FG805296	Pyruvate kinase, cytosolic isozyme
FG806882	Ribose-5-phosphate isomerase
FG806288	Hypothetical protein
FG806342	Arginine decarboxylase
FG807416	Glutamine synthetase
FG806408	AtTLP3 (TUBBY LIKE PROTEIN 3);
FG806896	Peptidase T2, asparaginase 2
FG806716	ATP synthase epsilon chain, mitochondrial
FG807008	Mitochondrial F1-ATPase, gamma subunit
FG805710	Mitochondrial succinate dehydrogenase iron-sulphur subunit
FG805581	Fumarate hydratase, putative / fumarase, putative
FG805377	Hypothetical protein
FG806045	Hypothetical protein PFL_3344
FG805615	Unnamed protein product
FG805564	Unknown
FG805866	6-phosphogluconate dehydrogenase
FG806368	Pyrophosphate-dependent phosphofructo-1-kinas...
FG805323	Unnamed protein product
FG805226	Hypothetical protein
FG805086	Unnamed protein product
FG805731	Unnamed protein product
FG804813	AP2/EREBP transcription factor ERF-2

Supplementary Table S2 Primers used in qPCR				
Accession no.	**F-Primer#**	**Primer(5'-3')**	**R-Primer#**	**Primer(5'-3')**
Actin	Me-QRP-90001_F	CCTTGACTATGAGCAGGAACTTG	Me-QRP-90001_R	CTTCTGGACAACGGAATCTTTC
FG805118	Me-QRP-00001_F	TGGGGCAACTGAACGTGTTGACA	Me-QRP-00001_R	CCTCTTCTGCGACGGACGCC
BM260279	Me-QRP-00002_F	TTCCAGGTAGCGGCGGCTCT	Me-QRP-00002_R	CCGAGGGAGCCATGCAACGT
BM260275	Me-QRP-00003_F	CCCACCGAGATGTATCTCCCTCGC	Me-QRP-00003_R	CCGGGGTTGGCCACCCTTTG
DQ443534	Me-QRP-00004_F	CCGGGGACCCCACTTCGTTG	Me-QRP-00004_R	TCACGGATGCTCTGAACGCGA
FG805504	Me-QRP-00005_F	AACCCTGGTGTGGTGAGCCA	Me-QRP-00005_R	TGTTGCTTCGTGGTGCCACT
FG807216	Me-QRP-00006_F	TGTGGAGCTTTGATGACCTCCCT	Me-QRP-00006_R	GCATGTCCAGGTGTGCTCTTACCA
FG806385	Me-QRP-00007_F	TGTTGGTGATGATCCCTGGCAGGA	Me-QRP-00007_R	TGCATCCATACCCTCCGCATCCA
FG806486	Me-QRP-00008_F	GGGAGGCAGTCCTGTTGTGCA	Me-QRP-00008_R	AAGCCAGAGCCCTAACACCCA
FG806743	Me-QRP-00009_F	GGCCTCTTTATCGTGCATGAGGACA	Me-QRP-00009_R	TGCTGGGAGTATGCCAGTCAAAGA
FG806552	Me-QRP-00010_F	CGGGCAGCGACTGTCACTTCT	Me-QRP-00010_R	CCCCTGGTCACTATCCCCAGCA
DN740362	Me-QRP-00011_F	TGGGAGGCTTCACTTGATGGCT	Me-QRP-00011_R	GGGAGGTCATCAAAGCTCCACAGG
FG807559	Me-QRP-00012_F	TGGGTACCCTGCAAAGGTTGCA	Me-QRP-00012_R	GGCGGAAATGGGCACACCCT
FG807051	Me-QRP-00013_F	AGAGCGAGCCGCAAGCTTGG	Me-QRP-00013_R	TAGCGTTGGCGGGAGAGCGA
FG806890	Me-QRP-00014_F	ATTCCACGACGACAGCCGCG	Me-QRP-00014_R	CGGATGAAACACCGAGGGCCG
FG806831	Me-QRP-00015_F	GCGCTACCTGTAGCAGGCTGC	Me-QRP-00015_R	ACAACACGCCACTGTTCTGGCA
FG806176	Me-QRP-00016_F	GACAGAGCAGCAGGCTCGGC	Me-QRP-00016_R	GCAATTGGGCAAAGCCGCCA
FG806323	Me-QRP-00017_F	AGAGGGGTGGCAGTGGTAGGT	Me-QRP-00017_R	CGGCGATGGCAAGAGTTTTACGC
FG807273	Me-QRP-00018_F	GCGCAAAGAGTGTCTTCCTGCAGA	Me-QRP-00018_R	GGGAGAGCAACCCTCTTGCGC
FG806078	Me-QRP-00019_F	CAATGCCCCTTGGAGCCGCCA	Me-QRP-00019_R	CGGTCACCAATCATTCCGGCCA
FG806269	Me-QRP-00020_F	GGCTTGGGAATGGCTGCTGGT	Me-QRP-00020_R	TCAGGCACGTTCGTCGTTGACA
FG807417	Me-QRP-00021_F	CAGGAGCCCACCAATGGGCG	Me-QRP-00021_R	TCAGCTCGCCACTTTCTCGGA
FG805555	Me-QRP-00022_F	GGAGGGGATTCAGCGGCTGC	Me-QRP-00022_R	AGCATCCGGTGCGAGGGTCT
FG804941	Me-QRP-00023_F	TGGACATGGGGGCAAGCTCT	Me-QRP-00023_R	GGATGGCATGGCCAACCTGCA
FG804850	Me-QRP-00024_F	TCCAGCGTCAATAGTCAATGCCTCT	Me-QRP-00024_R	CGCAGCCCAAGCTCACAGGG
FG807243	Me-QRP-00025_F	GCTCTCTGCAGCACCCTCGC	Me-QRP-00025_R	AGCTTGGCGGCAGAGGACAA
FG805261	Me-QRP-00026_F	GTTGATGAAGGCAACACGC	Me-QRP-00026_R	TGTTCTGGAGGAAGGAGGC
FG804711	Me-QRP-00027_F	CGGCGGTGAAATGAGGCTGC	Me-QRP-00027_R	AACCGGCAAGCACACCAGCA
FG805045	Me-QRP-00028_F	CGGGGTCGCGATTACCCCTG	Me-QRP-00028_R	CCCCAACAGGGCCCCGACTA
FG805853	Me-QRP-00029_F	GGTGCCCCGTCAACAGCACA	Me-QRP-00029_R	TCCCAACGTCCACGCGAGGA
FG807023	Me-QRP-00030_F	TCCAGGTGCTTTGTGACGTGCT	Me-QRP-00030_R	GCGACATGCAATGTTCCGCCA
FG806351	Me-QRP-00031_F	CGGAGCATTCTTCTCCGGGCG	Me-QRP-00031_R	CCTTCGGTGTGTCAACGGCGT
FG805678	Me-QRP-00032_F	GCTGCTGGCTCGTCCAGTCC	Me-QRP-00032_R	TGGGTGCTTTGCGAGCGTTGA
FG807054	Me-QRP-00033_F	TGACATGGACGGAGATGGGCT	Me-QRP-00033_R	AGCCAAGGCTCCGGTTTCGT
FG806004	Me-QRP-00034_F	TGACAGATTGGAAGCCAGGA	Me-QRP-00034_R	AGAGCAACCCTCCTTTTGC
FG806888	Me-QRP-00035_F	CGGGGCATGGCAATCGGTGT	Me-QRP-00035_R	TCCTCCTGTGGGATTGTGGCGT
FG806407	Me-QRP-00036_F	TGCCGGTGTTGGCCGACTTG	Me-QRP-00036_R	TGCCATTGACGACCAAAAGGGGA
FG806208	Me-QRP-00037_F	TGTCTCGCCAGAGATTGCCGA	Me-QRP-00037_R	TTCAGACACCCCCACGACTCA
FG807089	Me-QRP-00038_F	GAATCAATAACTTCAGCAGCACA	Me-QRP-00038_R	GCTTCCACAGTACCCAAACATG
FG804956	Me-QRP-00039_F	GTCACAGACCAACGTACCCTACA	Me-QRP-00039_R	GAGTCCCGCGAAACATCA
FG806889	Me-QRP-00040_F	TGGCGTCCACAGCTCAAAGTCG	Me-QRP-00040_R	ACCTTCGTCGAGCACTGGGA
FG805321	Me-QRP-00041_F	TGGGGCTTGAAAAGCTTGGGGG	Me-QRP-00041_R	ACTTGCCGGTCTGCCATGGC
FG805197	Me-QRP-00042_F	GTGTTCGGATTTGTGTAGCGGTGT	Me-QRP-00042_R	TTCTCACTTCCCACCTTCTGTGTT
FG805211	Me-QRP-00043_F	TGGGCAGCAAGTTACGGGGA	Me-QRP-00043_R	GCCTCTGGCGGCTCATCTCC
FG807144	Me-QRP-00044_F	GGTCGTAACTTCGCTCGAGAGGC	Me-QRP-00044_R	AGGGGATCAAACCACGAGAGTGGA

FG805259	Me-QRP-00045_F	GGACCCTAATGCTTGCTTGGGGG	Me-QRP-00045_R	TGTGCAGGCAGCCATCTTATTTGC
FG806102	Me-QRP-00046_F	GCCTTTGTGTACTTCTTGCTGCCG	Me-QRP-00046_R	TCCAGAACCAGTGCTCCCTCCA
DQ011041	Me-QRP-00047_F	GGGTAACCGCAGCACTGGGG	Me-QRP-00047_R	GGAGCCACTGCAGCCATGCT
DQ017830	Me-QRP-00048_F	GAATCATGTAACAAGGCAGAAGG	Me-QRP-00048_R	CTGACTGAGAAGGAGGAGGAAG
X77012	Me-QRP-00049_F	TTGGGTCTGTTTCCGGCGCC	Me-QRP-00049_R	AGGGATCTAGGACCACCAGGCA
AY944584	Me-QRP-00050_F	ACGTCCACTGGAGCGGTCAA	Me-QRP-00050_R	CCATGGGAAGGGTGCCAATGCC
AY944583	Me-QRP-00051_F	AGGAAAGTTGACAGCGCGGGA	Me-QRP-00051_R	TAAAGGCTGCCCTGCGACGC
X69714	Me-QRP-00052_F	TGGTTGCGACTTGCCCGGAA	Me-QRP-00052_R	TGGCCCACTCTTCCACGTCCA
X74160	Me-QRP-00053_F	ACCGGAGAGAGCACCATGGCA	Me-QRP-00053_R	TGGGTCCAGGGTCCAGTGTGA
AF173900	Me-QRP-00054_F	GCTCCGGTTTGCCATCCCCC	Me-QRP-00054_R	TCCTGACCCACCATCCAGGGA
EF667961	Me-QRP-00055_F	GGGTCAGTCCCGCTTTGCACT	Me-QRP-00055_R	CTCACAAGTGCGACAGCCCC
EF667960	Me-QRP-00056_F	ATGGCCGCTTGTGCCCATCG	Me-QRP-00056_R	TCGTGAACTGCCAAGCCCACC
X74160	Me-QRP-00053_F	ACCGGAGAGAGCACCATGGCA	Me-QRP-00053_R	TGGGTCCAGGGTCCAGTGTGA
AY818397	Me-QRP-00058_F	TGTACCATTGCTCATGCTCTCGAGA	Me-QRP-00058_R	GCCGTAAACTGGCATGAAAAGTGGT
DQ443534	Me-QRP-00004_F	CCGGGGACCCCACTTCGTTG	Me-QRP-00004_R	TCACGGATGCTCTGAACGCGA
FG807146	Me-QRP-00060_F	TTCAGGCCTGGCTCCGTTTCT	Me-QRP-00060_R	TGGATCAACAACCCATAAGCACAAA
BI325240	Me-QRP-00061_F	GATTGCCAGCAGGCACCGGT	Me-QRP-00061_R	GCTGCCTAGAGCATGCATCATCGG
FG806315	Me-QRP-00062_F	CCTTCCAGAGGTGGGAACTTAC	Me-QRP-00062_R	CCTTGGCACGAAAGGAATAAG
FG806257	Me-QRP-00063_F	CCAGCTCGATGCTACGCGCA	Me-QRP-00063_R	TCTGCTCCCTGGTCGGACCG
FG807524	Me-QRP-00064_F	CCGTTGGAAATCGAATGGGGAACC	Me-QRP-00064_R	CCCGGGCAAACCCCAAAGGG
FG806994	Me-QRP-00065_F	CGATCATGGGCGAGCTTATAC	Me-QRP-00065_R	AGAACTGCAAATTCCTTAGACAAC
FG806895	Me-QRP-00066_F	GGGCGAGCTTATCGAATTCTATCCC	Me-QRP-00066_R	TAGAGGCCGAGGCGTGCGA
FG807523	Me-QRP-00067_F	GAGCTTATAAATATCTATCGAATCTTG	Me-QRP-00067_R	GAAAAAGGGAGAGAGAGAACTGC
FG805882	Me-QRP-00068_F	GGTGGGCGATGGTGTGGGAC	Me-QRP-00068_R	GCGCTGGAGTGGCATGGCTT
FG806486	Me-QRP-00008_F	GGGAGGCAGTCCTGTTGTGCA	Me-QRP-00008_R	AAGCCAGAGCCCTAACACCCA
FG807035	Me-QRP-00070_F	GACCGGCCAAAGGGACA	Me-QRP-00070_R	GAGCGATTGCACAGCACTCTA
FG806240	Me-QRP-00071_F	GCTCAAGGACCTGCCTGCTGT	Me-QRP-00071_R	CTGCAGCCAGTTGACCCGCA
FG806843	Me-QRP-00072_F	CCGTTGCTGAAAGATAGCTGGCT	Me-QRP-00072_R	ACCCAGCAATCGACCATGTCTGG
FG804902	Me-QRP-00073_F	GCCTCCACGCCCAGCATTCA	Me-QRP-00073_R	GGGGCAATGCCTGCTCAGCA
FG805122	Me-QRP-00074_F	GACTTCACAGGTAGGGAGCAG	Me-QRP-00074_R	GCTACAGAACCGCCAAGATTG
FG806288	Me-QRP-00075_F	CATTCGGCGGGCATGATGCA	Me-QRP-00075_R	TGCCAAAACCCTTTCAAGCAGCT
FG805239	Me-QRP-00076_F	TCCGTAGCTCCATGCGAGAGCA	Me-QRP-00076_R	GCTGCAAGGAGAGCCGCTCC
FG806020	Me-QRP-00077_F	TGGTGGGTGTCGTCACGGGT	Me-QRP-00077_R	GTGTTCGGTTCTGGCGACCCA
FG806684	Me-QRP-00078_F	TCTCATGGGCGATGGTCAGCCA	Me-QRP-00078_R	CCAAAGCCCAAGTGGTCGCA
FG806755	Me-QRP-00079_F	CAGTGTGGCAGTTCTAATGGAGGGG	Me-QRP-00079_R	TGCAGATCCCCAGGGCAAGC
GQ227726	Me-QRP-00080_F	ATGGGCCGAGTGGAACGGGA	Me-QRP-00080_R	TCGCGCTGTACAGGTCAGCA
GU229751	Me-QRP-00081_F	AAGCATGGGATGCGGGAGGC	Me-QRP-00081_R	GCGAAAGCCCCAGCAAAGCC
FG806969	Me-QRP-00082_F	CACTCGATGGTGCCCGGCTG	Me-QRP-00082_R	TCCCCATCCTTCACTGCGTCT
FG806491	Me-QRP-00083_F	GCTGCCCAGGTGACCCAGGA	Me-QRP-00083_R	AGCAGCCACATTGGCCGCAA
FG806272	Me-QRP-00084_F	CCTTCCACCACCACACGCCC	Me-QRP-00084_R	CCGGTGCTACTGGCGAAGCA
FG805296	Me-QRP-00085_F	GCCAGGTGATGCTGTTGTGGCA	Me-QRP-00085_R	CTCTTGCAACGGCCTCGCCA
FG806882	Me-QRP-00086_F	GGCTAGCAGGTGTCGTTGAGCA	Me-QRP-00086_R	AGTGACCCCAAGTTCCCCTGCA
FG806288	Me-QRP-00075_F	CATTCGGCGGGCATGATGCA	Me-QRP-00075_R	TGCCAAAACCCTTTCAAGCAGCT
FG806342	Me-QRP-00088_F	TCTTAAGAGGAGGTGGGGGAGAGA	Me-QRP-00088_R	AGAGTGCAAGCAAAACTCGCCA
FG807416	Me-QRP-00089_F	TGGGGTGTTGCTAACCGCGG	Me-QRP-00089_R	TGATGCAGGCCTCCTGTCCTCA
FG806408	Me-QRP-00090_F	TGGCGACCCATGTGGCATGA	Me-QRP-00090_R	TCGGCAGAAGCAACCAGCTGA
FG806896	Me-QRP-00091_F	AGGTCGCAGCAGCACAACGG	Me-QRP-00091_R	GCTGCGGCATGGAGAGTCCC
FG806716	Me-QRP-00092_F	AGACGAAGCCTCACTCGCGA	Me-QRP-00092_R	ACGAAGAGTGGGTTTCTGGGGCT
FG807008	Me-QRP-00093_F	CCTCGAGTGCAGATGCTCCCG	Me-QRP-00093_R	TGCTGGCGAGATGCTTGATCGT

FG805710	Me-QRP-00094_F	TGGGGCCAGCTGCTTTGCTC	Me-QRP-00094_R	TTGGACAGGCACGGGCACAA
FG805581	Me-QRP-00095_F	ATAGGGAATAATGGTGAGTGGTG	Me-QRP-00095_R	ATGTTCCTAATCCGACAAATAAGAG
FG805377	Me-QRP-00096_F	GGCCAAGATGCGCTCTCTGGG	Me-QRP-00096_R	TGGCCTGCTTGCAAATGCCTGT
FG806045	Me-QRP-00097_F	TGCGCAATGAGCCGCGAGAA	Me-QRP-00097_R	TGGGGGTGATCAGCCAGCCA
FG805615	Me-QRP-00098_F	GTGAGGCAAAACCACACCCCCA	Me-QRP-00098_R	GCGGGGGCTCGTGTGGAAAA
FG805564	Me-QRP-00099_F	GAGCGTCAGTCTGCCTGGCG	Me-QRP-00099_R	GCTGGCAACCTTTCCCTTCGGT
FG805866	Me-QRP-00100_F	GGACCATCGAGGCCGCCATG	Me-QRP-00100_R	TTTGCTGGCGGGAGCGGAAG
FG806368	Me-QRP-00101_F	TGGGAATTGTGCTGACGACCTCA	Me-QRP-00101_R	ACCGGGATCTCGCGGTAGGT
FG805323	Me-QRP-00102_F	TCGAGGGAAGGCTGCAGTTGC	Me-QRP-00102_R	ACCCTCTGCTTCTTGGCACCCT
FG805226	Me-QRP-00103_F	GGCAGGCAGGTTGGCATCATGT	Me-QRP-00103_R	TCTCCAGCAAAAGATGCCTTGAGCC
FG805086	Me-QRP-00104_F	TGGTTTGCCGACCAGTCTCCTGT	Me-QRP-00104_R	TGGGTTGCAAGGGTAAGGGCA
FG805731	Me-QRP-00105_F	TCCGCCACTGTCAAGAAGGAGT	Me-QRP-00105_R	CCTTGGGCAAGCAAGGGCCA
FG804813	Me-QRP-00106_F	TGTCCGTGTCTGGCTCGGGA	Me-QRP-00106_R	TTGTCGCCACGGATGCGCTT

Supplementary Table S3 qPCR cycles of the genes in H124 at ‘seedling’

	Sample	H124 leaves				H124 stems				H124 roots			
	primer ID	Ct1	Ct2	Ct3	Ave.Ct	Ct1	Ct2	Ct3	Ave.Ct	Ct1	Ct2	Ct3	Ave.Ct
1	Me-QRP-90001	24.18	23.99	23.94	24.04	24.71	24.72	24.61	24.68	24.47	24.41	24.57	24.48
2	Me-QRP-00001	27.11	27.07	27.03	27.07	25.88	25.80	25.76	25.81	23.05	23.16	23.21	23.14
3	Me-QRP-00002	28.73	28.59	28.62	28.65	26.05	26.01	25.88	25.98	22.73	22.83	22.73	22.76
4	Me-QRP-00003	0.00	0.00	0.00	0.00	0.00	0.00	0.00	0.00	0.00	0.00	0.00	0.00
5	Me-QRP-00004	33.59	32.81	32.95	33.12	27.25	26.95	26.85	27.02	25.52	25.77	25.82	25.70
6	Me-QRP-00005	26.07	25.77	25.81	25.89	27.76	27.38	27.40	27.52	25.51	25.49	25.74	25.58
7	Me-QRP-00006	29.57	28.77	29.01	29.12	28.69	28.33	28.22	28.41	23.61	23.49	23.45	23.52
8	Me-QRP-00007	28.88	28.97	28.70	28.85	29.64	29.72	29.64	29.67	25.47	25.58	25.55	25.53
9	Me-QRP-00008	28.44	28.07	28.02	28.18	28.01	27.79	27.80	27.87	24.35	24.07	24.59	24.33
10	Me-QRP-00009	30.03	30.08	30.11	30.07	26.89	26.76	26.78	26.81	24.96	24.95	25.12	25.01
11	Me-QRP-00010	32.33	32.06	32.23	32.20	33.92	33.66	33.23	33.60	31.75	31.92	31.62	31.76
12	Me-QRP-00011	27.51	27.26	27.27	27.35	27.65	27.18	27.13	27.32	23.49	23.53	23.41	23.48
13	Me-QRP-00012	23.92	23.56	23.73	23.74	28.37	28.24	27.98	28.20	26.74	26.71	24.26	25.90
14	Me-QRP-00013	29.70	29.48	29.28	29.49	29.75	29.83	29.63	29.73	26.93	26.99	27.01	26.98
15	Me-QRP-00014	27.03	26.91	26.79	26.91	26.82	26.52	26.31	26.55	25.02	25.13	25.15	25.10
16	Me-QRP-00015	26.28	26.10	26.13	26.17	26.30	26.37	26.10	26.26	23.91	23.81	23.83	23.85
17	Me-QRP-00016	27.99	27.92	27.87	27.93	26.92	26.97	26.85	26.91	26.84	26.72	27.49	27.02
18	Me-QRP-00017	26.24	26.17	26.45	26.29	25.67	25.29	25.16	25.37	24.93	25.07	24.99	25.00
19	Me-QRP-00018	28.82	28.81	28.76	28.80	27.50	27.45	27.33	27.43	25.25	25.38	25.38	25.34
20	Me-QRP-00019	29.31	29.14	29.37	29.28	30.90	31.28	30.95	31.04	30.75	30.21	30.27	30.41
21	Me-QRP-00020	28.18	27.73	27.74	27.89	28.75	28.79	28.66	28.73	26.67	26.80	26.77	26.74
22	Me-QRP-00021	31.45	31.52	31.32	31.43	35.18	34.06	34.06	34.43	31.51	31.16	31.13	31.27
23	Me-QRP-00022	24.15	23.83	23.94	23.97	29.09	28.79	28.73	28.87	26.57	26.26	26.28	26.37
24	Me-QRP-00023	27.74	27.61	27.57	27.64	27.60	27.61	27.40	27.54	25.77	25.72	25.80	25.76
25	Me-QRP-00024	28.47	28.44	28.24	28.39	28.31	28.40	28.26	28.32	25.82	25.60	25.89	25.77
26	Me-QRP-00025	37.63	36.94	35.83	36.80	35.54	35.54	35.64	35.57	33.10	32.96	33.69	33.25
27	Me-QRP-00026	29.99	30.02	30.00	30.01	31.87	31.20	31.60	31.56	29.10	29.12	29.01	29.08
28	Me-QRP-00027	26.93	26.75	26.99	26.89	28.27	28.12	27.98	28.12	26.25	26.44	26.52	26.40
29	Me-QRP-00028	31.79	31.45	31.76	31.67	37.77	37.52	36.32	37.20	28.96	28.63	28.56	28.72
30	Me-QRP-00029	0.00	0.00	0.00	0.00	0.00	0.00	0.00	0.00	0.00	0.00	0.00	0.00
31	Me-QRP-00030	29.89	29.39	29.53	29.60	31.50	31.56	31.24	31.44	29.47	29.43	29.31	29.40
32	Me-QRP-00031	29.98	29.82	29.87	29.89	31.10	31.13	30.94	31.05	29.05	29.04	29.02	29.04
33	Me-QRP-00032	27.75	27.66	27.74	27.72	27.55	27.59	27.44	27.53	24.90	24.93	24.98	24.94
34	Me-QRP-00033	31.24	30.84	31.29	31.12	29.35	29.18	29.14	29.22	27.71	27.79	27.73	27.74
35	Me-QRP-00034	33.62	31.76	31.95	32.45	33.45	33.14	32.71	33.10	0.00	0.00	0.00	0.00
36	Me-QRP-00035	0.00	0.00	0.00	0.00	0.00	0.00	0.00	0.00	0.00	37.99	0.00	0.00
37	Me-QRP-00036	0.00	0.00	0.00	0.00	0.00	0.00	0.00	0.00	0.00	0.00	0.00	0.00
38	Me-QRP-00037	32.42	32.22	32.02	32.22	32.95	32.99	32.76	32.90	31.08	30.99	31.01	31.03
39	Me-QRP-00038	29.87	29.88	29.93	29.89	31.81	31.72	31.38	31.64	28.97	28.71	28.90	28.86
40	Me-QRP-00039	0.00	0.00	0.00	0.00	0.00	0.00	0.00	0.00	0.00	0.00	0.00	0.00
41	Me-QRP-00040	36.92	36.12	36.60	36.55	35.78	N/A	38.27	37.02	37.39	38.21	37.68	37.76
42	Me-QRP-00041	28.91	28.37	28.49	28.59	24.87	24.83	24.70	24.80	24.28	24.28	24.37	24.31
43	Me-QRP-00042	31.42	31.23	31.09	31.25	32.55	32.56	32.41	32.51	32.35	32.26	32.14	32.25
44	Me-QRP-00043	26.91	27.00	26.87	26.93	26.69	26.82	26.74	26.75	25.69	25.71	25.80	25.73
45	Me-QRP-00044	0.00	0.00	0.00	0.00	0.00	0.00	0.00	0.00	0.00	0.00	0.00	0.00
46	Me-QRP-00045	27.32	27.31	27.22	27.29	28.38	28.43	27.98	28.26	26.09	26.20	26.35	26.21
47	Me-QRP-00046	30.49	30.43	30.43	30.45	33.32	33.47	32.91	33.23	30.12	30.22	30.31	30.22
48	Me-QRP-00047	27.66	27.26	27.45	27.45	28.89	28.91	28.98	28.93	28.83	28.77	28.43	28.68
49	Me-QRP-00048	31.22	31.23	31.16	31.20	35.45	35.31	36.23	35.66	32.61	32.70	33.03	32.78
50	Me-QRP-00049	31.91	32.04	31.59	31.85	30.87	30.67	30.91	30.82	23.33	25.57	25.16	24.68
51	Me-QRP-00050	29.72	29.73	29.48	29.64	36.15	37.02	37.18	36.78	30.86	30.94	31.20	31.00
52	Me-QRP-00051	0.00	0.00	0.00	0.00	0.00	0.00	0.00	0.00	0.00	0.00	37.56	37.56
53	Me-QRP-00052	28.43	28.23	28.19	28.28	27.91	27.72	27.73	27.79	26.95	27.16	27.12	27.08
54	Me-QRP-00053	23.55	23.34	23.44	23.45	35.02	35.75	35.77	35.52	28.27	28.47	28.36	28.37
55	Me-QRP-00054	25.56	25.25	25.28	25.36	28.40	28.18	28.01	28.20	29.05	29.07	28.85	28.99
56	Me-QRP-00055	24.95	24.60	24.61	24.72	27.56	27.44	27.47	27.49	30.16	29.95	30.36	30.16
57	Me-QRP-00056	28.96	28.76	28.67	28.80	30.06	29.90	29.60	29.85	30.04	29.96	29.96	29.99
58	Me-QRP-00053	23.55	23.34	23.44	23.45	35.02	35.75	35.77	35.52	28.27	28.47	28.36	28.37
59	Me-QRP-00058	26.28	25.67	25.51	25.82	0.00	0.00	0.00	0.00	25.59	25.68	25.58	25.62
60	Me-QRP-00004	33.59	32.81	32.95	33.12	27.25	26.95	26.85	27.02	25.52	25.77	25.82	25.70

61	Me-QRP-00060	27.42	27.09	27.14	27.22	29.95	30.01	29.71	29.89	27.93	28.20	28.07	28.06
62	Me-QRP-00061	23.97	23.73	23.60	23.77	25.37	25.02	24.93	25.11	26.58	26.79	26.88	26.75
63	Me-QRP-00062	0.00	0.00	0.00	0.00	0.00	0.00	0.00	0.00	0.00	0.00	0.00	0.00
64	Me-QRP-00063	0.00	0.00	0.00	0.00	0.00	0.00	0.00	0.00	35.80	36.84	35.72	36.12
65	Me-QRP-00064	30.23	29.92	30.06	30.07	29.55	29.45	29.33	29.45	27.04	27.14	27.00	27.06
66	Me-QRP-00065	30.87	30.30	30.15	30.44	32.61	31.79	31.38	31.93	29.42	29.72	29.55	29.57
67	Me-QRP-00066	0.00	0.00	0.00	0.00	0.00	0.00	0.00	0.00	38.01	0.00	0.00	0.00
68	Me-QRP-00067	0.00	0.00	0.00	0.00	0.00	0.00	0.00	0.00	0.00	0.00	0.00	0.00
69	Me-QRP-00068	0.00	0.00	0.00	0.00	0.00	0.00	0.00	0.00	0.00	0.00	0.00	0.00
70	Me-QRP-00008	28.44	28.07	28.02	28.18	28.01	27.79	27.80	27.87	24.35	24.07	24.59	24.33
71	Me-QRP-00070	0.00	0.00	0.00	0.00	0.00	0.00	0.00	0.00	0.00	0.00	0.00	0.00
72	Me-QRP-00071	28.14	27.53	27.89	27.85	25.98	25.85	25.81	25.88	24.63	24.78	24.75	24.72
73	Me-QRP-00072	0.00	0.00	0.00	0.00	0.00	0.00	0.00	0.00	0.00	0.00	0.00	0.00
74	Me-QRP-00073	27.86	27.75	27.53	27.71	27.35	27.12	27.08	27.18	28.15	27.97	27.88	28.00
75	Me-QRP-00074	29.17	29.19	28.97	29.11	31.41	31.72	31.40	31.51	27.30	27.61	27.60	27.51
76	Me-QRP-00075	24.08	24.00	23.98	24.02	25.10	25.22	25.07	25.13	23.29	23.23	23.35	23.29
77	Me-QRP-00076	27.31	27.39	27.55	27.41	29.43	29.13	29.21	29.26	27.99	28.01	28.07	28.02
78	Me-QRP-00077	27.72	27.89	27.31	27.64	27.56	27.26	27.19	27.34	26.95	26.96	26.93	26.95
79	Me-QRP-00078	28.65	28.53	28.50	28.56	29.02	28.92	29.02	28.98	28.75	28.73	28.96	28.82
80	Me-QRP-00079	28.65	28.53	28.50	28.56	29.02	28.92	29.02	28.98	29.74	29.50	29.90	29.72
81	Me-QRP-00080	33.88	33.35	32.81	33.35	33.11	33.15	32.78	33.01	30.12	30.37	30.39	30.29
82	Me-QRP-00081	29.81	28.71	29.54	29.35	29.15	29.15	27.99	28.76	27.98	28.03	28.07	28.03
83	Me-QRP-00082	28.13	28.26	28.20	28.20	29.41	29.57	29.44	29.47	28.29	28.37	28.40	28.35
84	Me-QRP-00083	29.64	29.41	29.02	29.36	28.69	28.23	28.06	28.33	28.57	28.50	28.55	28.54
85	Me-QRP-00084	25.92	25.71	25.65	25.76	28.35	28.08	28.03	28.15	26.54	26.70	26.60	26.62
86	Me-QRP-00085	26.24	26.15	26.14	26.18	27.53	27.24	26.90	27.22	25.58	25.68	25.58	25.62
87	Me-QRP-00086	27.24	27.08	27.12	27.15	26.06	26.07	26.01	26.05	26.70	26.25	26.43	26.46
88	Me-QRP-00075	24.08	24.00	23.98	24.02	25.10	25.22	25.07	25.13	23.29	23.23	23.35	23.29
89	Me-QRP-00088	27.38	27.03	27.12	27.18	26.88	26.67	26.62	26.72	25.25	25.36	25.40	25.34
90	Me-QRP-00089	28.15	27.89	27.87	27.97	28.23	27.94	27.75	27.97	26.58	26.66	26.58	26.61
91	Me-QRP-00090	30.65	30.36	29.23	30.08	31.27	31.64	31.19	31.36	31.56	31.72	31.59	31.63
92	Me-QRP-00091	26.20	26.04	25.91	26.05	29.20	29.15	28.99	29.11	28.03	27.92	28.07	28.01
93	Me-QRP-00092	28.32	28.13	27.97	28.14	28.23	27.88	27.72	27.94	26.10	25.98	26.20	26.10
94	Me-QRP-00093	27.95	27.57	27.69	27.74	28.68	28.28	28.20	28.39	26.08	26.13	26.13	26.11
95	Me-QRP-00094	30.61	30.15	30.85	30.54	30.21	29.97	29.70	29.96	26.45	26.36	26.50	26.44
96	Me-QRP-00095	26.92	26.72	26.75	26.80	28.79	28.92	28.50	28.74	25.98	26.08	26.19	26.08
97	Me-QRP-00096	26.59	26.26	26.40	26.42	28.18	28.38	28.11	28.22	27.70	27.55	27.48	27.58
98	Me-QRP-00097	0.00	0.00	0.00	0.00	0.00	0.00	0.00	0.00	0.00	0.00	0.00	0.00
99	Me-QRP-00098	27.57	27.44	27.21	27.41	26.97	27.11	27.11	27.06	24.96	25.08	25.04	25.03
100	Me-QRP-00099	29.16	29.44	29.24	29.28	37.13	37.99	36.69	37.27	27.34	27.42	27.44	27.40
101	Me-QRP-00100	0.00	0.00	0.00	0.00	0.00	0.00	0.00	0.00	33.91	32.82	32.92	33.22
102	Me-QRP-00101	33.79	33.82	33.55	33.72	36.05	35.66	35.97	35.90	32.06	32.07	32.17	32.10
103	Me-QRP-00102	30.29	29.74	29.78	29.94	28.10	27.79	27.65	27.84	26.79	26.73	26.71	26.74
104	Me-QRP-00103	29.89	29.72	29.64	29.75	29.74	29.61	29.33	29.56	28.58	28.29	28.24	28.37
105	Me-QRP-00104	27.38	27.04	26.85	27.09	28.40	28.04	27.84	28.10	24.21	24.16	24.02	24.13
106	Me-QRP-00105	26.80	26.63	26.50	26.65	28.19	28.37	28.05	28.20	25.75	25.65	25.59	25.66
107	Me-QRP-00106	29.73	29.34	29.58	29.55	29.78	29.31	29.15	29.41	27.93	27.85	27.75	27.84

**Supplementary Table S4** qPCR cycles of the genes in F01 at 'seedling'													
	Sample	F01 leaves				F01 stems				F01 roots			
	primer ID	Ct1	Ct2	Ct3	Ave.Ct	Ct1	Ct2	Ct3	Ave.Ct	Ct1	Ct2	Ct3	Ave.Ct
1	Me-QRP-90001	24.06	24.10	23.97	24.04	23.26	23.13	23.04	23.14	24.30	24.20	23.85	24.12
2	Me-QRP-00001	26.66	26.64	26.68	26.66	23.45	23.25	23.21	23.30	21.90	22.06	21.92	21.96
3	Me-QRP-00002	28.77	28.57	28.79	28.71	25.08	24.92	24.86	24.95	22.05	22.01	22.00	22.02
4	Me-QRP-00003	0.00	0.00	0.00	0.00	0.00	0.00	0.00	0.00	0.00	37.92	0.00	0.00
5	Me-QRP-00004	32.59	32.34	32.14	32.36	26.12	25.92	25.84	25.96	25.01	24.85	24.92	24.92
6	Me-QRP-00005	25.89	25.52	25.47	25.63	24.50	24.18	24.10	24.26	25.03	24.90	24.94	24.96
7	Me-QRP-00006	28.52	28.46	28.43	28.47	25.73	25.56	25.56	25.62	22.88	22.85	22.76	22.83
8	Me-QRP-00007	29.32	29.14	29.27	29.24	27.32	27.23	27.14	27.23	25.63	25.44	25.19	25.42
9	Me-QRP-00008	28.07	27.93	27.93	27.97	26.51	26.32	26.11	26.32	23.70	23.46	23.57	23.58
10	Me-QRP-00009	25.71	25.91	25.84	25.82	29.42	29.19	29.34	29.32	24.15	24.11	24.08	24.11
11	Me-QRP-00010	33.21	32.93	32.95	33.03	32.16	31.98	31.58	31.91	31.53	31.26	31.36	31.38
12	Me-QRP-00011	27.31	27.37	27.31	27.33	25.36	25.19	25.14	25.23	22.88	22.84	22.66	22.79
13	Me-QRP-00012	24.03	24.03	23.97	24.01	26.50	26.21	26.12	26.28	23.39	22.53	20.29	22.07
14	Me-QRP-00013	28.53	28.39	28.55	28.49	24.00	23.99	24.05	24.01	26.43	26.41	26.20	26.34
15	Me-QRP-00014	26.52	26.47	26.75	26.58	23.60	23.46	23.76	23.61	24.98	24.88	24.92	24.93
16	Me-QRP-00015	26.63	26.40	26.41	26.48	23.75	23.71	23.76	23.74	22.68	22.74	22.68	22.70
17	Me-QRP-00016	28.38	28.25	28.25	28.30	24.75	24.62	24.42	24.59	26.98	26.79	26.84	26.87
18	Me-QRP-00017	25.49	26.10	26.04	25.88	22.76	22.34	22.25	22.45	24.52	24.59	24.29	24.47
19	Me-QRP-00018	28.87	28.83	28.85	28.85	24.11	23.93	23.86	23.97	25.60	25.42	25.45	25.49
20	Me-QRP-00019	28.41	28.24	28.27	28.31	27.58	27.33	27.30	27.40	29.84	29.44	29.31	29.53
21	Me-QRP-00020	28.07	27.99	27.93	28.00	26.82	26.58	26.47	26.62	26.18	26.12	26.17	26.15
22	Me-QRP-00021	31.86	31.81	31.86	31.84	31.57	31.07	31.01	31.22	31.04	31.21	31.16	31.14
23	Me-QRP-00022	23.66	23.62	23.50	23.59	25.21	25.10	25.03	25.11	26.53	26.61	26.44	26.53
24	Me-QRP-00023	28.24	28.10	28.16	28.17	26.23	26.07	26.04	26.11	25.86	25.58	25.43	25.63
25	Me-QRP-00024	28.14	28.21	28.22	28.19	25.94	25.77	25.59	25.77	24.73	24.75	24.65	24.71
26	Me-QRP-00025	36.89	36.78	36.06	36.58	33.31	33.44	33.72	33.49	32.11	32.13	32.27	32.17
27	Me-QRP-00026	30.74	30.63	30.45	30.61	28.50	28.26	28.40	28.39	28.41	28.25	28.32	28.33
28	Me-QRP-00027	26.94	27.00	26.94	26.96	25.86	25.73	25.80	25.80	25.46	25.19	25.20	25.28
29	Me-QRP-00028	32.43	31.86	32.51	32.27	35.10	36.14	35.71	35.65	27.33	27.25	27.24	27.27
30	Me-QRP-00029	0.00	0.00	0.00	0.00	0.00	0.00	0.00	0.00	0.00	0.00	0.00	0.00
31	Me-QRP-00030	29.84	29.89	29.90	29.88	29.58	29.54	29.37	29.50	28.27	28.30	28.43	28.33
32	Me-QRP-00031	30.73	30.41	30.35	30.49	29.19	29.38	29.10	29.22	28.31	28.03	28.08	28.14
33	Me-QRP-00032	28.81	28.60	28.75	28.72	26.05	25.89	25.87	25.93	24.55	24.36	24.24	24.38
34	Me-QRP-00033	31.10	30.81	30.76	30.89	25.06	24.94	24.84	24.95	28.18	28.17	28.37	28.24
35	Me-QRP-00034	33.65	33.05	33.27	33.32	32.51	32.14	32.39	32.35	0.00	0.00	0.00	0.00
36	Me-QRP-00035	0.00	0.00	0.00	0.00	0.00	0.00	0.00	0.00	36.08	38.52	38.29	37.63
37	Me-QRP-00036	0.00	0.00	0.00	0.00	0.00	0.00	0.00	0.00	0.00	0.00	0.00	0.00
38	Me-QRP-00037	32.78	32.55	33.11	32.81	31.77	31.47	31.30	31.52	30.48	30.47	30.71	30.55

39	Me-QRP-00038	31.16	31.34	30.98	31.16	29.75	29.65	29.78	29.73	29.46	29.15	28.97	29.19
40	Me-QRP-00039	0.00	0.00	0.00	0.00	0.00	0.00	0.00	0.00	0.00	0.00	0.00	0.00
41	Me-QRP-00040	34.84	34.97	37.68	35.83	34.92	34.87	35.26	35.02	36.18	36.57	37.89	36.88
42	Me-QRP-00041	29.64	29.37	29.30	29.44	22.01	21.84	21.75	21.87	24.39	24.37	24.34	24.37
43	Me-QRP-00042	32.07	31.78	31.94	31.93	31.82	31.88	31.49	31.73	31.30	31.39	31.34	31.34
44	Me-QRP-00043	27.29	27.22	27.10	27.20	25.43	26.13	25.48	25.68	25.67	25.30	25.25	25.41
45	Me-QRP-00044	0.00	0.00	0.00	0.00	0.00	0.00	0.00	0.00	0.00	0.00	0.00	0.00
46	Me-QRP-00045	27.76	27.54	27.54	27.62	25.89	25.71	25.58	25.73	25.35	25.21	25.12	25.23
47	Me-QRP-00046	30.59	30.36	30.38	30.44	30.24	30.52	30.72	30.49	28.93	28.94	29.13	29.00
48	Me-QRP-00047	27.13	27.14	27.01	27.09	26.81	26.64	26.49	26.65	27.70	27.71	27.80	27.74
49	Me-QRP-00048	31.31	30.95	31.31	31.19	34.78	34.91	34.25	34.65	31.07	31.61	31.00	31.23
50	Me-QRP-00049	31.61	30.41	31.06	31.03	29.94	29.65	29.14	29.57	23.56	24.82	24.18	24.19
51	Me-QRP-00050	30.60	30.18	30.60	30.46	35.62	37.68	37.78	37.02	30.74	30.69	30.40	30.61
52	Me-QRP-00051	0.00	0.00	0.00	0.00	0.00	0.00	0.00	0.00	35.12	0.00	36.42	35.77
53	Me-QRP-00052	28.75	28.56	28.61	28.64	27.33	27.02	27.19	27.18	27.20	27.08	27.16	27.15
54	Me-QRP-00053	25.94	25.69	25.64	25.75	22.92	22.77	22.72	22.80	28.49	28.40	28.43	28.44
55	Me-QRP-00054	25.40	25.36	25.41	25.39	26.48	26.37	26.44	26.43	28.02	27.82	27.87	27.90
56	Me-QRP-00055	25.08	25.04	24.95	25.02	26.86	26.75	26.55	26.72	29.66	29.31	29.53	29.50
57	Me-QRP-00056	29.03	29.18	29.23	29.15	29.35	29.10	29.04	29.16	29.16	29.46	29.08	29.23
58	Me-QRP-00053	25.94	25.69	25.64	25.75	22.92	22.77	22.72	22.80	28.49	28.40	28.43	28.44
59	Me-QRP-00058	23.85	23.00	23.55	23.47	26.27	25.96	25.13	25.79	25.42	25.31	25.07	25.27
60	Me-QRP-00004	32.59	32.34	32.14	32.36	26.12	25.92	25.84	25.96	25.01	24.85	24.92	24.92
61	Me-QRP-00060	26.22	26.08	26.14	26.15	27.24	27.00	26.93	27.05	26.31	26.43	26.35	26.36
62	Me-QRP-00061	23.81	23.70	23.62	23.71	24.24	23.93	23.88	24.02	26.19	26.01	25.84	26.01
63	Me-QRP-00062	0.00	0.00	0.00	0.00	0.00	0.00	0.00	0.00	0.00	0.00	0.00	0.00
64	Me-QRP-00063	0.00	0.00	0.00	0.00	0.00	0.00	0.00	0.00	36.16	37.01	36.05	36.41
65	Me-QRP-00064	30.14	30.33	30.56	30.34	27.07	27.05	27.09	27.07	27.46	27.55	27.59	27.53
66	Me-QRP-00065	28.98	28.82	28.78	28.86	27.92	27.86	27.85	27.88	27.87	27.81	28.01	27.90
67	Me-QRP-00066	0.00	0.00	0.00	0.00	0.00	0.00	0.00	0.00	0.00	0.00	37.60	0.00
68	Me-QRP-00067	0.00	0.00	0.00	0.00	0.00	0.00	0.00	0.00	37.51	38.53	0.00	0.00
69	Me-QRP-00068	0.00	0.00	0.00	0.00	0.00	0.00	0.00	0.00	0.00	0.00	37.86	0.00
70	Me-QRP-00008	28.07	27.93	27.93	27.97	26.51	26.32	26.11	26.32	23.70	23.46	23.57	23.58
71	Me-QRP-00070	0.00	0.00	0.00	0.00	0.00	0.00	0.00	0.00	0.00	0.00	0.00	0.00
72	Me-QRP-00071	28.03	27.95	27.98	27.99	23.83	23.74	23.61	23.73	24.10	23.93	24.01	24.02
73	Me-QRP-00072	0.00	0.00	0.00	0.00	0.00	0.00	0.00	0.00	38.12	0.00	0.00	0.00
74	Me-QRP-00073	28.29	27.96	28.09	28.11	26.31	26.18	26.16	26.21	27.62	27.58	27.46	27.55
75	Me-QRP-00074	30.04	30.04	29.95	30.01	29.86	29.81	29.83	29.84	27.34	26.93	26.99	27.09
76	Me-QRP-00075	24.75	24.55	24.58	24.63	23.21	23.14	23.16	23.17	22.71	22.67	22.57	22.65
77	Me-QRP-00076	28.13	28.05	27.98	28.05	25.98	25.95	26.05	25.99	27.65	27.70	27.77	27.71
78	Me-QRP-00077	27.76	27.60	27.62	27.66	25.88	25.67	25.58	25.71	26.41	26.38	26.24	26.34
79	Me-QRP-00078	28.65	28.51	28.70	28.62	25.90	25.83	25.65	25.79	28.95	28.45	28.54	28.65
80	Me-QRP-00079	28.65	28.51	28.70	28.62	25.90	25.83	25.65	25.79	28.41	28.27	28.57	28.42
81	Me-QRP-00080	32.88	30.05	32.88	31.93	30.92	30.77	31.02	30.90	29.85	29.73	29.48	29.69

82	Me-QRP-00081	27.77	28.28	29.43	28.49	27.31	26.73	27.36	27.14	27.31	27.48	27.43	27.41
83	Me-QRP-00082	28.83	28.73	28.85	28.81	26.92	26.83	27.00	26.92	27.60	27.56	27.79	27.65
84	Me-QRP-00083	29.25	29.11	29.11	29.16	25.73	25.44	25.61	25.59	28.20	28.03	28.09	28.10
85	Me-QRP-00084	26.02	26.00	25.98	26.00	26.52	26.38	26.42	26.44	25.64	25.70	25.53	25.63
86	Me-QRP-00085	26.51	26.42	26.42	26.45	25.10	25.09	25.12	25.11	24.55	24.41	24.35	24.43
87	Me-QRP-00086	27.57	27.51	27.51	27.53	24.74	24.80	24.80	24.78	26.24	26.20	26.30	26.25
88	Me-QRP-00075	24.75	24.55	24.58	24.63	23.21	23.14	23.16	23.17	24.98	24.87	24.99	24.95
89	Me-QRP-00088	27.37	27.44	27.34	27.38	24.23	24.11	24.29	24.21	25.19	24.99	25.16	25.11
90	Me-QRP-00089	28.41	28.37	28.41	28.40	25.78	25.66	25.72	25.72	31.17	30.96	31.09	31.07
91	Me-QRP-00090	31.14	30.97	31.01	31.04	30.03	29.98	29.82	29.94	22.71	22.67	22.57	22.65
92	Me-QRP-00091	25.77	25.65	25.67	25.70	27.81	27.82	27.78	27.80	27.80	27.36	27.26	27.47
93	Me-QRP-00092	27.15	27.35	27.20	27.24	26.43	26.21	26.35	26.33	24.93	24.93	24.94	24.93
94	Me-QRP-00093	27.51	27.40	27.33	27.41	26.23	26.11	26.15	26.16	25.30	25.22	25.20	25.24
95	Me-QRP-00094	28.69	28.57	28.69	28.65	27.63	27.49	27.52	27.55	25.73	25.66	25.67	25.69
96	Me-QRP-00095	27.87	27.78	27.70	27.78	26.65	26.53	26.66	26.61	25.01	24.86	24.79	24.89
97	Me-QRP-00096	26.60	26.54	26.53	26.56	27.09	27.18	27.19	27.15	26.01	25.99	25.89	25.96
98	Me-QRP-00097	0.00	0.00	0.00	0.00	0.00	0.00	0.00	0.00	0.00	0.00	0.00	0.00
99	Me-QRP-00098	28.51	28.39	28.45	28.45	26.01	25.83	25.72	25.85	24.16	24.14	24.21	24.17
100	Me-QRP-00099	28.95	28.95	28.82	28.91	30.17	30.05	30.30	30.17	26.73	26.59	26.73	26.68
101	Me-QRP-00100	0.00	0.00	0.00	0.00	0.00	0.00	0.00	0.00	34.06	32.77	33.16	33.33
102	Me-QRP-00101	33.03	32.98	33.03	33.01	31.31	31.29	31.41	31.34	30.84	30.86	30.34	30.68
103	Me-QRP-00102	30.27	30.13	30.04	30.15	26.91	26.86	26.77	26.85	26.62	26.71	26.55	26.62
104	Me-QRP-00103	30.30	30.09	30.00	30.13	28.31	28.35	28.45	28.37	28.66	28.47	28.55	28.56
105	Me-QRP-00104	27.67	27.42	27.40	27.50	26.00	25.79	25.82	25.87	24.17	23.97	23.99	24.04
106	Me-QRP-00105	27.82	27.68	27.70	27.74	26.43	26.46	26.54	26.48	25.08	24.89	24.95	24.97
107	Me-QRP-00106	29.14	29.16	29.33	29.21	27.05	27.06	27.10	27.07	28.19	28.06	28.07	28.11

Supplementary Table S5 qPCR cycles of the genes in H124 at formation of root system													
	Sample	H124 leaves				H124 stems				H124 roots			
	primer ID	Ct1	Ct2	Ct3	Ave.Ct	Ct1	Ct2	Ct3	Ave.Ct	Ct1	Ct2	Ct3	Ave.Ct
1	Me-QRP-90001	23.13	23.01	22.92	23.02	21.07	21.00	20.92	21.00	22.65	22.56	22.35	22.52
2	Me-QRP-00001	24.70	24.72	24.43	24.62	22.79	22.12	22.10	22.34	24.47	24.33	24.17	24.32
3	Me-QRP-00002	26.63	26.39	26.30	26.44	22.51	22.31	22.27	22.37	24.13	24.01	23.92	24.02
4	Me-QRP-00003	0.00	0.00	0.00	0.00	0.00	0.00	0.00	0.00	0.00	0.00	0.00	0.00
5	Me-QRP-00004	28.44	28.24	28.08	28.25	23.69	23.73	23.38	23.60	24.44	24.36	24.25	24.35
6	Me-QRP-00005	24.49	24.30	24.12	24.30	23.84	23.55	23.56	23.65	23.41	23.26	22.99	23.22
7	Me-QRP-00006	27.04	26.90	26.86	26.93	25.50	25.58	25.13	25.40	26.79	26.37	26.23	26.46
8	Me-QRP-00007	28.25	28.16	28.05	28.16	26.75	26.63	26.56	26.65	26.85	26.82	26.78	26.81
9	Me-QRP-00008	26.90	26.95	26.81	26.89	25.36	25.23	25.08	25.22	26.88	26.45	26.31	26.54
10	Me-QRP-00009	26.96	26.92	26.89	26.93	26.76	26.78	26.85	26.80	29.85	29.99	29.91	29.92
11	Me-QRP-00010	31.59	31.50	31.30	31.46	31.75	31.40	31.33	31.49	30.81	30.71	30.53	30.68
12	Me-QRP-00011	25.87	25.91	25.91	25.90	25.43	25.43	25.18	25.35	24.16	24.17	24.11	24.15
13	Me-QRP-00012	23.22	23.35	23.25	23.27	25.33	25.26	24.92	25.17	27.61	27.55	27.27	27.48
14	Me-QRP-00013	28.92	28.46	28.77	28.72	25.81	25.70	25.67	25.73	23.84	23.80	23.66	23.77
15	Me-QRP-00014	24.91	24.79	24.92	24.87	22.56	22.73	22.45	22.58	22.64	22.50	22.35	22.50
16	Me-QRP-00015	24.59	24.42	24.47	24.49	22.31	22.14	22.16	22.21	23.78	23.71	23.60	23.70
17	Me-QRP-00016	26.72	26.73	26.70	26.72	24.42	24.10	24.08	24.20	24.00	24.05	23.99	24.01
18	Me-QRP-00017	23.22	23.03	22.91	23.06	21.57	21.45	21.07	21.36	22.73	22.47	22.25	22.48
19	Me-QRP-00018	26.56	26.46	26.22	26.41	24.21	23.89	23.74	23.94	23.25	23.29	23.29	23.28
20	Me-QRP-00019	29.51	29.25	29.21	29.32	27.52	27.24	27.07	27.28	25.99	25.97	25.85	25.94
21	Me-QRP-00020	26.87	26.87	26.87	26.87	26.12	25.83	25.73	25.89	26.90	26.88	26.85	26.88
22	Me-QRP-00021	29.74	29.91	29.51	29.72	31.20	30.67	30.85	30.91	29.14	29.12	28.93	29.06
23	Me-QRP-00022	23.09	23.18	23.09	23.12	25.60	25.32	25.10	25.34	23.02	22.97	22.83	22.94
24	Me-QRP-00023	26.84	26.75	26.72	26.77	25.88	25.69	25.52	25.70	26.36	26.64	26.20	26.40
25	Me-QRP-00024	26.96	26.94	26.92	26.94	25.31	25.15	25.07	25.18	26.10	26.09	25.97	26.05
26	Me-QRP-00025	35.72	35.09	34.40	35.07	32.28	32.29	32.21	32.26	33.32	33.04	32.45	32.94
27	Me-QRP-00026	28.05	27.92	28.19	28.05	26.62	26.69	26.35	26.55	27.06	26.78	26.59	26.81
28	Me-QRP-00027	25.71	25.63	25.74	25.69	24.09	24.20	23.99	24.09	25.75	25.62	25.54	25.64
29	Me-QRP-00028	32.82	33.01	32.52	32.78	34.82	34.13	34.03	34.33	34.96	35.24	35.04	35.08
30	Me-QRP-00029	0.00	0.00	0.00	0.00	0.00	0.00	0.00	0.00	0.00	0.00	0.00	0.00
31	Me-QRP-00030	28.55	28.50	28.44	28.50	29.86	29.76	29.54	29.72	29.08	29.14	28.98	29.06
32	Me-QRP-00031	29.02	28.85	28.89	28.92	27.71	27.53	27.40	27.54	29.49	29.47	29.26	29.41
33	Me-QRP-00032	27.44	27.34	27.28	27.35	25.94	25.83	25.72	25.83	25.40	25.49	25.39	25.43
34	Me-QRP-00033	27.74	27.88	27.72	27.78	24.59	24.59	24.31	24.50	24.46	24.43	24.33	24.41
35	Me-QRP-00034	32.71	32.18	32.20	32.36	32.37	32.03	31.79	32.06	31.43	31.24	31.04	31.24
36	Me-QRP-00035	0.00	0.00	0.00	0.00	0.00	0.00	0.00	0.00	0.00	0.00	0.00	0.00
37	Me-QRP-00036	0.00	0.00	0.00	0.00	0.00	0.00	0.00	0.00	0.00	0.00	0.00	0.00
38	Me-QRP-00037	31.39	31.67	31.10	31.38	32.76	32.49	32.77	32.67	31.10	31.06	30.86	31.01
39	Me-QRP-00038	29.18	29.08	29.20	29.16	28.74	28.32	28.25	28.44	29.56	29.60	29.52	29.56

40	Me-QRP-00039	0.00	0.00	0.00	0.00	0.00	0.00	0.00	0.00	0.00	0.00	0.00	0.00
41	Me-QRP-00040	36.58	34.95	36.20	35.91	34.57	34.87	34.36	34.60	35.69	36.08	35.50	35.75
42	Me-QRP-00041	26.85	26.87	26.74	26.82	24.26	24.43	24.10	24.26	23.92	23.78	23.66	23.79
43	Me-QRP-00042	31.72	31.86	31.44	31.67	31.20	31.23	31.18	31.20	31.49	31.23	31.14	31.29
44	Me-QRP-00043	26.29	26.18	26.29	26.25	24.00	23.88	23.85	23.91	25.02	24.94	24.84	24.94
45	Me-QRP-00044	0.00	0.00	0.00	0.00	0.00	0.00	0.00	0.00	0.00	0.00	0.00	0.00
46	Me-QRP-00045	26.75	26.63	26.40	26.59	26.20	26.12	25.98	26.10	26.53	26.36	26.26	26.39
47	Me-QRP-00046	29.34	29.18	28.97	29.16	31.52	31.78	31.99	31.76	31.21	30.91	30.96	31.03
48	Me-QRP-00047	27.33	27.23	27.07	27.21	28.12	28.00	27.90	28.01	26.95	26.87	26.73	26.85
49	Me-QRP-00048	30.40	30.36	30.34	30.37	37.74	36.12	N/A	36.93	35.43	36.56	35.57	35.86
50	Me-QRP-00049	30.43	30.17	30.35	30.32	28.24	28.26	27.79	28.10	29.13	28.92	28.77	28.94
51	Me-QRP-00050	29.13	29.13	29.06	29.11	31.37	31.36	31.38	31.37	31.24	31.48	31.24	31.32
52	Me-QRP-00051	0.00	0.00	0.00	0.00	0.00	0.00	0.00	0.00	0.00	0.00	0.00	0.00
53	Me-QRP-00052	27.91	27.69	27.67	27.76	26.05	25.93	25.89	25.96	26.49	26.24	26.11	26.28
54	Me-QRP-00053	25.42	25.27	25.17	25.29	23.05	22.98	22.87	22.97	23.92	23.95	23.79	23.89
55	Me-QRP-00054	25.20	25.04	25.21	25.15	26.34	26.51	26.23	26.36	27.51	27.24	27.00	27.25
56	Me-QRP-00055	25.23	24.98	24.81	25.01	26.39	26.28	26.15	26.28	27.67	27.35	27.21	27.41
57	Me-QRP-00056	28.24	28.41	28.15	28.27	27.94	27.95	27.92	27.93	28.60	28.35	28.32	28.43
58	Me-QRP-00053	25.42	25.27	25.17	25.29	23.05	22.98	22.87	22.97	23.92	23.95	23.79	23.89
59	Me-QRP-00058	22.19	22.23	21.84	22.09	24.11	23.78	23.27	23.72	20.49	20.60	19.80	20.30
60	Me-QRP-00004	28.44	28.24	28.08	28.25	23.69	23.73	23.38	23.60	24.44	24.36	24.25	24.35
61	Me-QRP-00060	27.14	26.97	26.81	26.98	26.81	26.60	26.42	26.61	27.04	26.88	26.80	26.91
62	Me-QRP-00061	22.39	22.24	22.14	22.26	22.39	22.36	22.11	22.29	25.35	25.12	24.88	25.12
63	Me-QRP-00062	0.00	0.00	0.00	0.00	0.00	0.00	0.00	0.00	0.00	0.00	0.00	0.00
64	Me-QRP-00063	0.00	0.00	0.00	0.00	0.00	0.00	0.00	0.00	0.00	0.00	0.00	0.00
65	Me-QRP-00064	28.82	28.54	28.83	28.73	30.58	30.74	30.46	30.59	28.94	28.95	28.79	28.89
66	Me-QRP-00065	28.93	28.59	28.90	28.81	29.20	29.19	29.06	29.15	27.49	27.27	27.05	27.27
67	Me-QRP-00066	0.00	0.00	0.00	0.00	0.00	0.00	0.00	0.00	0.00	0.00	0.00	0.00
68	Me-QRP-00067	0.00	0.00	0.00	0.00	0.00	0.00	0.00	0.00	0.00	0.00	0.00	0.00
69	Me-QRP-00068	0.00	0.00	0.00	0.00	0.00	0.00	0.00	0.00	0.00	0.00	0.00	0.00
70	Me-QRP-00008	26.90	26.95	26.81	26.89	25.36	25.23	25.08	25.22	26.88	26.45	26.31	26.54
71	Me-QRP-00070	0.00	0.00	0.00	0.00	0.00	0.00	0.00	0.00	0.00	0.00	0.00	0.00
72	Me-QRP-00071	25.46	25.34	25.31	25.37	22.15	22.08	21.95	22.06	23.79	23.60	23.49	23.63
73	Me-QRP-00072	0.00	0.00	0.00	0.00	0.00	0.00	0.00	0.00	0.00	0.00	0.00	0.00
74	Me-QRP-00073	26.24	26.06	26.18	26.16	24.72	24.92	24.33	24.66	25.64	25.37	25.20	25.40
75	Me-QRP-00074	28.42	28.10	28.35	28.29	27.66	27.48	27.43	27.52	29.66	29.47	29.40	29.51
76	Me-QRP-00075	24.80	24.62	24.75	24.72	24.27	24.08	24.10	24.15	23.33	23.22	23.09	23.22
77	Me-QRP-00076	26.49	26.41	26.45	26.45	26.08	26.05	26.01	26.05	26.37	26.33	26.25	26.32
78	Me-QRP-00077	25.93	25.88	25.77	25.86	23.89	23.89	23.81	23.86	25.98	25.86	25.71	25.85
79	Me-QRP-00078	27.61	27.52	27.26	27.46	25.31	25.33	25.23	25.29	25.66	25.52	25.43	25.54
80	Me-QRP-00079	27.61	27.52	27.26	27.46	25.31	25.33	25.23	25.29	25.66	25.52	25.43	25.54
81	Me-QRP-00080	31.67	31.86	31.78	31.77	28.47	28.80	28.43	28.57	30.49	30.33	30.62	30.48
82	Me-QRP-00081	28.43	28.58	28.60	28.54	27.21	27.26	26.86	27.11	28.51	27.91	27.74	28.05

83	Me-QRP-00082	27.33	27.29	27.27	27.30	27.23	27.21	27.14	27.19	27.01	26.96	26.81	26.93
84	Me-QRP-00083	27.21	27.12	27.28	27.20	24.16	24.32	24.14	24.21	25.30	25.08	24.91	25.10
85	Me-QRP-00084	25.56	25.42	25.54	25.51	24.90	25.19	24.92	25.00	27.07	26.89	26.85	26.94
86	Me-QRP-00085	25.42	25.38	25.48	25.43	24.44	24.75	24.45	24.55	25.21	25.10	24.89	25.07
87	Me-QRP-00086	25.84	25.73	25.77	25.78	23.16	22.96	22.96	23.03	23.68	23.51	23.35	23.51
88	Me-QRP-00075	24.80	24.62	24.75	24.72	24.27	24.08	24.10	24.15	23.33	23.22	23.09	23.22
89	Me-QRP-00088	25.72	25.55	25.70	25.66	24.73	24.86	24.71	24.77	23.53	23.37	23.09	23.33
90	Me-QRP-00089	26.83	26.80	26.90	26.84	24.37	24.68	24.45	24.50	28.76	28.78	28.62	28.72
91	Me-QRP-00090	29.69	29.63	29.76	29.69	28.70	28.72	28.62	28.68	29.49	29.94	29.52	29.65
92	Me-QRP-00091	25.94	25.72	25.80	25.82	28.44	28.34	28.23	28.34	25.29	25.21	25.07	25.19
93	Me-QRP-00092	25.80	25.72	25.82	25.78	24.63	24.80	24.50	24.64	26.65	26.36	26.09	26.37
94	Me-QRP-00093	26.04	25.93	26.09	26.02	24.81	25.18	24.82	24.93	26.12	26.04	25.90	26.02
95	Me-QRP-00094	28.26	28.05	28.16	28.16	26.00	26.22	25.95	26.06	27.81	27.63	27.35	27.60
96	Me-QRP-00095	26.58	26.29	26.46	26.45	25.58	25.45	25.34	25.45	27.12	26.96	26.87	26.98
97	Me-QRP-00096	25.57	25.37	25.41	25.45	26.01	25.93	25.80	25.91	27.12	27.01	26.97	27.04
98	Me-QRP-00097	0.00	0.00	0.00	0.00	0.00	0.00	0.00	0.00	0.00	0.00	0.00	0.00
99	Me-QRP-00098	26.47	26.33	26.34	26.38	24.75	24.59	24.61	24.65	24.91	24.74	24.64	24.76
100	Me-QRP-00099	27.37	27.49	27.36	27.41	28.04	27.95	27.83	27.94	25.79	25.59	25.60	25.66
101	Me-QRP-00100	0.00	0.00	0.00	0.00	0.00	0.00	0.00	0.00	0.00	0.00	0.00	0.00
102	Me-QRP-00101	33.29	33.26	33.71	33.42	32.36	32.82	32.40	32.53	32.97	32.77	32.39	32.71
103	Me-QRP-00102	28.66	28.43	28.45	28.52	25.24	25.44	25.20	25.29	26.40	26.32	26.81	26.51
104	Me-QRP-00103	28.85	28.75	28.82	28.81	27.93	27.75	27.67	27.78	27.90	27.88	27.45	27.74
105	Me-QRP-00104	24.37	24.12	24.21	24.23	24.29	24.32	24.01	24.20	26.39	25.88	25.71	26.00
106	Me-QRP-00105	26.50	26.35	26.33	26.39	25.14	25.01	24.98	25.05	26.75	26.83	26.60	26.73
107	Me-QRP-00106	27.85	27.75	27.86	27.82	27.25	27.33	27.16	27.25	26.68	26.56	26.36	26.53

**Supplementary Table S6** qPCR cycles of the genes in F01 at formation of root system													
	Sample	F01 leaves				F01 stems				F01 roots			
	primer ID	Ct1	Ct2	Ct3	Ave.Ct	Ct1	Ct2	Ct3	Ave.Ct	Ct1	Ct2	Ct3	Ave.Ct
1	Me-QRP-90001	22.97	22.70	22.71	22.79	22.47	22.45	22.22	22.38	21.86	21.73	21.78	21.79
2	Me-QRP-00001	24.71	24.37	24.46	24.51	23.73	23.38	23.31	23.47	23.19	23.16	23.18	23.18
3	Me-QRP-00002	25.84	25.49	25.38	25.57	22.96	22.90	22.90	22.92	22.90	22.81	22.85	22.85
4	Me-QRP-00003	0.00	0.00	0.00	0.00	0.00	0.00	0.00	0.00	0.00	0.00	0.00	0.00
5	Me-QRP-00004	27.17	26.81	26.78	26.92	25.07	24.97	25.01	25.01	23.47	23.38	23.49	23.45
6	Me-QRP-00005	25.08	24.61	24.48	24.73	23.98	23.79	23.66	23.81	23.98	23.75	23.92	23.88
7	Me-QRP-00006	27.38	27.12	27.05	27.19	26.58	26.09	26.12	26.26	25.96	25.97	25.89	25.94
8	Me-QRP-00007	27.86	27.47	27.53	27.62	25.92	25.95	25.82	25.89	26.86	26.87	26.70	26.81
9	Me-QRP-00008	27.07	26.80	26.79	26.89	25.74	25.65	25.53	25.64	25.77	25.72	25.77	25.75
10	Me-QRP-00009	27.07	27.33	27.26	27.22	27.94	28.13	28.25	28.10	28.01	27.78	27.66	27.82
11	Me-QRP-00010	31.62	30.80	30.86	31.09	30.62	30.75	30.66	30.68	30.24	30.27	30.32	30.28
12	Me-QRP-00011	26.10	25.95	25.87	25.97	25.42	25.31	25.35	25.36	23.79	23.78	23.81	23.79
13	Me-QRP-00012	23.84	23.58	23.47	23.63	25.12	25.08	25.29	25.17	27.19	26.92	27.09	27.07
14	Me-QRP-00013	28.94	28.65	28.34	28.64	24.43	24.34	24.37	24.38	25.21	24.83	24.86	24.97
15	Me-QRP-00014	24.73	24.74	24.61	24.69	22.92	22.94	22.86	22.91	23.00	23.04	23.12	23.05
16	Me-QRP-00015	24.90	24.55	24.44	24.63	23.54	23.53	23.39	23.49	24.03	23.89	23.90	23.94
17	Me-QRP-00016	26.47	26.10	26.20	26.26	23.33	23.17	23.21	23.23	24.71	24.62	24.59	24.64
18	Me-QRP-00017	23.61	23.00	23.43	23.35	22.74	22.52	22.72	22.66	22.80	22.70	22.77	22.76
19	Me-QRP-00018	26.24	25.78	25.78	25.93	23.44	23.27	23.30	23.34	23.74	23.70	23.66	23.70
20	Me-QRP-00019	28.89	28.58	28.71	28.72	29.48	29.27	29.25	29.33	27.61	27.58	27.40	27.53
21	Me-QRP-00020	26.85	26.69	26.53	26.69	26.22	26.00	26.08	26.10	26.52	26.51	26.58	26.54
22	Me-QRP-00021	30.73	30.25	30.55	30.51	31.00	30.55	30.81	30.79	30.15	30.16	30.18	30.17
23	Me-QRP-00022	23.70	23.29	23.44	23.48	24.60	24.45	24.58	24.54	23.85	23.85	23.87	23.86
24	Me-QRP-00023	26.83	26.49	26.48	26.60	25.31	25.11	25.19	25.20	25.76	25.80	25.73	25.76
25	Me-QRP-00024	26.51	26.08	26.13	26.24	25.39	25.22	25.17	25.26	25.74	25.65	25.54	25.64
26	Me-QRP-00025	34.71	34.68	34.30	34.56	34.22	34.14	34.29	34.22	32.05	32.39	32.49	32.31
27	Me-QRP-00026	28.53	28.48	28.35	28.45	26.99	26.93	26.69	26.87	26.91	26.93	26.98	26.94
28	Me-QRP-00027	25.47	25.34	25.37	25.39	25.67	25.82	25.73	25.74	26.02	25.93	26.13	26.03
29	Me-QRP-00028	32.41	32.12	32.17	32.24	33.36	33.60	33.27	33.41	34.58	34.20	34.93	34.57
30	Me-QRP-00029	0.00	0.00	0.00	0.00	0.00	0.00	0.00	0.00	0.00	0.00	0.00	0.00
31	Me-QRP-00030	29.01	28.80	28.81	28.87	29.24	29.05	29.15	29.15	29.42	29.63	29.42	29.49
32	Me-QRP-00031	29.54	29.28	29.12	29.31	27.98	27.99	27.98	27.98	28.89	28.80	28.85	28.85
33	Me-QRP-00032	27.14	26.79	26.77	26.90	25.52	25.38	25.42	25.44	25.36	25.28	25.18	25.27
34	Me-QRP-00033	27.20	26.95	27.05	27.07	24.88	24.68	25.00	24.85	25.57	25.47	25.62	25.56
35	Me-QRP-00034	32.96	32.60	32.65	32.74	32.08	31.59	31.76	31.81	30.87	30.90	30.60	30.79
36	Me-QRP-00035	0.00	0.00	0.00	0.00	0.00	0.00	0.00	0.00	0.00	0.00	0.00	0.00
37	Me-QRP-00036	0.00	0.00	0.00	0.00	0.00	0.00	0.00	0.00	0.00	0.00	0.00	0.00
38	Me-QRP-00037	32.16	31.48	31.68	31.77	30.72	30.60	30.55	30.62	31.14	31.15	31.31	31.20
39	Me-QRP-00038	29.90	29.66	29.71	29.75	27.64	27.60	27.60	27.61	28.96	28.90	28.98	28.95

40	Me-QRP-00039	0.00	0.00	0.00	0.00	0.00	0.00	0.00	0.00	0.00	0.00	0.00	0.00
41	Me-QRP-00040	34.56	34.65	35.47	34.89	35.54	35.76	33.46	34.92	35.81	36.81	35.83	36.15
42	Me-QRP-00041	26.96	26.60	26.66	26.74	25.09	24.85	24.90	24.95	22.85	22.78	22.79	22.81
43	Me-QRP-00042	31.79	31.44	31.26	31.50	30.59	30.52	30.59	30.57	31.48	31.59	31.41	31.49
44	Me-QRP-00043	26.10	25.70	25.57	25.79	23.89	23.79	23.90	23.86	24.74	24.67	24.70	24.70
45	Me-QRP-00044	0.00	0.00	0.00	0.00	0.00	0.00	0.00	0.00	0.00	0.00	0.00	0.00
46	Me-QRP-00045	27.05	26.62	26.66	26.78	25.54	25.39	25.39	25.44	26.78	26.78	26.61	26.72
47	Me-QRP-00046	30.30	29.83	29.72	29.95	30.21	30.12	29.88	30.07	32.10	31.86	31.68	31.88
48	Me-QRP-00047	27.12	26.93	26.66	26.90	25.30	25.13	25.22	25.22	27.69	27.72	27.60	27.67
49	Me-QRP-00048	31.28	30.58	30.44	30.77	32.53	32.52	32.78	32.61	34.37	34.84	34.09	34.43
50	Me-QRP-00049	30.19	29.41	29.75	29.78	29.59	28.90	29.34	29.28	27.94	28.26	28.14	28.12
51	Me-QRP-00050	30.71	30.45	30.31	30.49	33.03	33.17	32.88	33.03	30.76	31.00	30.78	30.85
52	Me-QRP-00051	0.00	0.00	0.00	0.00	0.00	0.00	0.00	0.00	0.00	0.00	0.00	0.00
53	Me-QRP-00052	28.18	27.78	27.69	27.88	26.58	26.34	26.30	26.41	25.71	25.65	25.58	25.65
54	Me-QRP-00053	25.03	24.86	24.72	24.87	22.76	22.49	22.54	22.60	24.12	24.02	24.15	24.10
55	Me-QRP-00054	25.19	25.08	25.11	25.12	25.69	25.78	25.80	25.76	26.69	26.57	26.75	26.67
56	Me-QRP-00055	25.06	24.59	24.81	24.82	26.03	25.76	25.94	25.91	26.67	26.76	26.73	26.72
57	Me-QRP-00056	28.84	28.43	28.55	28.61	28.15	28.01	28.03	28.06	27.86	27.75	27.71	27.77
58	Me-QRP-00053	25.03	24.86	24.72	24.87	22.76	22.49	22.54	22.60	24.12	24.02	24.15	24.10
59	Me-QRP-00058	21.26	21.13	21.17	21.19	24.52	23.57	24.08	24.05	22.71	22.54	23.00	22.75
60	Me-QRP-00004	27.17	26.81	26.78	26.92	25.07	24.97	25.01	25.01	23.47	23.38	23.49	23.45
61	Me-QRP-00060	26.50	26.11	26.13	26.25	25.76	25.43	25.52	25.57	27.45	27.33	27.18	27.32
62	Me-QRP-00061	22.89	22.47	22.49	22.62	24.34	24.05	24.05	24.15	24.75	24.74	24.76	24.75
63	Me-QRP-00062	0.00	0.00	0.00	0.00	0.00	0.00	0.00	0.00	0.00	0.00	0.00	0.00
64	Me-QRP-00063	0.00	0.00	0.00	0.00	0.00	0.00	0.00	0.00	0.00	0.00	0.00	0.00
65	Me-QRP-00064	29.62	29.55	29.53	29.57	27.99	28.06	28.16	28.07	28.81	28.88	28.79	28.83
66	Me-QRP-00065	29.44	29.32	29.45	29.40	27.84	27.85	27.76	27.82	28.27	28.15	28.12	28.18
67	Me-QRP-00066	0.00	0.00	0.00	0.00	0.00	0.00	0.00	0.00	0.00	0.00	0.00	0.00
68	Me-QRP-00067	0.00	0.00	0.00	0.00	0.00	0.00	0.00	0.00	0.00	0.00	0.00	0.00
69	Me-QRP-00068	0.00	0.00	0.00	0.00	0.00	0.00	0.00	0.00	0.00	0.00	0.00	0.00
70	Me-QRP-00008	27.07	26.80	26.79	26.89	25.74	25.65	25.53	25.64	25.77	25.72	25.77	25.75
71	Me-QRP-00070	0.00	0.00	0.00	0.00	0.00	0.00	0.00	0.00	0.00	0.00	0.00	0.00
72	Me-QRP-00071	25.07	24.85	24.91	24.95	23.33	22.91	23.17	23.14	23.79	23.74	23.75	23.76
73	Me-QRP-00072	0.00	0.00	0.00	0.00	0.00	0.00	0.00	0.00	0.00	0.00	0.00	0.00
74	Me-QRP-00073	26.31	26.08	26.05	26.15	25.13	24.84	24.87	24.94	25.03	24.98	25.07	25.02
75	Me-QRP-00074	29.13	28.80	28.81	28.91	28.49	28.44	28.44	28.46	28.96	28.63	28.86	28.82
76	Me-QRP-00075	24.85	24.51	24.47	24.61	23.11	23.18	23.14	23.14	23.04	22.93	22.91	22.96
77	Me-QRP-00076	27.12	26.91	26.82	26.95	26.19	26.12	26.24	26.18	27.12	27.16	27.22	27.17
78	Me-QRP-00077	25.51	25.21	25.31	25.34	24.20	23.96	23.99	24.05	25.42	25.55	25.50	25.49
79	Me-QRP-00078	28.26	27.81	27.76	27.95	27.01	26.79	26.93	26.91	27.82	27.68	27.62	27.70
80	Me-QRP-00079	28.26	27.81	27.76	27.95	27.01	26.79	26.93	26.91	27.82	27.68	27.62	27.70
81	Me-QRP-00080	31.72	31.69	31.59	31.67	30.41	30.39	30.39	30.40	29.84	30.00	29.95	29.93
82	Me-QRP-00081	0.00	26.75	27.51	27.13	26.25	25.93	26.91	26.36	26.55	26.79	26.98	26.77

83	Me-QRP-00082	28.02	27.78	27.70	27.84	27.04	27.09	27.00	27.04	27.79	27.82	27.70	27.77
84	Me-QRP-00083	26.92	26.95	26.92	26.93	24.92	24.85	24.79	24.85	25.32	25.29	25.45	25.35
85	Me-QRP-00084	25.21	25.31	25.73	25.41	26.18	26.14	26.14	26.15	26.35	26.38	26.55	26.43
86	Me-QRP-00085	25.44	25.49	25.48	25.47	25.40	25.49	25.32	25.41	25.23	25.22	25.33	25.26
87	Me-QRP-00086	25.77	25.44	25.28	25.50	23.70	23.58	23.58	23.62	23.99	23.96	23.97	23.97
88	Me-QRP-00075	24.85	24.51	24.47	24.61	23.11	23.18	23.14	23.14	23.04	22.93	22.91	22.96
89	Me-QRP-00088	25.84	25.73	25.70	25.76	23.70	23.67	23.54	23.63	23.29	23.18	23.62	23.36
90	Me-QRP-00089	26.70	26.68	26.62	26.67	26.77	26.77	26.85	26.80	28.21	28.24	28.48	28.31
91	Me-QRP-00090	29.61	27.28	29.26	28.72	28.54	28.81	28.72	28.69	29.73	29.73	29.75	29.74
92	Me-QRP-00091	27.05	26.55	26.54	26.71	26.35	26.30	26.33	26.32	25.23	25.20	25.24	25.22
93	Me-QRP-00092	25.88	25.82	25.73	25.81	26.70	26.35	26.24	26.43	25.61	25.49	25.53	25.54
94	Me-QRP-00093	25.97	26.06	26.07	26.03	26.09	25.98	25.93	26.00	25.20	25.18	25.31	25.23
95	Me-QRP-00094	28.20	28.05	28.08	28.11	28.56	28.28	28.12	28.32	26.95	26.97	27.10	27.00
96	Me-QRP-00095	26.79	26.47	26.46	26.57	25.81	25.87	25.81	25.83	26.94	26.88	26.83	26.89
97	Me-QRP-00096	26.09	25.73	25.57	25.80	26.11	26.05	26.04	26.07	26.18	26.11	26.15	26.15
98	Me-QRP-00097	0.00	0.00	0.00	0.00	0.00	0.00	0.00	0.00	0.00	0.00	0.00	0.00
99	Me-QRP-00098	26.56	26.14	26.05	26.25	24.17	24.25	24.19	24.20	24.15	24.02	24.17	24.11
100	Me-QRP-00099	26.88	26.78	26.70	26.79	28.89	28.72	28.73	28.78	27.33	27.35	27.43	27.37
101	Me-QRP-00100	0.00	0.00	0.00	0.00	0.00	0.00	0.00	0.00	0.00	0.00	0.00	0.00
102	Me-QRP-00101	32.58	32.61	32.43	32.54	32.12	32.09	32.05	32.08	32.76	32.44	32.77	32.66
103	Me-QRP-00102	28.90	28.61	28.49	28.67	26.57	26.28	26.31	26.39	25.67	25.51	25.66	25.61
104	Me-QRP-00103	29.20	28.78	28.69	28.89	27.82	27.81	27.86	27.83	27.35	27.40	27.43	27.39
105	Me-QRP-00104	25.27	25.00	24.97	25.08	24.84	24.61	24.42	24.63	25.01	24.81	24.98	24.93
106	Me-QRP-00105	26.60	26.29	26.14	26.34	25.63	25.71	25.60	25.64	26.28	26.11	26.13	26.18
107	Me-QRP-00106	28.05	28.07	28.01	28.04	27.09	26.94	26.81	26.95	26.44	26.30	26.38	26.37

Supplementary Table S7 qPCR cycles of the genes in H124 at root bulking

	Sample	H124 leaves				H124 stems				H124 roots			
	primer ID	Ct1	Ct2	Ct3	Ave.Ct	Ct1	Ct2	Ct3	Ave.Ct	Ct1	Ct2	Ct3	Ave.Ct
1	Me-QRP-90001	21.45	21.74	21.60	21.60	22.27	22.02	22.10	22.13	23.19	23.08	22.97	23.08
2	Me-QRP-00001	23.93	23.96	23.94	23.94	23.77	23.68	23.72	23.72	24.59	24.69	24.65	24.64
3	Me-QRP-00002	25.96	26.12	26.01	26.03	25.01	24.98	24.90	24.96	24.95	24.94	24.90	24.93
4	Me-QRP-00003	0.00	0.00	0.00	0.00	0.00	0.00	0.00	0.00	0.00	0.00	0.00	0.00
5	Me-QRP-00004	29.31	29.66	29.21	29.40	26.39	26.07	26.21	26.22	25.33	25.25	25.27	25.28
6	Me-QRP-00005	24.04	24.08	24.01	24.04	23.62	23.48	23.53	23.54	24.46	24.27	24.29	24.34
7	Me-QRP-00006	25.71	25.88	25.63	25.74	25.00	24.82	24.91	24.91	26.70	26.71	26.74	26.71
8	Me-QRP-00007	26.09	26.18	26.13	26.13	25.86	25.71	25.70	25.76	27.77	27.70	27.58	27.68
9	Me-QRP-00008	25.55	25.70	25.60	25.62	25.40	25.23	25.39	25.34	27.01	26.96	27.03	27.00
10	Me-QRP-00009	27.04	26.86	26.79	26.90	25.98	25.63	25.93	25.85	28.88	28.64	28.45	28.66
11	Me-QRP-00010	30.76	29.73	30.76	30.42	30.74	30.61	30.68	30.68	32.05	31.73	31.37	31.72
12	Me-QRP-00011	24.47	24.63	24.43	24.51	24.48	24.17	24.36	24.34	25.30	24.79	24.71	24.93
13	Me-QRP-00012	20.54	20.77	20.47	20.59	25.26	25.06	25.21	25.18	27.45	27.35	26.96	27.25
14	Me-QRP-00013	25.90	25.90	25.90	25.90	25.97	25.70	25.63	25.77	26.30	26.23	26.15	26.23
15	Me-QRP-00014	23.46	23.44	23.40	23.43	22.14	22.18	22.20	22.18	24.11	24.16	24.34	24.20
16	Me-QRP-00015	24.80	24.82	24.83	24.82	23.98	23.72	23.69	23.80	24.34	24.29	24.25	24.29
17	Me-QRP-00016	25.25	25.29	25.26	25.27	23.43	23.20	23.21	23.28	25.72	25.26	24.93	25.30
18	Me-QRP-00017	23.18	23.75	23.30	23.41	22.20	22.02	22.20	22.14	22.96	22.69	22.74	22.80
19	Me-QRP-00018	26.88	27.02	26.98	26.96	23.68	23.33	23.40	23.47	26.36	25.61	25.19	25.72
20	Me-QRP-00019	29.22	29.35	29.28	29.28	26.33	26.26	26.10	26.23	26.78	26.37	26.16	26.43
21	Me-QRP-00020	25.79	25.90	25.73	25.81	26.01	25.80	25.90	25.90	27.79	27.37	27.13	27.43
22	Me-QRP-00021	29.70	29.80	29.27	29.59	30.64	30.10	30.37	30.37	30.45	30.18	30.14	30.26
23	Me-QRP-00022	21.78	21.99	21.75	21.84	23.99	23.85	23.89	23.91	25.44	25.03	24.92	25.13
24	Me-QRP-00023	25.43	25.54	25.41	25.46	24.82	24.79	24.53	24.71	27.66	27.33	26.89	27.29
25	Me-QRP-00024	27.47	27.66	27.55	27.56	26.61	26.38	26.32	26.44	27.40	26.97	26.74	27.04
26	Me-QRP-00025	35.53	35.78	35.04	35.45	33.72	34.10	33.98	33.94	33.73	34.03	33.39	33.72
27	Me-QRP-00026	28.07	28.11	28.10	28.09	26.86	26.93	26.90	26.90	27.63	27.65	27.47	27.58
28	Me-QRP-00027	24.99	25.11	25.11	25.07	25.41	25.58	25.56	25.52	26.49	26.50	26.39	26.46
29	Me-QRP-00028	33.17	33.74	33.30	33.40	35.87	35.06	34.85	35.26	35.07	35.16	35.99	35.41
30	Me-QRP-00029	0.00	0.00	0.00	0.00	0.00	0.00	0.00	0.00	0.00	0.00	0.00	0.00
31	Me-QRP-00030	27.82	27.87	27.78	27.82	28.82	28.13	28.26	28.40	30.14	29.86	29.54	29.85
32	Me-QRP-00031	28.01	28.00	27.97	27.99	28.90	28.62	28.60	28.71	29.91	29.79	29.45	29.71
33	Me-QRP-00032	26.20	26.14	26.15	26.16	26.07	25.81	25.77	25.89	26.25	26.04	25.86	26.05
34	Me-QRP-00033	26.88	27.05	26.80	26.91	24.04	23.83	23.94	23.94	26.99	26.78	26.74	26.84
35	Me-QRP-00034	31.97	32.12	32.10	32.06	32.36	32.25	31.65	32.09	31.81	31.74	31.57	31.70
36	Me-QRP-00035	0.00	0.00	0.00	0.00	0.00	0.00	0.00	0.00	0.00	0.00	0.00	0.00
37	Me-QRP-00036	0.00	0.00	0.00	0.00	0.00	0.00	0.00	0.00	0.00	0.00	0.00	0.00
38	Me-QRP-00037	30.76	31.16	30.58	30.83	30.74	30.56	30.24	30.51	31.71	31.43	31.45	31.53
39	Me-QRP-00038	28.03	27.93	27.99	27.98	28.02	27.78	27.64	27.81	30.03	29.78	29.63	29.81

40	Me-QRP-00039	0.00	0.00	0.00	0.00	0.00	0.00	0.00	0.00	0.00	0.00	0.00	0.00
41	Me-QRP-00040	35.67	35.40	35.46	35.51	35.81	34.58	34.90	35.10	36.59	35.94	N/A	36.26
42	Me-QRP-00041	26.38	26.55	26.33	26.42	23.76	23.39	23.53	23.56	25.81	25.77	25.62	25.73
43	Me-QRP-00042	30.02	30.17	30.16	30.12	30.70	30.52	30.37	30.53	31.44	31.20	31.18	31.27
44	Me-QRP-00043	24.48	24.50	24.43	24.47	24.34	23.75	23.73	23.94	25.99	25.92	25.93	25.94
45	Me-QRP-00044	0.00	0.00	0.00	0.00	0.00	0.00	0.00	0.00	0.00	0.00	0.00	0.00
46	Me-QRP-00045	25.54	25.63	25.52	25.56	25.66	25.46	25.42	25.51	27.38	27.41	27.18	27.33
47	Me-QRP-00046	28.44	28.52	28.35	28.44	30.25	30.44	30.55	30.41	30.84	30.87	30.85	30.85
48	Me-QRP-00047	24.91	24.96	24.86	24.91	25.37	25.20	25.34	25.30	26.81	26.72	26.20	26.58
49	Me-QRP-00048	29.87	30.01	29.79	29.89	35.14	35.31	35.79	35.41	37.30	36.42	37.43	37.05
50	Me-QRP-00049	29.08	29.70	29.17	29.32	28.53	28.19	28.18	28.30	28.96	28.49	28.64	28.69
51	Me-QRP-00050	28.27	28.29	28.12	28.23	33.69	33.47	33.71	33.62	32.59	32.93	32.76	32.76
52	Me-QRP-00051	0.00	0.00	0.00	0.00	0.00	0.00	0.00	0.00	0.00	0.00	0.00	0.00
53	Me-QRP-00052	26.58	26.74	26.58	26.63	25.67	25.51	25.52	25.56	25.67	25.57	25.50	25.58
54	Me-QRP-00053	24.24	24.30	24.32	24.29	20.13	20.37	20.27	20.26	23.02	22.92	23.01	22.98
55	Me-QRP-00054	22.79	22.97	22.88	22.88	25.42	25.58	25.57	25.52	27.76	27.69	27.67	27.71
56	Me-QRP-00055	22.69	22.86	22.66	22.74	25.56	25.76	25.92	25.75	27.64	27.76	27.69	27.70
57	Me-QRP-00056	26.29	26.47	26.24	26.33	28.16	28.12	28.02	28.10	28.18	28.02	27.95	28.05
58	Me-QRP-00053	24.24	24.30	24.32	24.29	20.13	20.37	20.27	20.26	23.02	22.92	23.01	22.98
59	Me-QRP-00058	19.88	19.74	19.91	19.84	24.36	24.41	24.16	24.31	21.99	21.63	21.70	21.78
60	Me-QRP-00004	29.31	29.66	29.21	29.40	26.39	26.07	26.21	26.22	25.33	25.25	25.27	25.28
61	Me-QRP-00060	25.15	25.18	25.08	25.14	25.57	25.42	25.34	25.44	27.31	27.15	27.14	27.20
62	Me-QRP-00061	22.82	22.53	22.35	22.56	24.22	23.95	24.11	24.10	24.83	24.91	24.89	24.88
63	Me-QRP-00062	0.00	0.00	0.00	0.00	0.00	0.00	0.00	0.00	0.00	0.00	0.00	0.00
64	Me-QRP-00063	0.00	0.00	0.00	0.00	0.00	0.00	0.00	0.00	0.00	0.00	0.00	0.00
65	Me-QRP-00064	28.15	28.20	28.14	28.16	27.96	28.03	27.98	27.99	30.15	30.09	30.05	30.10
66	Me-QRP-00065	27.06	27.19	27.24	27.16	27.11	27.24	27.05	27.13	27.96	27.88	27.77	27.87
67	Me-QRP-00066	0.00	0.00	0.00	0.00	0.00	0.00	0.00	0.00	0.00	0.00	0.00	0.00
68	Me-QRP-00067	0.00	0.00	0.00	0.00	0.00	0.00	0.00	0.00	0.00	0.00	0.00	0.00
69	Me-QRP-00068	0.00	0.00	0.00	0.00	0.00	0.00	0.00	0.00	0.00	0.00	0.00	0.00
70	Me-QRP-00008	25.55	25.70	25.60	25.62	25.40	25.23	25.39	25.34	27.01	26.96	27.03	27.00
71	Me-QRP-00070	0.00	0.00	0.00	0.00	0.00	0.00	0.00	0.00	0.00	0.00	0.00	0.00
72	Me-QRP-00071	24.96	25.12	24.93	25.00	22.94	22.78	22.82	22.85	24.46	24.38	24.52	24.45
73	Me-QRP-00072	0.00	0.00	0.00	0.00	0.00	0.00	0.00	0.00	0.00	0.00	0.00	0.00
74	Me-QRP-00073	25.73	25.78	25.82	25.78	25.13	25.25	25.11	25.16	25.68	25.64	25.73	25.68
75	Me-QRP-00074	28.04	28.00	27.90	27.98	28.89	28.74	28.60	28.74	29.74	29.59	29.69	29.68
76	Me-QRP-00075	22.31	22.20	22.14	22.22	22.37	22.11	22.10	22.19	24.53	24.43	24.43	24.46
77	Me-QRP-00076	25.43	25.59	25.42	25.48	26.50	26.19	26.13	26.27	26.84	26.75	26.75	26.78
78	Me-QRP-00077	24.74	24.99	24.73	24.82	24.74	24.58	24.74	24.69	26.14	26.19	26.25	26.20
79	Me-QRP-00078	26.21	26.23	26.19	26.21	25.17	25.12	25.02	25.10	27.11	27.06	27.07	27.08
80	Me-QRP-00079	26.21	26.23	26.19	26.21	25.17	25.12	25.02	25.10	27.11	27.06	27.07	27.08
81	Me-QRP-00080	30.05	30.29	30.23	30.19	29.20	29.48	29.37	29.35	30.06	29.92	30.02	30.00
82	Me-QRP-00081	26.79	27.39	25.84	26.67	27.31	27.03	26.65	26.99	29.00	29.36	27.13	28.50

83	Me-QRP-00082	26.31	26.38	26.36	26.35	27.28	26.90	26.98	27.05	27.24	27.16	27.04	27.15
84	Me-QRP-00083	26.50	26.67	26.53	26.57	24.71	24.61	24.61	24.64	25.95	25.85	25.88	25.89
85	Me-QRP-00084	24.66	24.83	24.74	24.74	26.01	26.14	26.15	26.10	27.33	27.33	27.33	27.33
86	Me-QRP-00085	24.69	24.81	24.79	24.76	25.18	25.29	25.38	25.29	25.29	25.34	25.26	25.30
87	Me-QRP-00086	24.91	24.93	24.87	24.90	23.59	23.18	23.08	23.28	25.41	25.37	25.31	25.36
88	Me-QRP-00075	22.31	22.20	22.14	22.22	22.37	22.11	22.10	22.19	24.53	24.43	24.43	24.46
89	Me-QRP-00088	24.40	24.53	24.50	24.48	22.07	22.24	22.23	22.18	25.43	25.35	25.28	25.36
90	Me-QRP-00089	25.39	25.53	25.38	25.43	27.04	27.17	27.07	27.09	28.17	28.17	27.88	28.07
91	Me-QRP-00090	28.25	28.45	28.63	28.44	29.73	28.75	28.75	29.08	30.06	30.37	30.15	30.19
92	Me-QRP-00091	24.50	24.40	24.45	24.45	25.42	25.02	25.05	25.16	24.63	24.46	24.44	24.51
93	Me-QRP-00092	24.87	25.00	24.84	24.90	25.94	25.88	25.89	25.90	26.67	26.52	26.51	26.57
94	Me-QRP-00093	24.98	25.06	25.19	25.08	25.72	25.90	25.86	25.83	26.63	26.72	27.00	26.78
95	Me-QRP-00094	26.83	27.05	27.03	26.97	27.13	27.30	27.33	27.25	28.42	28.44	28.20	28.36
96	Me-QRP-00095	25.52	25.54	25.41	25.49	26.29	25.98	25.92	26.07	27.55	27.33	27.33	27.40
97	Me-QRP-00096	25.18	25.09	25.14	25.14	27.08	26.80	26.71	26.86	27.69	27.55	27.49	27.57
98	Me-QRP-00097	0.00	0.00	0.00	0.00	0.00	0.00	0.00	0.00	0.00	0.00	0.00	0.00
99	Me-QRP-00098	25.50	25.65	25.86	25.67	25.18	25.05	24.82	25.02	25.28	25.22	25.18	25.23
100	Me-QRP-00099	26.56	26.37	26.36	26.43	27.74	27.63	27.72	27.70	26.81	26.58	26.48	26.62
101	Me-QRP-00100	0.00	0.00	0.00	0.00	0.00	0.00	0.00	0.00	0.00	0.00	0.00	0.00
102	Me-QRP-00101	32.44	32.71	32.70	32.62	32.87	32.93	32.76	32.85	33.80	33.94	33.82	33.85
103	Me-QRP-00102	27.21	27.32	27.31	27.28	25.04	25.22	25.22	25.16	26.64	26.55	26.44	26.54
104	Me-QRP-00103	27.53	27.46	27.54	27.51	27.84	27.20	27.22	27.42	29.67	29.61	29.63	29.64
105	Me-QRP-00104	24.36	24.13	24.27	24.25	25.38	25.42	25.43	25.41	26.62	26.43	26.22	26.42
106	Me-QRP-00105	25.30	25.34	25.29	25.31	26.36	26.10	26.08	26.18	27.20	27.14	27.17	27.17
107	Me-QRP-00106	27.19	27.46	27.27	27.30	26.11	26.23	26.20	26.18	26.17	26.17	26.08	26.14

**Supplementary Table S8** qPCR cycles of the genes in F01 at root bulking													
	Sample	F01 leaves				F01 stems				F01 roots			
	primer ID	Ct1	Ct2	Ct3	Ave.Ct	Ct1	Ct2	Ct3	Ave.Ct	Ct1	Ct2	Ct3	Ave.Ct
1	Me-QRP-90001	20.00	20.01	19.86	19.96	22.84	23.00	23.00	22.95	21.92	22.02	21.99	21.98
2	Me-QRP-00001	22.41	22.38	22.35	22.38	24.40	24.45	24.46	24.44	24.05	24.15	24.09	24.10
3	Me-QRP-00002	24.49	24.51	24.37	24.46	25.84	25.93	26.01	25.93	23.38	23.38	23.44	23.40
4	Me-QRP-00003	0.00	0.00	0.00	0.00	0.00	0.00	0.00	0.00	0.00	0.00	0.00	0.00
5	Me-QRP-00004	27.95	28.00	28.01	27.99	26.91	27.10	27.06	27.02	23.71	23.85	23.90	23.82
6	Me-QRP-00005	22.03	21.99	21.80	21.94	22.92	23.00	23.13	23.01	23.78	23.73	23.62	23.71
7	Me-QRP-00006	24.07	24.04	23.95	24.02	25.37	25.53	25.61	25.50	25.34	25.44	25.43	25.41
8	Me-QRP-00007	23.82	23.83	23.69	23.78	25.87	26.07	26.05	25.99	26.83	26.87	26.76	26.82
9	Me-QRP-00008	23.89	23.82	23.77	23.83	26.33	26.46	26.78	26.52	25.71	25.87	25.80	25.79
10	Me-QRP-00009	28.01	28.02	28.03	28.02	28.41	28.03	28.54	28.33	25.95	25.85	25.69	25.83
11	Me-QRP-00010	29.00	29.49	29.11	29.20	31.37	31.73	31.57	31.56	29.49	30.74	30.48	30.24
12	Me-QRP-00011	23.03	23.03	22.96	23.01	24.96	25.18	25.06	25.07	24.07	24.08	24.29	24.15
13	Me-QRP-00012	19.46	19.49	19.25	19.40	24.74	24.89	24.87	24.83	27.53	27.19	27.42	27.38
14	Me-QRP-00013	22.54	22.19	22.16	22.30	24.39	24.34	24.21	24.31	25.09	25.15	25.13	25.12
15	Me-QRP-00014	21.39	21.17	21.10	21.22	22.69	22.54	22.69	22.64	23.08	23.05	23.05	23.06
16	Me-QRP-00015	23.23	22.96	22.91	23.04	23.80	23.80	23.76	23.79	23.95	23.92	23.92	23.93
17	Me-QRP-00016	23.12	23.25	23.06	23.14	23.66	23.80	23.82	23.76	24.68	24.53	24.80	24.67
18	Me-QRP-00017	21.65	21.73	21.50	21.63	22.04	23.03	22.26	22.45	22.72	22.56	22.75	22.68
19	Me-QRP-00018	24.79	24.67	24.38	24.61	23.74	23.78	23.81	23.77	24.90	24.91	25.29	25.03
20	Me-QRP-00019	26.63	26.59	26.43	26.55	25.28	25.51	25.55	25.45	26.08	25.86	26.28	26.07
21	Me-QRP-00020	23.77	23.68	23.54	23.66	26.51	26.69	26.67	26.62	26.71	26.74	27.03	26.82
22	Me-QRP-00021	28.03	27.99	28.01	28.01	30.41	30.26	30.18	30.28	30.73	30.44	30.78	30.65
23	Me-QRP-00022	21.05	21.02	20.92	21.00	23.07	23.26	23.17	23.17	24.15	24.06	24.29	24.17
24	Me-QRP-00023	23.96	24.01	23.82	23.93	25.84	26.02	26.22	26.03	26.10	25.98	26.39	26.15
25	Me-QRP-00024	26.11	26.07	25.91	26.03	26.81	26.87	26.95	26.88	25.87	25.59	25.93	25.80
26	Me-QRP-00025	33.20	32.80	32.80	32.93	33.95	34.10	33.85	33.97	31.11	31.25	31.19	31.18
27	Me-QRP-00026	27.74	27.75	27.55	27.68	27.20	27.09	27.05	27.11	26.77	26.61	26.70	26.70
28	Me-QRP-00027	23.88	23.76	23.64	23.76	25.83	25.69	25.75	25.76	25.66	25.62	25.65	25.64
29	Me-QRP-00028	31.99	31.83	31.56	31.80	34.34	35.06	34.66	34.69	34.42	34.88	34.19	34.49
30	Me-QRP-00029	0.00	0.00	0.00	0.00	0.00	0.00	0.00	0.00	0.00	0.00	0.00	0.00
31	Me-QRP-00030	26.16	26.15	26.11	26.14	28.46	28.51	28.59	28.52	29.18	29.04	29.30	29.17
32	Me-QRP-00031	26.35	26.01	26.05	26.14	29.17	29.08	29.02	29.09	28.80	28.87	28.90	28.86
33	Me-QRP-00032	24.60	24.66	24.40	24.55	26.19	26.40	26.45	26.35	24.72	24.55	24.81	24.69
34	Me-QRP-00033	23.63	23.57	23.50	23.57	23.98	24.16	24.08	24.07	24.60	24.43	24.61	24.55
35	Me-QRP-00034	31.35	31.42	31.21	31.33	31.20	31.25	31.34	31.26	31.33	30.27	31.96	31.19
36	Me-QRP-00035	0.00	0.00	0.00	0.00	0.00	0.00	0.00	0.00	0.00	0.00	0.00	0.00
37	Me-QRP-00036	0.00	0.00	0.00	0.00	0.00	0.00	0.00	0.00	0.00	0.00	0.00	0.00
38	Me-QRP-00037	29.25	29.27	29.03	29.18	30.58	30.76	30.89	30.74	30.36	30.40	30.46	30.41
39	Me-QRP-00038	26.14	26.00	25.90	26.01	28.50	28.54	28.61	28.55	29.87	29.99	29.80	29.89
40	Me-QRP-00039	0.00	0.00	0.00	0.00	0.00	0.00	0.00	0.00	0.00	0.00	0.00	0.00

41	Me-QRP-00040	33.37	34.13	33.21	33.57	35.65	35.17	35.21	35.34	34.99	34.69	35.19	34.96
42	Me-QRP-00041	24.83	25.14	24.74	24.90	24.55	24.70	24.70	24.65	23.30	22.94	23.14	23.12
43	Me-QRP-00042	28.34	28.30	28.04	28.23	30.32	30.57	30.43	30.44	30.55	30.23	30.40	30.39
44	Me-QRP-00043	22.87	22.62	22.64	22.71	24.84	24.71	24.83	24.80	24.31	24.39	24.33	24.34
45	Me-QRP-00044	0.00	0.00	0.00	0.00	0.00	0.00	0.00	0.00	0.00	0.00	0.00	0.00
46	Me-QRP-00045	23.97	23.98	23.86	23.93	25.51	25.77	25.78	25.69	26.48	26.47	26.43	26.46
47	Me-QRP-00046	26.55	26.54	26.31	26.47	29.16	29.13	29.15	29.15	30.00	29.89	29.93	29.94
48	Me-QRP-00047	23.46	23.46	23.35	23.42	25.03	25.11	25.13	25.09	25.79	25.91	25.81	25.83
49	Me-QRP-00048	27.80	27.71	27.60	27.70	34.32	33.81	33.45	33.86	34.12	34.00	34.03	34.05
50	Me-QRP-00049	27.87	27.78	27.75	27.80	28.06	28.53	28.24	28.27	26.34	26.60	26.35	26.43
51	Me-QRP-00050	27.65	27.30	27.37	27.44	34.26	34.89	33.35	34.17	31.71	32.11	31.93	31.92
52	Me-QRP-00051	0.00	0.00	0.00	0.00	0.00	0.00	0.00	0.00	0.00	0.00	0.00	0.00
53	Me-QRP-00052	25.28	25.20	25.09	25.19	26.26	26.43	26.56	26.42	24.21	24.24	24.20	24.22
54	Me-QRP-00053	25.51	25.37	25.51	25.46	18.83	18.87	18.84	18.85	24.00	24.08	24.09	24.05
55	Me-QRP-00054	21.61	21.47	21.38	21.49	26.31	26.09	26.28	26.23	26.37	26.43	26.48	26.43
56	Me-QRP-00055	21.28	21.26	21.26	21.27	26.03	26.27	26.25	26.18	26.19	25.84	26.27	26.10
57	Me-QRP-00056	25.05	25.06	24.85	24.98	28.44	28.54	28.65	28.54	26.80	26.75	26.77	26.77
58	Me-QRP-00053	25.51	25.37	25.51	25.46	18.83	18.87	18.84	18.85	24.00	24.08	24.09	24.05
59	Me-QRP-00058	21.03	21.12	21.23	21.13	22.94	23.19	23.17	23.10	22.80	22.43	22.94	22.72
60	Me-QRP-00004	27.95	28.00	28.01	27.99	26.91	27.10	27.06	27.02	23.71	23.85	23.90	23.82
61	Me-QRP-00060	22.77	22.79	22.64	22.73	25.24	25.26	25.47	25.32	25.55	25.59	25.53	25.56
62	Me-QRP-00061	20.24	20.25	20.17	20.22	24.11	24.10	24.21	24.14	24.69	24.73	24.70	24.71
63	Me-QRP-00062	0.00	0.00	0.00	0.00	0.00	0.00	0.00	0.00	0.00	0.00	0.00	0.00
64	Me-QRP-00063	0.00	0.00	0.00	0.00	0.00	0.00	0.00	0.00	0.00	0.00	0.00	0.00
65	Me-QRP-00064	26.14	25.89	25.77	25.93	27.32	27.39	27.39	27.37	27.95	27.87	27.89	27.90
66	Me-QRP-00065	24.67	24.53	24.40	24.53	26.40	26.57	26.21	26.39	27.95	27.81	27.86	27.87
67	Me-QRP-00066	0.00	0.00	0.00	0.00	0.00	0.00	0.00	0.00	0.00	0.00	0.00	0.00
68	Me-QRP-00067	0.00	0.00	0.00	0.00	0.00	0.00	0.00	0.00	0.00	0.00	0.00	0.00
69	Me-QRP-00068	0.00	0.00	0.00	0.00	0.00	0.00	0.00	0.00	0.00	0.00	0.00	0.00
70	Me-QRP-00008	23.89	23.82	23.77	23.83	26.33	26.46	26.78	26.52	25.71	25.87	25.80	25.79
71	Me-QRP-00070	0.00	0.00	0.00	0.00	0.00	0.00	0.00	0.00	0.00	0.00	0.00	0.00
72	Me-QRP-00071	22.24	22.24	22.18	22.22	22.88	23.04	23.03	22.98	23.44	23.51	23.42	23.46
73	Me-QRP-00072	0.00	0.00	0.00	0.00	0.00	0.00	0.00	0.00	0.00	0.00	0.00	0.00
74	Me-QRP-00073	24.66	24.52	24.46	24.55	25.90	25.77	25.80	25.82	25.42	25.44	25.28	25.38
75	Me-QRP-00074	26.93	26.79	26.76	26.83	29.17	29.14	29.34	29.22	27.61	27.75	27.45	27.61
76	Me-QRP-00075	21.04	20.93	20.82	20.93	22.96	22.89	22.89	22.91	23.94	24.06	23.96	23.99
77	Me-QRP-00076	23.83	23.56	23.45	23.61	26.46	26.38	26.68	26.51	25.17	25.17	24.99	25.11
78	Me-QRP-00077	23.23	23.20	23.18	23.20	24.86	25.01	24.95	24.94	25.30	25.47	25.37	25.38
79	Me-QRP-00078	24.15	24.23	24.08	24.15	25.67	25.89	25.90	25.82	25.44	25.54	25.60	25.52
80	Me-QRP-00079	24.15	24.23	24.08	24.15	25.67	25.89	25.90	25.82	25.44	25.54	25.60	25.52
81	Me-QRP-00080	27.94	27.87	27.76	27.86	29.16	29.18	29.05	29.13	28.99	29.04	29.25	29.09
82	Me-QRP-00081	25.10	24.55	24.88	24.84	26.44	26.10	27.56	26.70	25.71	25.29	26.50	25.83
83	Me-QRP-00082	24.84	24.73	24.63	24.73	27.29	27.22	27.37	27.30	26.02	25.86	25.81	25.89

84	Me-QRP-00083	24.18	24.16	24.05	24.13	24.99	24.90	24.87	24.92	24.99	24.91	24.91	24.94
85	Me-QRP-00084	23.59	23.48	23.33	23.47	26.94	26.84	26.86	26.88	26.63	26.25	26.21	26.36
86	Me-QRP-00085	23.46	23.53	23.35	23.45	26.16	26.07	26.06	26.10	23.84	23.84	23.75	23.81
87	Me-QRP-00086	22.22	22.13	22.02	22.12	24.05	23.73	24.06	23.95	24.87	24.84	24.81	24.84
88	Me-QRP-00075	21.04	20.93	20.82	20.93	22.96	22.89	22.89	22.91	23.94	24.06	23.96	23.99
89	Me-QRP-00088	22.59	22.54	22.43	22.52	23.84	23.75	23.71	23.77	23.88	23.78	23.77	23.81
90	Me-QRP-00089	23.99	23.81	23.79	23.86	27.93	28.00	27.89	27.94	27.45	27.31	27.50	27.42
91	Me-QRP-00090	26.70	26.63	26.51	26.61	29.24	29.00	29.49	29.24	29.11	29.07	29.10	29.09
92	Me-QRP-00091	23.60	23.33	23.29	23.40	25.34	25.27	25.54	25.38	24.81	24.82	24.84	24.82
93	Me-QRP-00092	23.38	23.30	23.18	23.29	26.79	26.67	26.58	26.68	25.08	25.21	25.05	25.11
94	Me-QRP-00093	23.42	23.48	23.33	23.41	26.61	26.65	26.73	26.66	25.13	25.12	25.09	25.11
95	Me-QRP-00094	24.98	24.90	24.82	24.90	26.68	28.35	28.25	28.30	27.23	26.60	26.88	26.90
96	Me-QRP-00095	23.73	23.47	23.46	23.55	26.95	27.09	26.81	26.95	26.43	26.58	26.39	26.47
97	Me-QRP-00096	23.78	23.58	23.53	23.63	27.11	27.16	27.18	27.15	26.60	26.59	37.04	30.08
98	Me-QRP-00097	0.00	0.00	0.00	0.00	0.00	0.00	0.00	0.00	0.00	0.00	0.00	0.00
99	Me-QRP-00098	24.36	24.20	24.19	24.25	25.89	25.85	25.89	25.88	24.31	24.38	24.31	24.33
100	Me-QRP-00099	27.07	27.10	27.17	27.11	26.22	25.91	25.94	26.02	27.27	27.17	27.16	27.20
101	Me-QRP-00100	0.00	0.00	0.00	0.00	0.00	0.00	0.00	0.00	0.00	0.00	0.00	0.00
102	Me-QRP-00101	29.56	29.56	29.39	29.50	31.85	31.75	31.65	31.75	30.75	30.53	30.69	30.66
103	Me-QRP-00102	25.21	25.01	24.90	25.04	26.67	26.58	26.52	26.59	25.81	25.71	25.70	25.74
104	Me-QRP-00103	25.96	25.77	25.76	25.83	28.78	28.64	28.53	28.65	27.84	27.81	27.93	27.86
105	Me-QRP-00104	23.89	23.73	23.65	23.76	26.49	26.28	26.32	26.36	25.91	25.82	25.82	25.85
106	Me-QRP-00105	24.29	23.97	23.95	24.07	26.97	26.96	26.93	26.95	25.71	25.73	25.73	25.72
107	Me-QRP-00106	25.89	25.76	25.71	25.79	27.01	27.06	26.99	27.02	26.18	26.14	26.07	26.13

Supplementary Table S9 qPCR cycles of the genes in H124 at root maturity

	Sample	H124 leaves				H124 stems				H124 roots			
	primer ID	Ct1	Ct2	Ct3	Ave.Ct	Ct1	Ct2	Ct3	Ave.Ct	Ct1	Ct2	Ct3	Ave.Ct
1	Me-QRP-90001	21.34	21.43	21.50	21.42	22.13	22.16	22.24	22.18	23.51	23.55	23.74	23.60
2	Me-QRP-00001	22.19	22.53	22.12	22.28	24.65	24.78	24.71	24.71	25.86	25.92	25.87	25.88
3	Me-QRP-00002	24.99	25.24	25.23	25.15	23.96	23.96	24.01	23.98	26.09	26.01	26.20	26.10
4	Me-QRP-00003	0.00	0.00	0.00	0.00	0.00	0.00	0.00	0.00	0.00	0.00	0.00	0.00
5	Me-QRP-00004	28.82	28.97	28.56	28.78	25.85	25.88	25.89	25.87	26.21	26.20	26.26	26.22
6	Me-QRP-00005	23.89	23.98	23.89	23.92	23.22	23.30	23.34	23.29	24.19	24.29	24.69	24.39
7	Me-QRP-00006	25.80	25.92	25.72	25.81	24.46	24.50	24.52	24.50	26.82	26.95	27.03	26.93
8	Me-QRP-00007	24.45	24.68	24.56	24.56	25.30	25.30	25.37	25.33	27.49	27.41	27.52	27.47
9	Me-QRP-00008	25.74	25.75	25.74	25.74	25.86	25.94	25.93	25.91	27.66	27.70	27.77	27.71
10	Me-QRP-00009	24.97	24.90	24.84	24.90	27.32	27.25	27.52	27.36	27.67	27.57	27.41	27.55
11	Me-QRP-00010	28.97	29.12	29.06	29.05	29.89	29.85	29.92	29.88	31.03	31.26	31.32	31.20
12	Me-QRP-00011	23.34	23.48	23.44	23.42	23.68	23.79	23.77	23.75	25.83	25.85	25.93	25.87
13	Me-QRP-00012	20.82	21.23	21.10	21.05	25.62	25.77	25.74	25.71	27.79	27.92	27.95	27.89
14	Me-QRP-00013	25.17	25.14	25.26	25.19	23.96	23.71	23.72	23.80	22.87	22.89	22.92	22.89
15	Me-QRP-00014	23.52	23.54	23.52	23.53	23.44	23.21	23.26	23.31	23.98	23.97	23.92	23.95
16	Me-QRP-00015	24.85	24.85	24.93	24.88	24.18	24.05	24.11	24.11	25.67	25.76	25.71	25.71
17	Me-QRP-00016	24.24	24.54	24.42	24.40	23.88	23.95	23.99	23.94	25.30	25.33	25.52	25.38
18	Me-QRP-00017	24.14	24.43	24.25	24.27	22.74	22.79	22.77	22.77	23.95	24.91	24.84	24.57
19	Me-QRP-00018	27.00	27.13	27.14	27.09	24.42	24.57	24.56	24.52	25.60	25.47	25.80	25.62
20	Me-QRP-00019	27.00	27.18	27.07	27.08	28.00	28.17	28.16	28.11	27.02	27.18	27.32	27.17
21	Me-QRP-00020	25.41	25.62	25.51	25.51	26.13	26.13	26.07	26.11	N/A	27.94	27.99	27.97
22	Me-QRP-00021	28.52	28.77	28.59	28.63	29.60	29.60	29.82	29.67	32.08	32.14	32.55	32.26
23	Me-QRP-00022	24.33	24.54	24.31	24.39	23.95	23.98	23.96	23.96	24.86	24.88	24.98	24.90
24	Me-QRP-00023	25.09	25.18	25.02	25.10	24.94	24.96	24.92	24.94	27.32	27.36	27.62	27.43
25	Me-QRP-00024	27.13	27.29	27.24	27.22	26.73	26.80	26.68	26.74	28.12	28.14	28.31	28.19
26	Me-QRP-00025	33.89	34.25	34.23	34.12	32.85	33.44	32.92	33.07	34.80	34.85	34.38	34.68
27	Me-QRP-00026	28.80	28.64	29.03	28.82	29.26	29.04	29.32	29.21	27.72	27.62	27.63	27.66
28	Me-QRP-00027	24.66	24.59	24.77	24.67	26.16	25.92	25.88	25.99	27.80	27.76	27.65	27.74
29	Me-QRP-00028	33.10	32.71	32.74	32.85	35.62	35.98	35.55	35.72	36.19	37.64	36.36	36.73
30	Me-QRP-00029	0.00	0.00	0.00	0.00	0.00	0.00	0.00	0.00	0.00	0.00	0.00	0.00
31	Me-QRP-00030	27.39	27.55	27.35	27.43	28.52	28.59	28.52	28.54	30.89	30.90	31.02	30.94
32	Me-QRP-00031	27.25	27.11	27.26	27.20	28.57	28.34	28.30	28.40	30.31	30.84	30.32	30.49
33	Me-QRP-00032	25.85	25.96	25.86	25.89	25.80	25.86	25.97	25.88	26.71	26.78	26.92	26.80
34	Me-QRP-00033	27.56	27.73	27.58	27.62	26.17	26.21	26.18	26.19	26.58	26.64	26.61	26.61
35	Me-QRP-00034	32.22	32.79	32.34	32.45	31.46	31.37	31.47	31.43	31.73	31.42	31.75	31.63
36	Me-QRP-00035	0.00	0.00	0.00	0.00	0.00	0.00	0.00	0.00	0.00	0.00	0.00	0.00
37	Me-QRP-00036	0.00	0.00	0.00	0.00	0.00	0.00	0.00	0.00	0.00	0.00	0.00	0.00
38	Me-QRP-00037	29.48	29.55	29.48	29.50	29.92	30.04	29.98	29.98	31.82	32.11	31.90	31.94
39	Me-QRP-00038	27.12	27.08	27.20	27.13	28.16	28.09	28.15	28.13	30.67	30.75	30.56	30.66

40	Me-QRP-00039	0.00	0.00	0.00	0.00	0.00	0.00	0.00	0.00	0.00	0.00	0.00	0.00
41	Me-QRP-00040	34.41	34.01	34.68	34.37	35.73	34.42	34.42	34.86	37.68	37.80	36.69	37.39
42	Me-QRP-00041	24.46	24.46	24.51	24.48	21.98	22.02	21.97	21.99	22.47	22.74	22.72	22.64
43	Me-QRP-00042	29.62	29.76	29.64	29.67	30.32	30.22	30.19	30.24	31.91	31.88	31.93	31.91
44	Me-QRP-00043	24.12	24.07	24.13	24.11	24.34	24.17	24.12	24.21	26.03	26.05	26.10	26.06
45	Me-QRP-00044	0.00	0.00	0.00	0.00	0.00	0.00	0.00	0.00	0.00	0.00	0.00	0.00
46	Me-QRP-00045	23.85	24.17	23.99	24.00	25.24	25.29	25.33	25.29	28.11	28.08	28.23	28.14
47	Me-QRP-00046	26.31	26.55	26.63	26.50	29.81	29.82	29.93	29.85	31.23	31.32	32.09	31.55
48	Me-QRP-00047	25.56	25.76	25.60	25.64	25.77	25.91	25.79	25.82	26.19	26.14	26.21	26.18
49	Me-QRP-00048	29.28	29.15	29.18	29.21	35.40	35.40	35.73	35.51	37.53	37.60	N/A	#VALUE!
50	Me-QRP-00049	29.02	29.18	29.28	29.16	28.22	28.24	28.22	28.23	27.91	27.80	27.89	27.87
51	Me-QRP-00050	28.54	28.39	28.58	28.50	N/A	34.06	33.55	33.81	32.83	32.82	32.46	32.70
52	Me-QRP-00051	0.00	0.00	0.00	0.00	0.00	0.00	0.00	0.00	0.00	0.00	0.00	0.00
53	Me-QRP-00052	26.21	26.42	26.29	26.31	25.46	25.69	25.54	25.56	25.01	25.17	25.15	25.11
54	Me-QRP-00053	23.91	23.89	23.87	23.89	21.55	21.69	21.58	21.61	24.20	24.20	24.16	24.19
55	Me-QRP-00054	24.33	24.50	24.55	24.46	25.84	25.59	25.04	25.49	27.75	27.76	27.59	27.70
56	Me-QRP-00055	24.10	24.31	24.13	24.18	25.70	25.82	25.80	25.78	27.25	27.33	27.39	27.32
57	Me-QRP-00056	26.12	26.33	26.21	26.22	27.54	27.44	27.72	27.57	28.10	27.89	28.11	28.04
58	Me-QRP-00053	23.91	23.89	23.87	23.89	21.55	21.69	21.58	21.61	24.20	24.20	24.16	24.19
59	Me-QRP-00058	19.74	19.78	19.77	19.76	24.16	24.77	24.95	24.63	21.78	21.89	21.89	21.85
60	Me-QRP-00004	28.82	28.97	28.56	28.78	25.85	25.88	25.89	25.87	26.21	26.20	26.26	26.22
61	Me-QRP-00060	25.75	25.90	25.83	25.83	25.72	25.75	25.80	25.76	26.56	26.59	26.78	26.64
62	Me-QRP-00061	22.99	23.37	23.14	23.17	24.11	24.17	24.01	24.10	25.22	25.11	25.15	25.16
63	Me-QRP-00062	0.00	0.00	0.00	0.00	0.00	0.00	0.00	0.00	0.00	0.00	0.00	0.00
64	Me-QRP-00063	0.00	0.00	0.00	0.00	0.00	0.00	0.00	0.00	0.00	0.00	0.00	0.00
65	Me-QRP-00064	26.30	26.29	26.29	26.29	29.92	29.78	29.89	29.86	30.08	29.87	29.84	29.93
66	Me-QRP-00065	25.30	25.31	25.43	25.35	26.88	26.93	26.70	26.84	29.79	29.80	29.79	29.79
67	Me-QRP-00066	0.00	0.00	0.00	0.00	0.00	0.00	0.00	0.00	0.00	0.00	0.00	0.00
68	Me-QRP-00067	0.00	0.00	0.00	0.00	0.00	0.00	0.00	0.00	0.00	0.00	0.00	0.00
69	Me-QRP-00068	0.00	0.00	0.00	0.00	0.00	0.00	0.00	0.00	0.00	0.00	0.00	0.00
70	Me-QRP-00008	25.74	25.75	25.74	25.74	25.86	25.94	25.93	25.91	27.66	27.70	27.77	27.71
71	Me-QRP-00070	0.00	0.00	0.00	0.00	0.00	0.00	0.00	0.00	0.00	0.00	0.00	0.00
72	Me-QRP-00071	25.68	25.94	25.72	25.78	23.77	23.99	23.84	23.87	25.19	24.94	25.05	25.06
73	Me-QRP-00072	0.00	0.00	0.00	0.00	0.00	0.00	0.00	0.00	0.00	0.00	0.00	0.00
74	Me-QRP-00073	25.76	25.52	25.88	25.72	25.17	25.06	25.07	25.10	26.46	26.44	26.56	26.49
75	Me-QRP-00074	27.31	27.36	27.31	27.33	28.61	28.62	28.60	28.61	29.46	29.88	29.58	29.64
76	Me-QRP-00075	22.55	22.56	22.82	22.64	23.65	23.69	23.65	23.66	24.30	24.41	24.41	24.37
77	Me-QRP-00076	24.52	24.40	24.59	24.51	26.29	26.17	26.23	26.23	28.08	28.08	28.17	28.11
78	Me-QRP-00077	25.01	25.21	25.11	25.11	24.79	24.85	24.72	24.79	26.53	26.64	26.71	26.62
79	Me-QRP-00078	25.00	25.29	25.23	25.18	25.79	25.88	25.89	25.86	27.91	27.96	27.96	27.94
80	Me-QRP-00079	25.00	25.29	25.23	25.18	25.79	25.88	25.89	25.86	27.91	27.96	27.96	27.94
81	Me-QRP-00080	28.69	28.56	28.72	28.66	28.20	28.02	28.11	28.11	29.56	29.47	29.69	29.57
82	Me-QRP-00081	25.75	26.22	26.20	26.06	27.79	27.64	27.58	27.67	27.15	28.78	27.88	27.93

83	Me-QRP-00082	25.55	25.69	25.81	25.68	27.12	26.98	27.10	27.06	28.58	28.72	28.92	28.74
84	Me-QRP-00083	24.99	25.04	24.99	25.01	25.57	25.46	25.46	25.50	26.31	26.39	26.39	26.36
85	Me-QRP-00084	24.69	24.77	24.74	24.73	26.12	26.05	25.91	26.02	28.59	28.61	28.61	28.60
86	Me-QRP-00085	24.64	24.75	24.73	24.71	25.46	25.33	25.29	25.36	26.88	26.96	26.94	26.93
87	Me-QRP-00086	24.53	24.46	24.54	24.51	24.66	24.66	24.65	24.66	25.32	25.43	25.45	25.40
88	Me-QRP-00075	22.55	22.56	22.82	22.64	23.65	23.69	23.65	23.66	24.30	24.41	24.41	24.37
89	Me-QRP-00088	23.69	23.92	24.08	23.90	22.47	22.33	22.34	22.38	23.53	23.50	23.47	23.50
90	Me-QRP-00089	24.31	24.29	24.42	24.34	26.32	26.14	25.95	26.14	30.34	30.73	30.34	30.47
91	Me-QRP-00090	27.14	27.28	27.35	27.26	28.38	28.15	28.14	28.22	30.56	30.57	30.63	30.59
92	Me-QRP-00091	23.72	23.75	23.59	23.69	25.15	25.06	25.06	25.09	26.46	26.25	26.51	26.41
93	Me-QRP-00092	24.40	24.33	24.55	24.43	26.30	26.28	26.22	26.27	27.76	27.84	27.80	27.80
94	Me-QRP-00093	24.26	24.31	24.49	24.36	26.02	25.93	25.94	25.96	27.08	27.18	26.94	27.07
95	Me-QRP-00094	26.92	27.16	27.29	27.12	27.14	27.13	26.94	27.07	28.84	28.92	28.84	28.86
96	Me-QRP-00095	24.38	N/A	24.53	######	25.51	25.50	25.44	25.48	27.90	27.97	27.93	27.93
97	Me-QRP-00096	25.26	25.37	25.44	25.36	26.64	26.61	26.75	26.67	28.99	29.10	29.20	29.10
98	Me-QRP-00097	0.00	0.00	0.00	0.00	0.00	0.00	0.00	0.00	0.00	0.00	0.00	0.00
99	Me-QRP-00098	24.92	24.96	25.05	24.97	25.97	25.97	25.91	25.95	26.95	26.99	26.99	26.98
100	Me-QRP-00099	26.30	26.26	26.22	26.26	26.25	33.25	26.38	26.32	26.88	26.91	26.87	26.89
101	Me-QRP-00100	0.00	0.00	0.00	0.00	0.00	0.00	0.00	0.00	0.00	0.00	0.00	0.00
102	Me-QRP-00101	32.87	32.94	32.59	32.80	33.14	32.89	32.92	32.99	34.52	35.03	34.46	34.67
103	Me-QRP-00102	27.32	26.84	26.94	27.03	25.18	24.96	24.97	25.04	27.53	26.88	26.83	27.08
104	Me-QRP-00103	25.96	25.89	25.90	25.92	26.88	26.76	26.74	26.79	27.59	27.65	27.46	27.57
105	Me-QRP-00104	25.39	25.56	25.74	25.56	25.84	25.58	25.63	25.68	27.84	27.85	27.89	27.86
106	Me-QRP-00105	25.05	25.04	25.06	25.05	25.88	25.76	25.64	25.76	28.10	28.03	28.03	28.05
107	Me-QRP-00106	27.20	27.13	27.15	27.16	26.60	26.45	26.29	26.45	28.28	28.38	28.23	28.29

Supplementary Table S10 qPCR cycles of the genes in F01 at root maturity

	Sample	F01 leaves				F01 stems				F01 roots			
	primer ID	Ct1	Ct2	Ct3	Ave.Ct	Ct1	Ct2	Ct3	Ave.Ct	Ct1	Ct2	Ct3	Ave.Ct
1	Me-QRP-90001	21.00	21.05	21.26	21.10	23.47	23.50	23.79	23.59	22.84	22.88	23.05	22.92
2	Me-QRP-00001	20.74	20.86	20.92	20.84	25.42	25.42	25.79	25.54	24.56	24.74	24.61	24.64
3	Me-QRP-00002	24.23	24.16	24.42	24.27	25.82	25.79	26.04	25.89	24.62	24.62	24.65	24.63
4	Me-QRP-00003	0.00	0.00	0.00	0.00	0.00	0.00	0.00	0.00	0.00	0.00	0.00	0.00
5	Me-QRP-00004	26.78	26.89	27.01	26.89	27.32	27.30	27.67	27.43	25.06	25.08	25.06	25.07
6	Me-QRP-00005	23.07	23.17	23.26	23.17	23.47	23.38	23.99	23.62	23.10	23.08	23.23	23.14
7	Me-QRP-00006	24.20	24.32	24.57	24.37	26.09	25.97	26.33	26.13	25.91	25.97	26.02	25.97
8	Me-QRP-00007	24.59	24.71	24.94	24.75	26.64	26.66	26.96	26.75	26.63	26.55	26.57	26.58
9	Me-QRP-00008	24.86	25.11	25.24	25.07	27.01	27.02	27.23	27.09	26.66	26.76	26.79	26.74
10	Me-QRP-00009	27.71	27.79	27.84	27.78	N/A	37.67	37.40	37.54	28.30	27.95	27.92	28.06
11	Me-QRP-00010	30.03	29.27	29.73	29.68	30.85	31.22	31.21	31.09	30.33	30.54	30.25	30.37
12	Me-QRP-00011	22.61	22.64	22.98	22.74	25.26	25.65	26.05	25.65	24.66	24.58	24.60	24.61
13	Me-QRP-00012	19.83	19.98	20.33	20.05	25.64	25.91	26.29	25.95	27.74	27.67	27.83	27.75
14	Me-QRP-00013	25.47	24.96	25.10	25.18	24.12	24.23	24.19	24.18	24.73	24.62	24.64	24.66
15	Me-QRP-00014	22.53	22.50	22.56	22.53	23.25	23.45	23.46	23.39	23.13	22.97	22.96	23.02
16	Me-QRP-00015	24.15	23.59	23.74	23.82	24.89	24.88	24.88	24.88	24.04	23.98	24.02	24.01
17	Me-QRP-00016	24.13	24.21	24.72	24.35	24.46	24.89	25.30	24.88	24.21	24.27	24.25	24.24
18	Me-QRP-00017	21.79	22.05	22.32	22.05	22.86	23.17	23.91	23.31	22.37	22.55	22.47	22.46
19	Me-QRP-00018	24.82	24.92	25.44	25.06	24.57	25.21	25.77	25.18	23.77	23.84	23.91	23.84
20	Me-QRP-00019	25.96	25.98	26.40	26.11	26.96	27.25	27.72	27.31	25.70	25.73	25.78	25.74
21	Me-QRP-00020	24.63	24.82	25.09	24.84	27.39	27.91	28.15	27.82	27.03	27.09	27.03	27.05
22	Me-QRP-00021	28.33	28.67	28.97	28.66	30.79	31.30	31.49	31.19	30.15	30.31	30.70	30.39
23	Me-QRP-00022	22.09	22.25	22.64	22.33	23.20	23.51	23.96	23.56	23.26	23.21	23.21	23.23
24	Me-QRP-00023	24.79	24.85	25.27	24.97	26.63	27.12	27.60	27.12	26.43	26.50	26.53	26.48
25	Me-QRP-00024	25.91	25.96	26.45	26.11	27.81	28.16	28.48	28.15	26.31	26.32	26.51	26.38
26	Me-QRP-00025	33.83	34.10	33.50	33.81	34.14	34.98	34.91	34.68	33.91	33.90	34.01	33.94
27	Me-QRP-00026	27.46	27.35	27.41	27.41	30.16	30.64	30.64	30.48	26.47	26.51	26.49	26.49
28	Me-QRP-00027	23.94	23.89	23.95	23.93	26.29	26.39	26.39	26.36	26.66	26.65	26.62	26.65
29	Me-QRP-00028	33.58	33.15	33.22	33.31	36.67	35.81	36.92	36.47	36.09	35.22	35.71	35.67
30	Me-QRP-00029	0.00	0.00	0.00	0.00	0.00	0.00	0.00	0.00	0.00	0.00	0.00	0.00
31	Me-QRP-00030	26.75	26.87	27.08	26.90	28.91	29.12	29.44	29.16	29.25	29.20	29.28	29.24
32	Me-QRP-00031	27.66	27.14	27.52	27.44	29.57	29.58	29.76	29.64	29.42	29.55	29.57	29.51
33	Me-QRP-00032	25.50	25.67	25.87	25.68	26.95	27.04	27.51	27.17	25.94	26.00	25.98	25.97
34	Me-QRP-00033	24.95	25.18	25.26	25.13	25.21	25.20	25.56	25.32	25.13	25.11	25.12	25.12
35	Me-QRP-00034	31.26	31.63	32.00	31.63	29.01	28.75	31.19	29.65	30.83	31.27	30.80	30.97
36	Me-QRP-00035	0.00	0.00	0.00	0.00	0.00	0.00	0.00	0.00	0.00	0.00	0.00	0.00
37	Me-QRP-00036	0.00	0.00	0.00	0.00	0.00	0.00	0.00	0.00	0.00	0.00	0.00	0.00
38	Me-QRP-00037	29.72	29.82	29.99	29.84	30.92	31.10	31.39	31.14	30.92	30.86	30.88	30.89
39	Me-QRP-00038	27.96	27.63	27.77	27.79	29.11	29.35	29.43	29.30	30.64	30.23	30.17	30.34

40	Me-QRP-00039	0.00	0.00	0.00	0.00	0.00	0.00	0.00	0.00	0.00	0.00	0.00	0.00
41	Me-QRP-00040	35.06	35.07	34.73	34.95	34.79	36.64	36.35	35.92	36.42	35.35	34.28	35.35
42	Me-QRP-00041	23.66	23.82	23.92	23.80	23.90	23.81	24.14	23.95	22.11	22.12	22.14	22.12
43	Me-QRP-00042	29.92	29.84	30.09	29.95	31.09	30.93	31.11	31.04	30.81	30.89	30.87	30.86
44	Me-QRP-00043	24.24	23.82	23.95	24.00	25.46	25.64	25.66	25.59	24.95	24.96	24.98	24.97
45	Me-QRP-00044	0.00	0.00	0.00	0.00	0.00	0.00	0.00	0.00	0.00	0.00	0.00	0.00
46	Me-QRP-00045	23.80	23.73	23.92	23.82	26.27	26.09	26.46	26.27	26.72	26.72	26.74	26.73
47	Me-QRP-00046	26.11	26.04	26.32	26.16	29.47	29.34	29.84	29.55	31.08	30.89	31.07	31.01
48	Me-QRP-00047	24.92	24.96	25.13	25.01	25.48	25.38	25.76	25.54	25.18	25.20	25.33	25.24
49	Me-QRP-00048	29.18	28.92	28.91	29.00	34.23	33.59	34.57	34.13	33.58	33.42	33.92	33.64
50	Me-QRP-00049	27.99	28.81	28.93	28.57	29.43	29.28	29.57	29.43	27.42	27.34	27.34	27.37
51	Me-QRP-00050	29.74	28.82	28.98	29.18	34.79	34.46	33.64	34.30	30.80	30.70	30.60	30.70
52	Me-QRP-00051	0.00	0.00	0.00	0.00	0.00	0.00	0.00	0.00	0.00	0.00	0.00	0.00
53	Me-QRP-00052	25.96	26.03	26.20	26.06	26.94	27.08	27.37	27.13	24.64	24.52	24.76	24.64
54	Me-QRP-00053	24.48	24.56	24.59	24.54	21.16	21.18	21.36	21.23	25.07	25.07	25.19	25.11
55	Me-QRP-00054	24.00	23.92	23.96	23.96	26.30	26.61	26.61	26.51	26.40	26.47	26.47	26.44
56	Me-QRP-00055	23.62	23.75	23.83	23.73	26.57	26.39	26.45	26.47	26.36	26.47	26.49	26.44
57	Me-QRP-00056	26.50	26.49	26.82	26.61	28.93	28.86	29.15	28.98	27.81	27.75	27.89	27.82
58	Me-QRP-00053	24.48	24.56	24.59	24.54	21.16	21.18	21.36	21.23	25.07	25.07	25.19	25.11
59	Me-QRP-00058	21.18	21.49	21.50	21.39	23.72	23.92	24.33	23.99	23.50	22.49	23.84	23.28
60	Me-QRP-00004	26.78	26.89	27.01	26.89	27.32	27.30	27.67	27.43	25.06	25.08	25.06	25.07
61	Me-QRP-00060	24.04	24.05	24.37	24.16	25.27	25.21	25.56	25.35	25.48	25.66	25.68	25.61
62	Me-QRP-00061	21.98	22.01	22.10	22.03	24.66	24.13	24.43	24.41	24.84	24.66	24.12	24.54
63	Me-QRP-00062	0.00	0.00	0.00	0.00	0.00	0.00	0.00	0.00	0.00	0.00	0.00	0.00
64	Me-QRP-00063	0.00	0.00	0.00	0.00	0.00	0.00	0.00	0.00	0.00	0.00	0.00	0.00
65	Me-QRP-00064	26.07	26.00	26.13	26.07	29.33	29.25	29.47	29.35	30.12	30.18	30.13	30.14
66	Me-QRP-00065	24.74	24.82	24.84	24.80	26.50	26.69	26.67	26.62	28.68	28.88	28.74	28.77
67	Me-QRP-00066	0.00	0.00	0.00	0.00	0.00	0.00	0.00	0.00	0.00	0.00	0.00	0.00
68	Me-QRP-00067	0.00	0.00	0.00	0.00	0.00	0.00	0.00	0.00	0.00	0.00	0.00	0.00
69	Me-QRP-00068	0.00	0.00	0.00	0.00	0.00	0.00	0.00	0.00	0.00	0.00	0.00	0.00
70	Me-QRP-00008	24.86	25.11	25.24	25.07	27.01	27.02	27.23	27.09	26.66	26.76	26.79	26.74
71	Me-QRP-00070	0.00	0.00	0.00	0.00	0.00	0.00	0.00	0.00	0.00	0.00	0.00	0.00
72	Me-QRP-00071	23.53	23.48	23.68	23.56	24.01	23.94	24.26	24.07	23.61	23.75	23.70	23.69
73	Me-QRP-00072	0.00	0.00	0.00	0.00	0.00	0.00	0.00	0.00	0.00	0.00	0.00	0.00
74	Me-QRP-00073	24.96	24.89	24.89	24.91	26.36	26.65	26.66	26.56	26.39	25.36	25.44	25.73
75	Me-QRP-00074	27.72	27.29	27.72	27.58	30.46	30.31	30.27	30.34	28.83	28.76	28.65	28.75
76	Me-QRP-00075	22.90	22.50	22.76	22.72	24.61	24.78	24.79	24.72	23.50	23.57	23.59	23.55
77	Me-QRP-00076	24.99	24.69	24.75	24.81	26.70	26.87	26.82	26.80	26.87	26.99	27.14	27.00
78	Me-QRP-00077	23.76	23.76	23.94	23.82	25.78	25.71	25.98	25.82	25.79	25.85	25.87	25.84
79	Me-QRP-00078	25.12	25.07	25.38	25.19	26.78	26.83	27.14	26.92	26.74	26.83	26.88	26.82
80	Me-QRP-00079	25.12	25.07	25.38	25.19	26.78	26.83	27.14	26.92	26.74	26.83	26.88	26.82
81	Me-QRP-00080	28.02	27.88	28.17	28.03	29.72	30.04	29.85	29.87	28.53	28.69	28.54	28.58
82	Me-QRP-00081	26.71	25.75	26.07	26.18	27.76	27.85	28.20	27.94	27.38	28.13	26.86	27.46

83	Me-QRP-00082	26.15	25.77	25.86	25.93	27.50	27.72	27.74	27.65	27.84	27.82	27.70	27.79
84	Me-QRP-00083	24.26	24.28	24.25	24.26	25.59	25.83	25.89	25.77	25.32	25.35	25.39	25.35
85	Me-QRP-00084	24.14	24.16	24.19	24.16	27.08	27.24	27.33	27.22	27.73	27.71	27.59	27.68
86	Me-QRP-00085	24.07	24.13	24.07	24.09	26.30	26.48	26.43	26.40	25.79	25.82	25.84	25.82
87	Me-QRP-00086	24.27	23.88	24.00	24.05	25.23	25.44	25.42	25.36	24.43	24.51	24.41	24.45
88	Me-QRP-00075	22.90	22.50	22.76	22.72	24.61	24.78	24.79	24.72	23.50	23.57	23.59	23.55
89	Me-QRP-00088	23.38	23.41	23.39	23.39	23.51	23.71	23.72	23.64	22.18	22.15	22.14	22.16
90	Me-QRP-00089	24.09	24.12	24.12	24.11	27.47	27.60	27.49	27.52	28.81	28.86	28.85	28.84
91	Me-QRP-00090	27.83	27.52	27.67	27.68	29.16	29.59	29.33	29.36	29.69	29.53	29.56	29.59
92	Me-QRP-00091	25.22	24.74	24.99	24.98	26.77	26.87	26.89	26.84	25.17	25.59	25.14	25.30
93	Me-QRP-00092	23.98	23.85	23.88	23.90	27.16	27.52	27.39	27.36	26.95	26.89	26.93	26.92
94	Me-QRP-00093	24.08	23.86	23.87	23.94	27.13	27.25	27.26	27.22	26.34	26.47	26.33	26.38
95	Me-QRP-00094	25.94	25.99	26.02	25.98	28.37	28.47	28.38	28.41	28.16	28.18	28.16	28.17
96	Me-QRP-00095	25.03	24.67	24.75	24.82	26.76	26.87	26.93	26.85	27.26	27.24	27.27	27.26
97	Me-QRP-00096	25.18	24.74	24.94	24.95	27.82	27.79	27.96	27.86	27.81	27.81	27.78	27.80
98	Me-QRP-00097	0.00	0.00	0.00	0.00	0.00	0.00	0.00	0.00	0.00	0.00	0.00	0.00
99	Me-QRP-00098	24.91	24.48	24.55	24.65	26.46	26.64	26.63	26.58	25.71	25.65	25.61	25.66
100	Me-QRP-00099	28.11	28.29	28.01	28.14	26.79	26.38	26.42	26.53	28.20	28.12	28.04	28.12
101	Me-QRP-00100	0.00	0.00	0.00	0.00	0.00	0.00	0.00	0.00	0.00	0.00	0.00	0.00
102	Me-QRP-00101	31.38	31.38	31.37	31.38	32.76	32.82	32.85	32.81	32.90	33.06	32.79	32.92
103	Me-QRP-00102	26.38	26.35	26.43	26.38	26.55	26.78	26.77	26.70	26.47	26.50	26.49	26.49
104	Me-QRP-00103	26.65	26.08	26.35	26.36	28.45	28.56	28.66	28.56	27.59	27.56	27.54	27.56
105	Me-QRP-00104	23.78	23.65	23.79	23.74	26.78	26.94	26.97	26.90	26.82	26.86	26.92	26.87
106	Me-QRP-00105	25.10	24.69	24.87	24.89	26.98	27.15	27.04	27.06	26.86	26.98	26.88	26.90
107	Me-QRP-00106	25.83	25.79	25.86	25.83	26.72	26.81	26.78	26.77	27.23	27.32	27.27	27.27
